# Synthesis and biological investigation of (+)-3-hydroxymethylartemisinin

**DOI:** 10.3762/bjoc.15.51

**Published:** 2019-02-27

**Authors:** Toni Smeilus, Farnoush Mousavizadeh, Johannes Krieger, Xingzhao Tu, Marcel Kaiser, Athanassios Giannis

**Affiliations:** 1 Faculty of Chemistry and Mineralogy, Institute of Organic Chemistry, University of Leipzig, Johannisallee 29, 04301 Leipzig, Germany; 2 Dr. M. Kaiser Swiss Tropical and Public Health Institute, Socinstrasse 57, 4051 Basel, Switzerland; 3 University of Basel, Petersplatz 1, 4003 Basel, Switzerland

**Keywords:** artemisinin, biomimetic synthesis, Diels–Alder reaction, malaria, peroxides

## Abstract

Herein, we describe a biomimetic entry to (+)-3-hydroxymethylartemisinin (**2**) as well as to the artemisinin derivatives (+)-3-hydroxymethyl-9-desmethylartemisinin (**16**) and (+)-3-hydroxymethyl-9-*epi*-artemisinin (**18**), starting from the known and readily available chiral aldehyde **3** and alkyne **4**. Subsequently, the synthesized compounds have been evaluated for their antimalarial activity against the drug-sensitive *P. falciparum* NF54 strain. All of them were inactive. In addition, they did not show any toxicity against L6 cells (a primary cell line derived from rat skeletal myoblasts). These results contribute to a better understanding of artemisinins mechanism of action.

## Introduction

**Figure 1:**
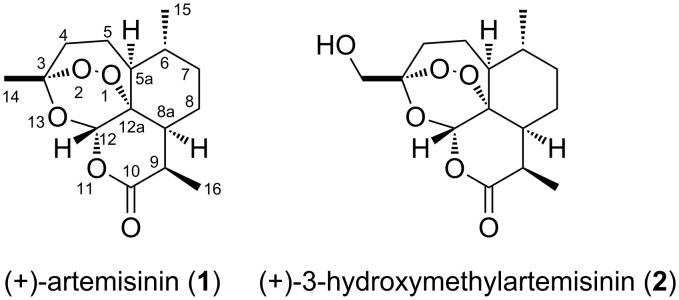
Structures of the natural (+)-artemisinin (**1**) and the synthesized (+)-3-hydroxymethylartemisinin (**2**).

The isolation of artemisinin (**1**; qinghaosu, Figure 1) from *Artemisia annua* L. and the discovery of its antimalarial properties in 1971 represent one of the greatest medical breakthroughs of the 20th century [1–2]. For these achievements Youyou Tu was awarded the Nobel Prize in Physiology or Medicine in 2015 [3]. As artemisinin has poor solubility and bioavailability, several derivatives of this natural product like artesunate and artemether were developed and are currently used in combination with other drugs for the treatment of malaria [4]. However, the exact mechanism of action of this endoperoxide sesquiterpene lactone is still unknown [5].

Whereas artemisinins are almost non-toxic to normal cells, several studies have confirmed their potent antitumor activity [6–7]. In addition, they have been reported to possess immunosuppressive, anti-inflammatory, antiviral, antifungal and antiparasitic activities [8–10]. Recently, it was shown that artemisinin interacts with the mammalian protein gephyrin and by stabilizing it, it enhances GABA_A_ receptor signaling resulting in in vivo conversion of pancreatic α-cells into functional β-like cells [11]. Therefore, this sesquiterpene lactone may also find an application in the treatment of diabetes mellitus. These facts indicate the clinical potential of artemisinins and “it is likely that artemisinin drugs will become a major armamentarium combating a variety of human diseases beyond malaria” [9].

In the past, several semi-synthetic artemisinin derivatives were prepared for experimental therapy of pathologies like the ones mentioned before [12–13]. However, the variety of these derivatives is limited as all of them are produced modifying the artemisinin skeleton at the same positions due to intrinsic synthetic challenges. Recently, we reported a biomimetic artemisinin synthesis that addresses these challenges and pave the way for derivatization of the (+)-artemisinin skeleton at positions not accessible using current methodology [14]. Based on our approach we report here on the synthesis and biological activity of the title compound (+)-3-hydroxymethylartemisinin (**2**, Figure 1).

## Results and Discussion

**Scheme 1:**
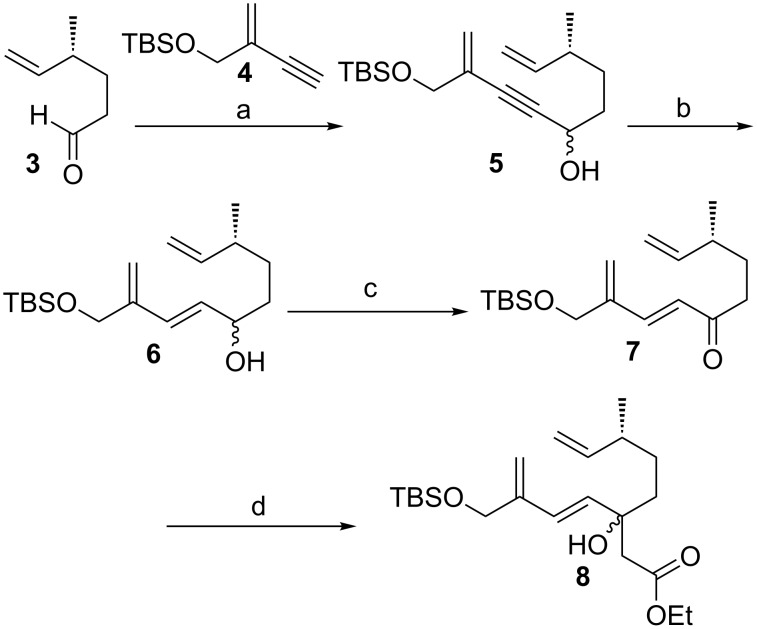
Synthesis of the Diels–Alder precursor **8** over four steps in 71% yield, starting from aldehyde **3** and alkyne **4**. Reaction conditions: a) alkyne **4**, *n*-BuLi, THF, −78 °C to rt, 16 h, 86%; b) Red-Al, THF, 0 °C, 10 min, 96%; c) BAIB, TEMPO, DCM, rt, 16 h, 92%; d) BrCH_2_CO_2_Et, Zn, toluene, reflux, 30 min, 93%. Red-Al = sodium bis(2-methoxyethoxy)aluminum dihydride, BAIB = (diacetoxyiodo)benzene, TEMPO = (2,2,6,6-tetramethylpiperidin-1-yl)oxyl.

**Scheme 2:**
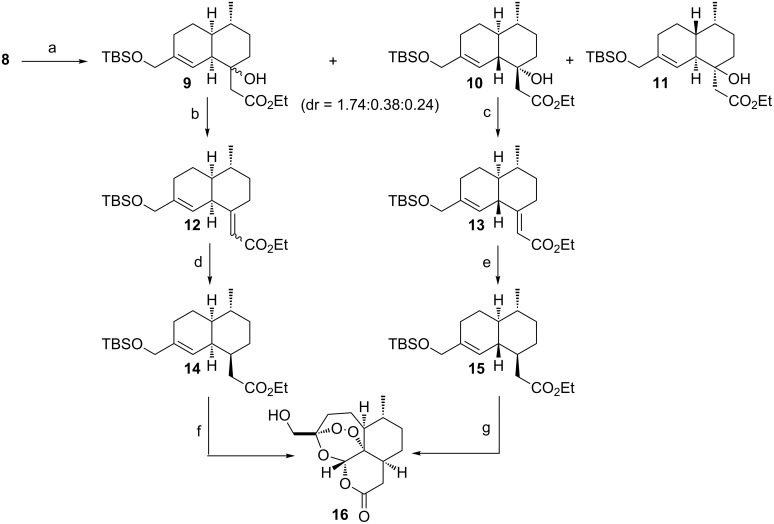
Synthesis of (+)-3-hydroxymethyl-9-desmethylartemisinin (**16**), starting from Diels–Alder derivatives **9** and **10**. Reaction conditions: a) toluene, 190 °C, 24 h, 84% (dr **9**:**10**:**11** = 1.74:0.38:0.24); b) Martin sulfurane, DCM, 0 °C, 10 min, 97% [(*E*)/(*Z*) = 1:1]; c) Martin sulfurane, DCM, 0 °C, 10 min, 95%; d) NiCl_2_, NaBH_4_, −60 °C to −40 °C, 1 h, 96% (dr = 1:0.03); e) Li, EtOH, NH_3_, −70 °C, 10 min, 61% (dr = 1:0.4); f) i. O_2_, methylene blue, light, DCM, −30 °C, 30 h; ii. then O_2_, cat. TFA, DCM, rt, 2 d, 24%; g) i. O_2_, methylene blue, light, DCM, −30 °C, 30 h; ii. then O_2_, cat. TFA, DCM, rt, 2 d, 16% [13].

Our synthesis (Scheme 1) started from the known and readily available aldehyde **3** [14] which was treated with the organolithium species obtained from derivative **4** [15] and *n*-BuLi to yield derivative **5**. The latter afforded allylic alcohol **6** after reduction of the propargylic moiety with Red-Al. BAIB/TEMPO oxidation of this alcohol gave ketone **7**. By a Reformatsky reaction of **7** using Zn/ethyl bromoacetate derivative **8** was obtained, which was subjected to a thermal (190 °C, toluene) intramolecular Diels–Alder reaction resulting in the formation of the β-hydroxy esters **9**, **10** and **11** (dr = 1.74:0:38:0.24) in a total yield of 84% (Scheme 2). Gratifyingly, the percentage of derivatives **9** and **10** that show the desired stereochemistry at carbon center 5a is around 90%.

The structure of all these isomers was confirmed by NOE experiments (see Supporting Information File 1 for full experimental details). Both derivatives **9** and **10** (Scheme 2) yielded α,β-unsaturated esters **12** and **13** after treatment with Martin sulfurane [16]. Reduction of compound **12** using NiCl_2_/NaBH_4_ in methanol as solvent yielded derivative **14** with excellent diastereomeric ratio (dr = 1:0.03). On the other hand, derivative **13** was reduced under Birch conditions (Li/NH_3_) to afford ester **15** in 61% yield (dr = 1:0.4). Gratifyingly, both artemisinin precursors **14** and **15** possess the desired stereoconfiguration at carbon center 8a.

**Scheme 3:**
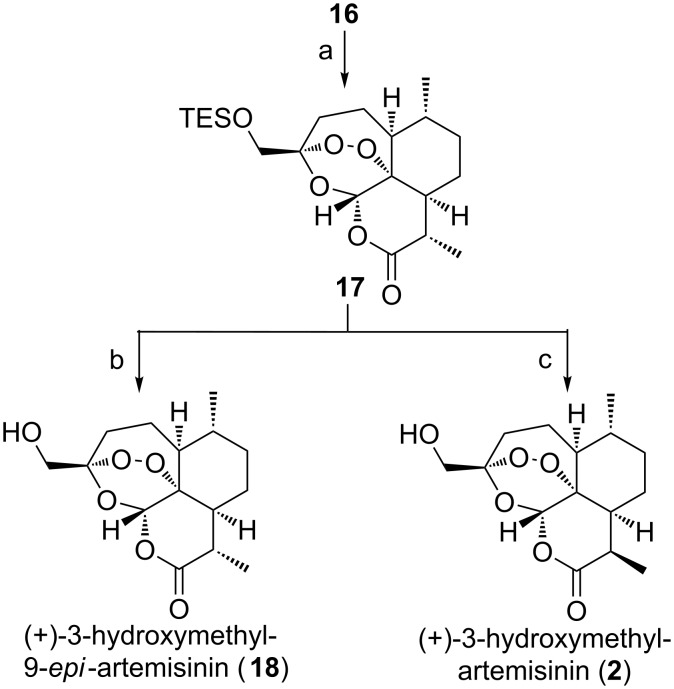
Synthesis of (+)-3-hydroxymethyl-9-*epi*-artemisinin (**18**) and (+)-3-hydroxymethylartemisinin (**2**). Reaction conditions: a) i. TESCl, NEt_3_, 0 °C, 16 h; ii. LDA, MeI, HMPA, THF, −78 °C to −50 °C, 1 h, 73% (over two steps); b) TBAF, THF, 0 °C, 10 min, 95%; c) i. DBU, DCM, rt, 1 d; ii. TBAF, THF, 0 °C, 10 min, 87% (over two steps). LDA = lithium diisopropylamide, HMPA = hexamethylphosphoramide, DBU = 1,8-diazabicyclo[5.4.0]undec-7-ene, TESCl = triethylsilyl chloride.

Subsequently, a solution of **14** and **15** in dichloromethane containing methylene blue as photosensitizer was exposed to sunlight and oxygen. The treatment of the resulting intermediate hydroperoxide with a small amount of trifluoroacetic acid as previously described [17–18], afforded in the frame of a Hock cleavage (+)-3-hydroxymethyl-9-desmethylartemisinin (**16**) in 24% and 16% yield, respectively. Protection of the free hydroxy group of **16** as a silyl ether and methylation of the obtained lactone in α-position (LDA/MeI/HMPA) afforded derivative **17**. After removal of the TES protecting group (+)-3-hydroxymethyl-9-*epi*-artemisinin (**18**, Scheme 3) was obtained, whereas the desired (+)-3-hydroxymethylartemisinin (**2**) was produced from **17** in two steps including epimerization and cleavage of the TES group in excellent yield (Scheme 3).

Finally, we evaluated the antimalarial activity of (+)-3-hydroxymethylartemisinin (**2**) as well as of the derivatives **16** and **18** against the drug-sensitive *P. falciparum* NF54 strain as described previously [14]. We found them to be inactive. In addition, **2** did not show any toxicity against L6 cells (a primary cell line derived from rat skeletal myoblasts). In both assays the highest concentration used was 100 μg/mL.

## Conclusion

In the past it has been postulated that artemisinins kill intraerythrocytic parasites such as *P. falciparum* in the presence of Fe^2+^ forming reactive oxygen species (ROS) [4]. As artemisinin and similar derivatives are destroyed by Fe^2+^ in vitro (Fenton reaction), it is difficult for us to explain their inactivity against *Plasmodium*. Furthermore, in cellular systems free ferrous iron [19] as well as haem [20–21] have been proposed to be the main iron sources for artemisinin activation. However, the results on that matter remain controversial [4,22]. Our results challenge the radical hypothesis of artemisinins action and also indicate that the methyl group at the C-3 position of artemisinin is possibly involved in lipophilic interactions with putative proteins essential for survival of the *Plasmodium* parasite. Derivative **2** will be used to attach further substituents at position 14 of artemisinin to prove this hypothesis.

## Experimental

Experimental details, NMR spectra and other physical data are shown in Supporting Information File 1.

## Supporting Information

File 1 Experimental part.
