# Supplementary material for: Synthesis and biological investigation of (+)-3-hydroxymethylartemisinin
Source: Beilstein J Org Chem. 2019 Feb 27;15:567–70. doi: 10.3762/bjoc.15.51 (PMC6404461; doi:10.3762/bjoc.15.51)
Supplement: File 1 — Experimental part. [file Beilstein_J_Org_Chem-15-567-s001.pdf]

BEILSTEIN JOURNAL OF ORGANIC CHEMISTRY

Supporting Information

for

Synthesis and biological investigation of (+)-3-hydroxymethylartemisinin

Toni Smeilus, Farnoush Mousavizadeh, Johannes Krieger, Xingzhao Tu, Marcel Kaiser and Athanassios Giannis

Beilstein J. Org. Chem. **2019,** *15,* 567–570. doi:10.3762/bjoc.15.51

Experimental part

License and Terms: This is a supporting information file under the terms of the Creative Commons Attribution License (http://creativecommons.org/licenses/by/4.0). Please note that the reuse, redistribution and reproduction in particular requires that the authors and source are credited.
The license is subject to the *Beilstein Journal of Organic Chemistry* terms and conditions: (https://www.beilstein-journals.org/bjoc)

General procedures

All reactions were run under an atmosphere of argon unless otherwise indicated. Room temperature refers to 22 °C. Reagents and anhydrous solvents were transferred via oven-dried syringe or cannula. Flasks were flame-dried under vacuum and cooled under a constant stream of argon.

THF was dried over potassium. Et_2O and toluene were dried over sodium. DCM was dried over SICAPENT®.

All other chemicals were purchased from ABCR, Acros, Alfa Aesar, Fluorochem, Merck, Sigma-Aldrich, and TCI Europe at highest commercially available purity and used as such.

Reactions were monitored by thin-layer chromatography using Macherey-Nagel silica gel (Alugram XtraSil G/UV_{254}, 0.2 mm coating thickness) TLC aluminium sheets and visualized with ceric ammonium molybdate, potassium permanganate or vanillin staining solution. Chromatographic purification was performed as flash chromatography on Macherey-Nagel silica gel 60 using a forced flow of eluent (method of Still). Yields refer to chromatographically purified and spectroscopically pure compounds.

NMR spectra were recorded on a Varian Mercury plus 400 (operating at 400 MHz for ^{1}H and 100.64 MHz for ^{13}C acquisitions), and a Varian Mercury plus 300 (operating at 300 MHz for ^{1}H and 75 MHz for ^{13}C acquisitions). Chemical shifts are reported in ppm with the solvent resonance as the internal standard [d_1-chloroform: 7.26 (^{1}H NMR), 77.16 (^{13}C NMR); d_6-dimethyl sulfoxide: 2.50 (^{1}H NMR), 39.52 (^{13}C NMR)]. Coupling constants J are given in Hertz (Hz). Multiplicities are classified by the following abbreviations: s = singlet, d = doublet, t = triplet, q = quartet, and combinations thereof, or m = multiplet, or br = broad signal. Where 2D-spectra were recorded and allowed complete assignment of all hydrogen- and carbon-atoms of a compound, spectral data include this assignment using common numbering. All spectra can be found as copies at the end of the experimental section.

High resolution mass spectra were obtained on a Bruker Daltonics ESI-FT-ICR-MS APEX II. IR spectra were obtained on an ATI/MATTSON Genesis FTIR as thin film (in CCl_4) or KBr disk. Absorbance frequencies are reported in reciprocal centimetres (cm^{-1}). Melting points were measured on a Boetius-micro hot stage and are uncorrected. Optical rotation data was obtained with a Schmidt+Haensch Polartronic MHz-8 at the sodium-D line (589.44 nm) using a 50 mm path-length cell in the solvent and concentration indicated.

Reactionscheme:

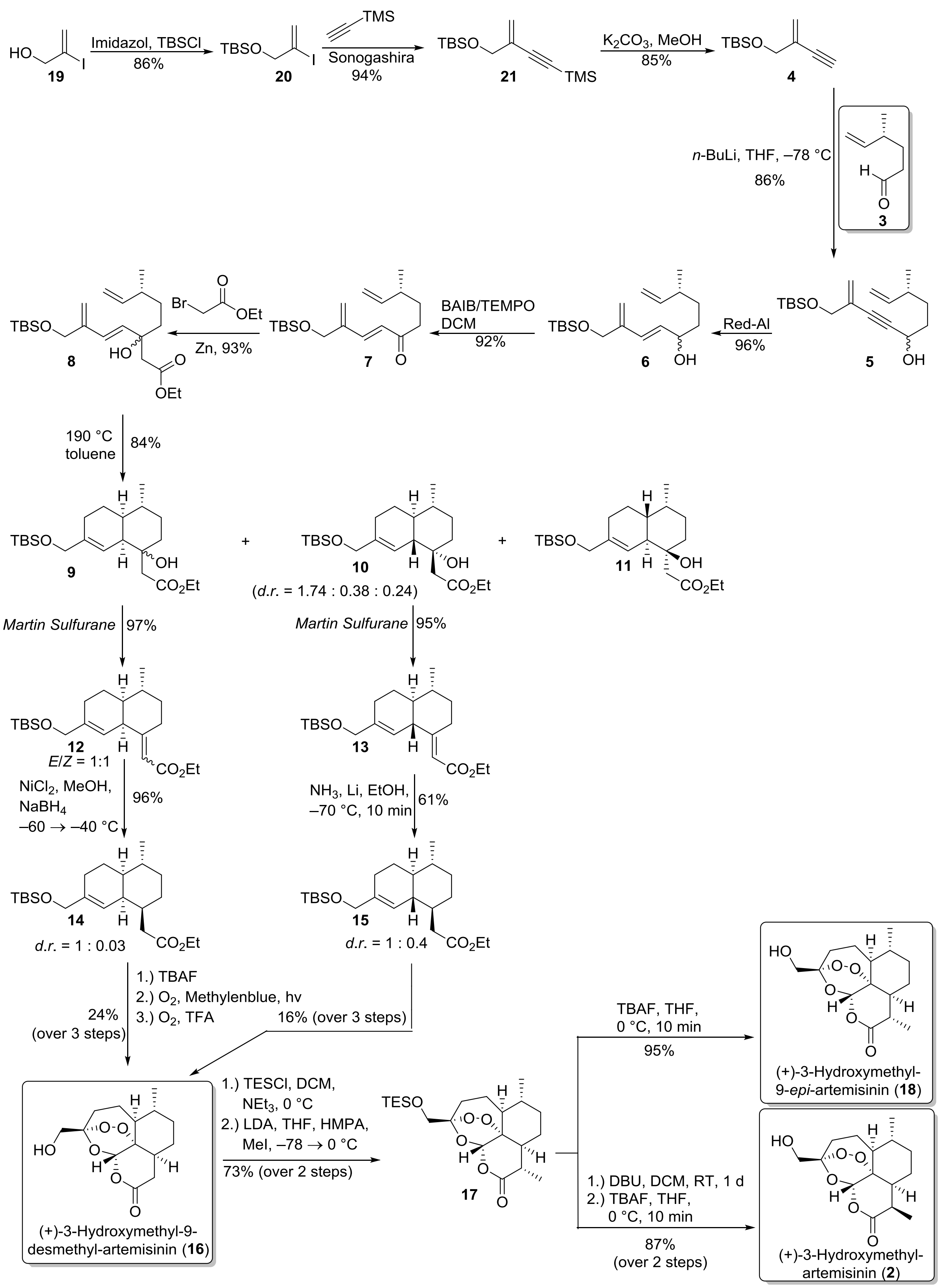

tert-Butyl((2-iodoallyl)oxy)dimethylsilane (20)[1]

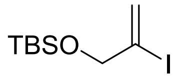

To a stirred solution of the alcohol **19** (14.7 g, 79.9 mmol) in DCM (100 mL) TBSCl (13.2 g, 87.9 mmol) was added and the resulting solution was cooled to 0 °C. Imidazole (5.98 g, 87.9 mmol) was added in portions and the mixture was warmed to room temperature and stirred for further 2 h. Water was added to the yellow suspension and the phases were separated. The aqueous phase was extracted with DCM (2 × 100 mL) and the combined organic phases were washed with water (200 mL) and saturated NaCl-solution (200 mL), dried over Na_2SO_4 and filtered. The solvent was removed under reduced pressure and the crude product was purified by silica gel chromatography (*n*-hexane/EtOAc, 15:1 v/v) to yield the TBS-protected alcohol **20** (20.4 g, 68.4 mmol, 86%) as a violet oil. TLC (*n*-hexane/EtOAc, 3:1 v/v): R_f = 0.84; IR (film): $\tilde{\nu}_{max}$ 2954, 2929, 2885, 2856; 1626, 1471, 1256, 1134, 1082, 839, 778 cm^{-1}; ^1H-NMR ($CDCl_3$, 400 MHz): δ 6.44-6.40 (m, 1H), 5.83-5.79 (m, 1H), 4.19-4.15 (m, 2H), 0.92 (s, 9H), 0.09 (s, 6H); ^{13}C-NMR ($CDCl_3$, 100 MHz): δ 123.1, 109.9, 71.2, 26.0, 18.5, −5.2; HRMS (*m/z*): $[M+Ag]^+$ calc. for $C_9H_{19}IOSiAg$: 404.93009, found: 404.92952.

tert-Butyldimethyl((2-methylene-4-(trimethylsilyl)but-3-yn-1-yl)oxy)silane (21)[2]

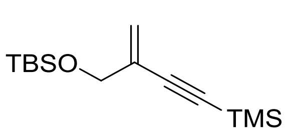

To a stirred solution of allyl iodide **20** (20.4 g, 68.4 mmol) in THF (120 mL) CuI (1.30 g, 6.84 mmol), NEt_3 (20.8 g, 28.4 mL, 0.205 mol), TMS-acetylene (10.1 g, 14.6 mL, 0.103 mol) and $Pd(PPh_3)_4$ (1.98 g, 1.71 mmol) were added at 0 °C. The resulting mixture was warmed to room temperature and stirred for 3 h. Afterwards suspension was filtered through a short plug of celite. MTBE (200 mL) and water (200 mL) were

[1] Ganic, A.; Pfaltz, A. *Chem. Eur. J.* **2012**, *18*, 6724-6728.
[2] Charpenay, M.; Boudhar, A., Hulot, C.; Blond G.; Suffert, J. *Tetrahedron* **2013**, *69*, 7568-7591.

added and the phases were separated. The aqueous phase was extracted with MTBE (2 × 100 mL). The combined organic phases were washed with water (200 mL) and saturated NaCl solution (200 mL), dried over Na_2SO_4 and filtered. The solvent was removed under reduced pressure and the crude product was purified by silica gel chromatography (*n*-hexane/EtOAc, 50:1 v/v) to yield **21** (17.3 g, 64.3 mmol, 94%) as yellow liquid. TLC (*n*-hexane): $R_f = 0.37$; IR (film): $\tilde{\nu}_{max}$ 2958, 2930, 2857, 2151, 1618, 1472, 1252, 1116, 842 cm^{-1}; ^1H-NMR ($CDCl_3$, 400 MHz): δ 5.61-5.57 (m, 1H), 5.51-5.46 (m, 1H), 4.16-4.11 (m, 2H), 0.92 (s, 9H), 0.19 (s, 9H), 0.09 (s, 6H); ^{13}C-NMR ($CDCl_3$, 100 MHz): δ 131.2, 120.6, 103.3, 95.4, 65.2, 26.0, 18.5, 0.1, −5.2; HRMS (*m/z*): $[M+H]^+$ calc. for $C_{14}H_{29}OSi_2$: 269.17514, found: 269.17498; $[M+Na]^+$ calc. for $C_{14}H_{28}OSi_2Na$: 291.15709, found: 291.15700.

***tert*-Butyldimethyl((2-methylenebut-3-yn-1-yl)oxy)silane (4)[2]**

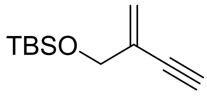

To a solution of **21** (16.6 g, 61.8 mmol) in MeOH (150 mL) K_2CO_3 (1.71 g, 12.4 mmol) was added and the resulting mixture was stirred for 1 h at room temperature. Then, water (200 mL) was added and the solution was extracted with MTBE (2 × 200 mL). The combined organic phases were washed with water (200 mL) and saturated NaCl solution (200 mL), dried over Na_2SO_4 and filtered. The solvent was removed under reduced pressure and the crude product was purified by silica gel chromatography (*n*-hexane/EtOAc, 50:1 v/v) to yield **4** (10.4 g, 53.0 mmol, 85%) as colorless liquid. TLC (*n*-hexane): $R_f = 0.48$; IR (film): $\tilde{\nu}_{max}$ 2956, 2930, 2858, 1620, 1472, 1257, 1115, 838 cm^{-1}; ^1H-NMR ($CDCl_3$, 300 MHz): δ 5.68-5.64 (m, 1H), 5.57-5.53 (m, 1H), 4.18-4.14 (m, 2H), 2.91 (s, 1H), 0.92 (s, 9H), 0.09 (s, 6H); ^{13}C-NMR ($CDCl_3$, 100 MHz): δ 130.2, 121.2, 82.0, 78.3, 65.0, 26.0, 18.5, −5.3; HRMS (*m/z*): $[M+Ag]^+$ calc. for $C_{11}H_{20}OSiAg$: 303.03344, found: 303.03300.

(8*R*)-2-(((*tert*-Butyldimethylsilyl)oxy)methyl)-8-methyldeca-1,9-dien-3-yn-5-ol (5)

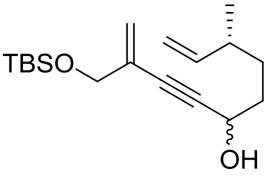

m-CPBA (11.8 g, 52.4 mmol, 77% purity) was added in small portions to a suspension of (*R*)-(−)-citronellene (6.04 g, 43.7 mmol) and NaOAc (4.66 g, 56.8 mmol) in DCM (200 mL) at 0 °C. Stirring at this temperature was continued for 1 h. The reaction was finished by addition of saturated $NaHCO_3$ solution (200 mL) and the phases were separated. The organic phase was washed with 1 M NaOH solution (3 × 200 mL), was dried over Na_2SO_4 and filtered. The solvent was removed under reduced pressure and the crude epoxide was used without further purification in the next step.

The crude epoxide was dissolved in Et_2O (200 mL) and a solution of H_5IO_6 (14.9 g, 65.5 mmol) in THF (200 mL) was added at 0 °C. Stirring at this temperature was continued for 1 h, then water (250 mL) and Et_2O (300 mL) was added. The phases were separated and the aqueous phase was extracted with Et_2O (2 × 100 mL). The combined organic phases were washed with water (300 mL) and saturated NaCl solution (200 mL), dried over Na_2SO_4 and filtered, most of the solvent was removed under reduced pressure (40 °C, 180 mbar) and the corresponding aldehyde was used without further purification in the next step.

To a stirred solution of **4** (10.3 g, 52.4 mmol) in THF (150 mL) *n*-BuLi (21.0 mL, 52.4 mmol, 2.5 M in hexane) was added slowly at −78 °C. The solution was warmed to 0 °C within 1 h and then cooled again to −78 °C. The previous prepared aldehyde was solved in THF (100 mL) and slowly added to the reaction mixture, which was afterwards warmed to room temperature overnight. Water (200 mL) and MTBE (400 mL) was added, the phases were separated and the aqueous phase was extracted with MTBE (2 × 200 mL). The combined organic phases were washed with water (300 mL) and saturated NaCl solution (300 mL), dried over Na_2SO_4 and filtered. The solvent was removed under reduced pressure and the crude product was purified by silica gel chromatography (*n*-hexane/EtOAc, 10:1 v/v) to yield **5** (11.6 g, 37.6 mmol, 86% over three steps, 1:1 mixture of epimers) as colorless oil. TLC (*n*-hexane/EtOAc, 10:1 v/v): R_f = 0.30; IR (film): $\tilde{\nu}_{max}$ 3365, 2955, 2929, 2858,

1620, 1471, 1463, 1256, 1119, 909, 838, 778 cm^{-1}; ^{1}H-NMR ($CDCl_3$, 300 MHz): δ 5.75-5.67 (m, 1H), 5.60-5.57 (m, 1H), 5.46-5.42 (m, 1H), 5.03-4.91 (m, 2H), 4.50-4.44 (m, 1H), 4.13-4.11 (m, 2H), 2.19-2.11 (m, 1H), 1.77-1.68 (m, 3H), 1.51-1.42 (m, 1H), 1.02 (d, J = 6.8 Hz, 3H), 0.92 (s, 9H), 0.09 (s, 6H); ^{13}C-NMR ($CDCl_3$, 75 MHz): δ 144.3, 130.5, 119.9, 113.2, 91.2, 83.1, 65.2, 63.1, 63.0, 37.7, 35.7, 32.1, 26.0, 20.5, 18.5, −5.2; HRMS (*m/z*): $[M+Na]^+$ calc. for $C_{18}H_{32}O_2SiNa$: 331.20693, found: 331.20668.

(8*R*,*E*)-2-(((*tert*-Butyldimethylsilyl)oxy)methyl)-8-methyldeca-1,3,9-trien-5-ol (6)

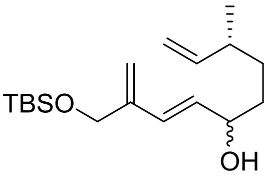

To a solution of **5** (10.5 g, 34.0 mmol) in THF (150 mL) Red-Al® (12.6 g, 37.4 mmol, 60 wt %, 3.6 mol/L in toluene) was added slowly at 0 °C. This solution was stirred for 10 min before water (50 mL) was added carefully. The solution was extracted with MTBE (3 × 50 mL) and the combined organic phases were washed with water (100 mL) and saturated NaCl solution (100 mL), dried over Na_2SO_4 and filtered. The solvent was removed under reduced pressure and the crude product was purified by silica gel chromatography (*n*-hexane/EtOAc, 10:1 v/v) to yield **6** (10.1 g, 32.5 mmol, 96%) as colorless oil. TLC (*n*-hexane/EtOAc, 10:1 v/v): R_f = 0.28; IR (film): $\tilde{\nu}_{max}$ 3409, 2955, 2884, 2858, 1710, 1639, 1471, 1463, 1255, 1112 cm^{-1}; ^{1}H-NMR ($CDCl_3$, 300 MHz): δ 6.22 (d, J = 16.2 Hz, 1H), 5.73-5.61 (2H, m), 5.30-5.28 (1H, m), 5.12-5.07 (m, 1H), 4.99 – 4.87 (m, 2H), 4.30 (s, 2H), 4.10 (q, J = 6.6 Hz, 1H), 2.21 – 2.01 (m, 1H), 1.63 – 1.49 (m, 2H), 1.43 – 1.24 (m, 2H), 0.99 (d, J = 6.7 Hz, 3H), 0.92 (s, 9H), 0.08 (s, 6H).; ^{13}C-NMR ($CDCl_3$, 75 MHz): δ 144.5, 144.2, 131.8, 130.1, 115.0, 113.0, 73.4, 63.0, 38.0, 35.2, 32.42, 26.0, 20.4, 18.5, −5.2; HRMS (*m/z*): $[M+Na]^+$ calc. for $C_{18}H_{34}O_2SiNa$: 333.22203, found: 333.22191.

(*R*,*E*)-2-(((*tert*-Butyldimethylsilyl)oxy)methyl)-8-methyldeca-1,3,9-trien-5-one (7)

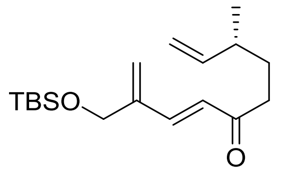

To a stirred solution of the allylic alcohol **6** (8.04 g, 25.9 mmol) in DCM (160 mL) BAIB (9.17 g, 28.5 mmol) and TEMPO (0.40 g, 2.59 mmol) were added. The solution was stirred overnight and afterwards the reaction was finished by the addition of saturated $Na_2S_2O_3$ solution (100 mL). The phases were separated and the aqueous phase was extracted with DCM (3 × 50 mL). The combined organic phases were washed with water (100 mL) and saturated NaCl solution (100 mL), dried over Na_2SO_4 and filtered. The solvent was removed under reduced pressure and the crude product was purified by silica gel chromatography (*n*-hexane/EtOAc, 50:1 > 20:1 v/v) to yield **7** (7.37 g, 23.9 mmol, 92%) as colorless oil. TLC (*n*-hexane/EtOAc, 10:1 v/v): R_f = 0.56; $[\alpha]_D^{22}$ (deg cm^3 g^{-1} dm^{-1}) = −13.6° (c = 0.0041 in $CHCl_3$); IR (film): $\tilde{\nu}_{max}$ 2956, 2929, 2857, 1693, 1667, 1597, 1463, 1258, 1119 cm^{-1}; ^{1}H-NMR ($CDCl_3$, 300 MHz): *δ* 7.17 (d, *J* = 16.4 Hz, 1H), 6.15 (d, *J* = 16.4 Hz, 1H), 5.72 – 5.61 (m, 2H), 5.51 (s, 1H), 5.02 – 4.94 (m, 2H), 4.33 (d, *J* = 1.7 Hz, 2H), 2.59 – 2.52 (m, 2H), 2.18 – 2.10 (m, 1H), 1.74 – 1.50 (m, 3H), 1.02 (d, *J* = 6.7 Hz, 3H), 0.92 (s, 9H), 0.09 (s, 6H); ^{13}C-NMR ($CDCl_3$, 75 MHz): *δ* 200.9, 143.9, 143.8, 141.6, 126.0, 122.9, 113.6, 62.4, 38.5, 37.7, 30.7, 26.0, 20.5, 18.5, −5.2; HRMS (*m/z*): $[M+H]^+$ calc. for $C_{18}H_{33}O_2Si$: 309.22443, found: 309.22420, $[M+Na]^+$ calc. for $C_{18}H_{32}O_2SiNa$: 331.20638, found: 331.20592, $[2M+Na]^+$ calc. for $C_{36}H_{64}O_4Si_2Na$: 639.42353, found: 639.42351.

Ethyl (6*R*)-3-((*E*)-3-(((*tert*-butyldimethylsilyl)oxy)methyl)buta-1,3-dien-1-yl)-3-hydroxy-6-methyloct-7-enoate (8)

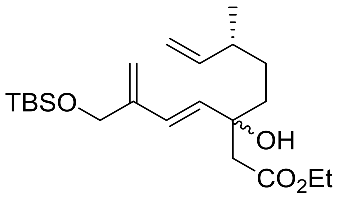

A suspension of activated zinc dust[3] (7.74 g, 118 mmol), iodine (60.1 mg, 0.24 mmol) and toluene (110 mL) was stirred under reflux for 5 min and cooled to room temperature. To this mixture ethyl bromoacetate (7.91 g, 5.24 mL, 47.4 mmol) was added first. Afterwards, ketone **7** (7.31 g, 23.7 mmol) solved in toluene (20 mL) was added to the suspension. The resulting mixture was stirred under reflux for 30 min. The reaction was cooled to 0 °C and water (50 mL) was added. The suspension was filtered and the filtrate was extracted with MTBE (3 × 100 mL). The combined organic phases were washed with water (150 mL) and saturated NaCl solution (150 mL), dried over Na_2SO_4, filtered and concentrated under reduced pressure. The crude product was purified by silica gel chromatography (*n*-hexane/EtOAc, 15:1 v/v) to yield **8** (8.74 g, 22.0 mmol, 93%) as colorless oil. TLC (*n*-hexane/EtOAc, 15:1 v/v): R_f = 0.39; IR (film): $\tilde{\nu}_{max}$ 3409, 2955, 2928, 2856, 1716, 1644, 1384, 1096, 1070, 1026 cm^{-1}; ^{1}H-NMR ($CDCl_3$, 300 MHz): δ 6.28 (d, J = 16.2 Hz, 1H), 5.72 – 5.57 (m, 2H), 5.31 – 5.23 (m, 1H), 5.13 – 5.06 (m, 1H), 4.98 – 4.88 (m, 2H), 4.31 – 4.23 (m, 2H), 4.21 – 4.06 (m, 2H), 2.55 (s, 2H), 2.05 (m, 1H), 1.69 – 1.26 (m, 3H), 1.23 (t, J = 7.1 Hz, 3H), 0.98 (d, J = 6.8 Hz, 3H), 0.92 (s, 9H), 0.08 (s, 6H); ^{13}C-NMR ($CDCl_3$, 75 MHz): δ 172.7, 144.5, 144.0, 132.8, 128.6, 114.9, 113.0, 73.4, 63.2, 60.9, 44.5, 39.2, 38.2, 30.2, 26.0, 20.4, 18.5, 14.3, −5.2; HRMS (*m/z*): $[M+Na]^+$ calc. for $C_{22}H_{40}O_4SiNa$: 419.25881, found: 419.25855, $[2M+Na]^+$ calc. for $C_{44}H_{80}O_8Si_2Na$: 815.52839, found: 815.52936.

Intramolecular *Diels–Alder*-reaction

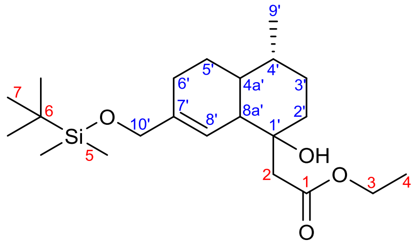

A solution of triene **8** (1.01 g, 2.55 mmol) in toluene (degassed) was heated in a pressure flask to 190 °C for 24 h. Afterwards the solvent was removed under reduced pressure and the crude product was purified by silica gel chromatography (*n*-

[3] Weiss, D.; Hope, S.; Beckert, R., Klemm, D. *Journal f. prakt. Chemie* **1990**, *332*, 367-374.

hexane/EtOAc, 20:1 v/v) to yield the *Diels-Alder*-products **9/10/11** (0.85 g, 2.14 mmol, 84%, 1.74 : 0.38 : 0.24) as colorless oil.

Ethyl 2-((1*R*,4*R*,4a*S*,8a*R*)-7-(((*tert*-butyldimethylsilyl)oxy)methyl)-1-hydroxy-4-methyl-1,2,3,4,4a,5,6,8a-octahydronaphthalen-1-yl)acetate (9a)

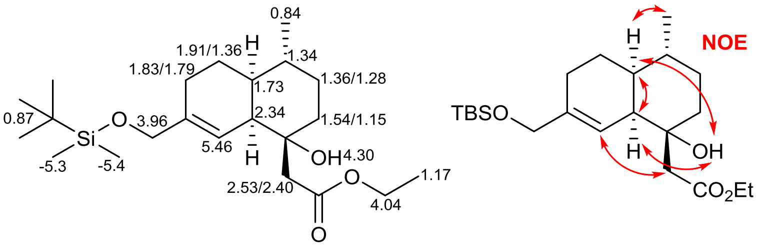

TLC (*n*-hexane/EtOAc, 15:1 v/v): $R_f = 0.35$; $[\alpha]_D^{22}$ (deg cm^3 g^{-1} dm^{-1}) = +10.2° (c = 0.0055 in $CHCl_3$); IR (film): $\tilde{\nu}_{max}$ 3500, 2952, 2927, 2857, 1715, 1463, 1372, 1253, 1186, 837 cm^{-1}; ^{1}H-NMR ($CDCl_3$, 300 MHz): δ 5.46 (s, 1H), 4.31 (s, 1H), 4.04 (q, J = 7.1 Hz, 2H), 3.96 (s, 2H), 2.53 (d, J = 13.9 Hz, 1H), 2.41 (d, J = 13.9 Hz, 1H), 2.36 – 2.31 (m, 1H), 1.96 – 1.86 (m, 1H), 1.86 – 1.70 (m, 3H), 1.58 – 1.47 (m, 1H), 1.42 – 1.23 (m, 4H), 1.17 (t, J = 7.1 Hz, 3H), 0.87 (s, 9H), 0.84 (d, J = 5.4 Hz, 3H), 0.03 (s, 6H); ^{13}C-NMR ($CDCl_3$, 75 MHz): δ 170.6 (C1), 137.5 (C7'), 120.8 (C8'), 72.0 (C1'), 66.4 (C10'), 59.4 (C3), 44.9 (C8a'), 44.9 (C2), 35.7 (C4a'), 33.7 (C2'), 29.2 (C3'), 26.8 (C4'), 25.7 (C7), 24.4 (C5'), 20.9 (C6'), 19.5 (C9'), 17.9 (C6), 14.1 (C4), −5.3 (C5), −5.4 (C5); HRMS (*m/z*): [M+Na]$^+$ calc. for $C_{22}H_{40}O_4SiNa$: 419.25881, found: 419.25892, [2M+Na]$^+$ calc. for $C_{44}H_{80}O_8Si_2Na$: 815.52839, found: 815.52839.

Ethyl 2-((1*S*,4*R*,4a*S*,8a*R*)-7-(((*tert*-butyldimethylsilyl)oxy)methyl)-1-hydroxy-4-methyl-1,2,3,4,4a,5,6,8a-octahydronaphthalen-1-yl)acetate (9b)

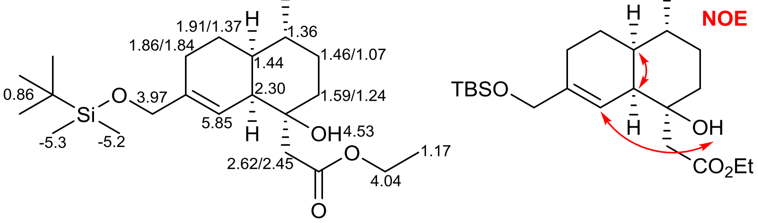

TLC (*n*-hexane/EtOAc, 15:1 v/v): $R_f = 0.34$; $[\alpha]_D^{22}$ (deg cm^3 g^{-1} dm^{-1}) = −2.1° (c = 0.0115 in $CHCl_3$); IR (film): $\tilde{\nu}_{max}$ 3511, 2952, 2928, 2857, 1715, 1463, 1254, 1184,

1063, 837 cm^{-1}; ^{1}H-NMR ($CDCl_3$, 300 MHz): δ 5.87 – 5.84 (m, 1H), 4.53 (s, 1H), 4.04 (q, J = 7.1 Hz, 2H), 4.02 – 3.92 (m, 2H), 2.62 (d, J = 13.5 Hz, 1H), 2.44 (d, J = 13.5 Hz, 1H), 2.34 – 2.25 (m, 1H), 1.96 – 1.80 (m, 3H), 1.63 – 1.55 (m, 1H), 1.51 – 1.42 (m, 2H), 1.43 – 1.29 (m, 2H), 1.28 – 1.22 (m, 1H), 1.17 (t, J = 7.1 Hz, 3H), 1.12 – 1.02 (m, 1H), 0.86 (s, 9H), 0.85 (d, J = 5.4 Hz, 3H), 0.03 (s, 6H); ^{13}C-NMR ($CDCl_3$, 75 MHz): δ 171.1 (C1), 136.4 (C7‘), 121.6 (C8‘), 72.4 (C1‘), 67.2 (C10‘), 59.4 (C3), 45.0 (C8a‘), 43.1 (C2), 37.8 (C4a‘), 33.3 (C2‘), 31.0 (C3‘), 27.0 (C4‘), 25.8 (C7), 24.7 (C5‘), 21.1 (C6‘), 19.1 (C9‘), 18.0 (C6), 14.2 (C4), −5.2 (C5), −5.3 (C5); HRMS (m/z): $[M+Na]^+$ calc. for $C_{22}H_{40}O_4SiNa$: 419.25881, found: 419.25875.

Ethyl 2-((1*R*,4*R*,4a*S*,8a*S*)-7-(((*tert*-butyldimethylsilyl)oxy)methyl)-1-hydroxy-4-methyl-1,2,3,4,4a,5,6,8a-octahydronaphthalen-1-yl)acetate (10)

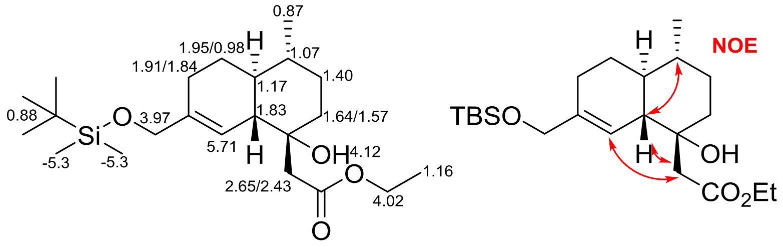

TLC (n-hexane/EtOAc, 15:1 v/v): $R_f = 0.31$; $[\alpha]_D^{22}$ (deg cm^3 g^{-1} dm^{-1}) = +13.4° (c = 0.0027 in $CHCl_3$); IR (film): $\tilde{\nu}_{max}$ 3503, 2952, 2927, 2856, 1732, 1715, 1463, 1372, 1254, 1189, 1071, 838 cm^{-1}; ^{1}H-NMR ($CDCl_3$, 300 MHz): δ 5.74 – 5.68 (m, 1H), 4.12 (s, 1H), 4.02 (q, J = 7.1 Hz, 2H), 4.01 – 3.91 (m, 2H), 2.64 (d, J = 13.8 Hz, 1H), 2.43 (d, J = 13.8 Hz, 1H), 2.03 – 1.78 (m, 4H), 1.72 – 1.62 (m, 1H), 1.61 – 1.52 (m, 1H), 1.43 – 1.34 (m, 2H), 1.27 – 1.21 (m, 1H), 1.16 (t, J = 7.1 Hz, 3H), 1.10 – 0.93 (m, 2H), 0.88 (s, 9H), 0.87 (d, J = 5.4 Hz, 3H), 0.04 (s, 6H); ^{13}C-NMR ($CDCl_3$, 75 MHz): δ 170.6 (C1), 137.1 (C7‘), 121.6 (C8‘), 71.0 (C1‘), 66.7 (C10‘), 59.6 (C3), 44.9 (C2), 36.8 (C2‘), 30.2 (C3‘), 26.0 (C5‘), 25.8 (C7), 25.3 (C6‘), 19.6 (C9‘), 18.0 (C6), 14.1 (C4), −5.3 (C5), −5.3 (C5); HRMS (m/z): $[M+Na]^+$ calc. for $C_{22}H_{40}O_4SiNa$: 419.25881, found: 419.25861, $[2M+Na]^+$ calc. for $C_{44}H_{80}O_8Si_2Na$: 815.52839, found: 815.53210.

Ethyl 2-((1*S*,4*R*,4a*R*,8a*R*)-7-(((*tert*-butyldimethylsilyl)oxy)methyl)-1-hydroxy-4-methyl-1,2,3,4,4a,5,6,8a-octahydronaphthalen-1-yl)acetate (11)

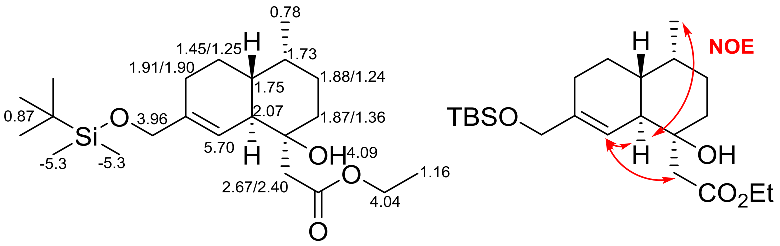

TLC (*n*-hexane/EtOAc, 15:1 v/v): $R_f = 0.28$; $[\alpha]_D^{22}$ (deg cm^3 g^{-1} dm^{-1}) = +6.1° (c = 0.0084 in $CHCl_3$); IR (film): $\tilde{\nu}_{max}$ 3513, 2955, 2927, 2856, 1731, 1713, 1463, 1370, 1255, 1181, 1061, 837 cm^{-1}; ^{1}H-NMR ($CDCl_3$, 300 MHz): δ 5.74 – 5.69 (m, 1H), 4.10 (s, 1H), 4.04 (q, *J* = 7.1 Hz, 2H), 4.01 – 3.91 (m, 2H), 2.67 (d, *J* = 13.9 Hz, 1H), 2.40 (d, *J* = 13.9 Hz, 1H), 2.12 – 2.03 (m, 1H), 1.95 – 1.85 (m, 3H), 1.81 – 1.70 (m, 2H), 1.52 – 1.43 (m, 1H), 1.40 – 1.32 (m, 1H), 1.31 – 1.22 (m, 1H), 1.16 (t, *J* = 7.1 Hz, 3H), 0.87 (s, 9H), 0.78 (d, *J* = 6.9 Hz, 3H), 0.04 (s, 3H), 0.03 (s, 3H); ^{13}C-NMR ($CDCl_3$, 75 MHz): *δ* 170.7 (C1), 137.3 (C7‘), 121.9 (C8‘), 71.2 (C1‘), 66.7 (C10‘), 59.6 (C3), 44.9 (C2), 39.6 (C8a‘), 36.1 (C4a‘), 31.8 (C4‘), 31.6 (C2‘), 27.8 (C3‘), 27.4 (C5‘), 25.8 (C7), 25.6 (C6‘), 18.0 (C6), 14.1 (C4), 11.7 (C9‘),−5.3 (C5), −5.3 (C5); HRMS (*m/z*): $[M+Na]^+$ calc. for $C_{22}H_{40}O_4SiNa$: 419.25881, found: 419.25814, $[2M+Na]^+$ calc. for $C_{44}H_{80}O_8Si_2Na$: 815.52839, found: 815.52641.

Ethyl 2-((4*R*,4a*S*,8a*R*)-7-(((*tert*-butyldimethylsilyl)oxy)methyl)-4-methyl-3,4,4a,5,6,8a-hexahydronaphthalen-1(2*H*)-ylidene)acetate (12)

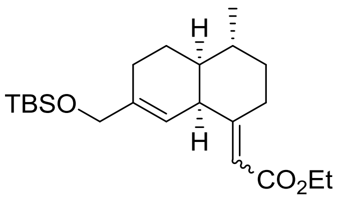

To a stirred solution of the tertiary alcohols **9a/b** (1.25 g, 3.15 mmol) in DCM (50 mL) *Martin Sulfurane* (2.54 g, 3.78 mmol) was added at 0 °C and the solution was stirred for 10 min. The reaction was finished by the addition of saturated $NaHCO_3$ solution (20 mL) and water (30 mL). The phases were separated and the aqueous phase was

extracted with DCM (2 × 40 mL). The combined organic phases were washed with saturated NaCl solution (100 mL), dried over Na_2SO_4 and filtered. The solvent was removed under reduced pressure and the crude product was purified by silica gel chromatography (*n*-hexane/EtOAc, 30:1 v/v) to yield **12** (1.16 g, 3.06 mmol, 97%, mixture of isomers *E*/*Z* = 1:1) as colorless oil. TLC (*n*-hexane/EtOAc, 10:1 v/v): $R_f = 0.61$; IR (film): $\tilde{\nu}_{max}$ 2953, 2928, 2856, 1715, 1644, 1463, 1380, 1254, 1165, 1147, 1065, 837 cm^{-1}; ^{1}H-NMR ($CDCl_3$, 400 MHz): δ 5.67 – 5.62 (m, 1H), 5.37 – 5.34 (m, 1H), 5.27 – 5.22 (m, 1H), 4.56 (s, 1H), 4.18 – 4.10 (m, 2H), 4.05 – 4.00 (m, 2H), 3.37 – 3.31 (m, 1H), 2.98 – 2.93 (m, 1H), 2.31 – 2.16 (m, 1H), 2.12 – 1.16 (m, 11H), 1.16 – 1.03 (m, 2H), 0.95 (d, *J* = 6.3 Hz, 3H), 0.90 (s, 9H), 0.89 (s, 9H), 0.06 (s, 6H), 0.05 (s, 6H); ^{13}C-NMR ($CDCl_3$, 75 MHz): δ 166.5, 166.3, 138.9, 137.8, 123.5, 122.6, 114.4, 113.8, 67.5, 67.2, 59.7, 49.9, 48.2, 46.1, 42.9, 41.7, 39.1, 36.2, 34.8, 34.2, 30.0, 28.4, 26.9, 24.7, 24.6, 22.5, 21.0, 19.6, 19.6, 14.5, −5.0, −5.1; HRMS (*m/z*): $[M+Na]^+$ calc. for $C_{22}H_{38}O_3SiNa$: 401.24824, found: 401.24954.

Ethyl 2-((4*R*,4a*S*,8a*S*,*E*)-7-(((*tert*-butyldimethylsilyl)oxy)methyl)-4-methyl-3,4,4a,5,6,8a-hexahydronaphthalen-1(2*H*)-ylidene)acetate (13)

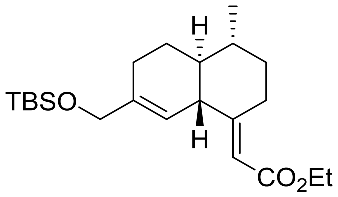

To a stirred solution of the tertiary alcohol **10** (0.64 g, 1.61 mmol) in DCM (30 mL) *Martin Sulfurane* (1.30 g, 1.94 mmol) was added at 0 °C and the solution was stirred for 10 min. The reaction was finished by the addition of saturated $NaHCO_3$ solution (10 mL) and water (20 mL). The phases were separated and the aqueous phase was extracted with DCM (2 × 30 mL). The combined organic phases were washed with saturated NaCl solution (60 mL), dried over Na_2SO_4 and filtered. The solvent was removed under reduced pressure and the crude product was purified by silica gel chromatography (*n*-hexane/EtOAc, 30:1 v/v) to yield **13** (0.58 g, 1.53 mmol, 95%) as colorless oil. TLC (*n*-hexane/EtOAc, 10:1 v/v): $R_f = 0.66$; $[\alpha]_D^{22}$ (deg cm^3 g^{-1} dm^{-1}) = +102.5° (c = 0.0095 in $CHCl_3$); IR (film): $\tilde{\nu}_{max}$ 2953, 2927, 2856, 1715, 1645, 1463, 1380, 1256, 1179, 1162, 1143, 1067, 837 cm^{-1}; ^{1}H-NMR ($CDCl_3$, 300 MHz): δ 5.69-

5.60 (s, 1H), 5.59 (s, 1H), 4.14 (q, *J* = 7.1 Hz, 2H), 4.11 – 4.02 (m, 2H), 3.95 – 3.86 (m, 1H), 2.67 – 2.51 (m, 1H), 2.14 – 2.01 (m, 2H), 1.99 – 1.84 (m, 3H), 1.51 – 1.40 (m, 1H), 1.27 (t, *J* = 7.1 Hz, 3H), 1.30 – 1.14 (m, 3H), 1.03 – 0.95 (m, 1H), 0.92 (s, 9H), 0.91 (d, *J* = 5.8 Hz, 3H), 0.08 (s, 6H); ^{13}C-NMR ($CDCl_3$, 75 MHz): δ 167.3, 165.8, 139.2, 120.6, 110.3, 67.0, 59.5, 49.7, 48.0, 37.8, 37.1, 30.6, 26.9, 25.9, 25.6, 18.9, 18.4, 14.3, −5.2; HRMS (*m/z*): $[M+Na]^+$ calc. for $C_{22}H_{38}O_3SiNa$: 401.24824, found: 401.24569.

Ethyl 2-((1*S*,4*R*,4a*S*,8a*R*)-7-(((*tert*-butyldimethylsilyl)oxy)methyl)-4-methyl-1,2,3,4,4a,5,6,8a-octahydronaphthalen-1-yl)acetate (14)

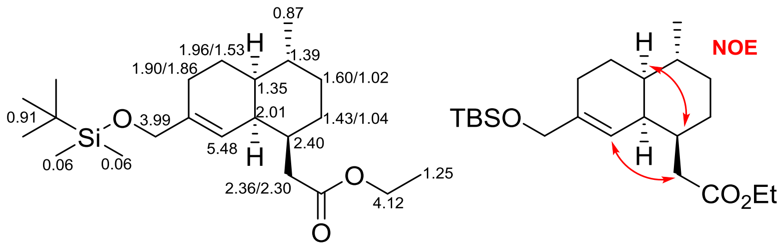

To a stirred solution of **12** (0.48 g, 1.27 mmol) in MeOH (30 mL) $NaBH_4$ (0.19 g, 5.07 mmol) and $NiCl_2$□H_2O (30.1 mg, 0.13 mmol) were added at −60 °C. Afterwards, the mixture was slowly warmed to −40 °C within 1 h. The resulting dark suspension was carefully finished by adding saturated $NaHCO_3$-solution (10 mL). Water (20 mL) and MTBE (50 mL) was added and the phases were separated. The aqueous phase was extracted with MTBE (2 × 30 mL). The combined organic phases were washed with water (50 mL) and saturated NaCl solution (60 mL), dried over Na_2SO_4 and filtered. The solvent was removed under reduced pressure and the crude product was purified by silica gel chromatography (*n*-hexane/EtOAc, 40:1 v/v) to yield **14** (0.47 g, 1.23 mmol, 96%, *d.r.* = 1 : 0.03) as colorless oil. TLC (*n*-hexane/EtOAc, 30:1 v/v): R_f = 0.32; IR (film): $\tilde{\nu}_{max}$ 2961, 2896, 1604, 1585, 1515, 1454, 1322, 1250, 1017, 844 cm^{-1}; ^{1}H-NMR ($CDCl_3$, 300 MHz): δ 5.51 – 5.46 (m, 1H), 4.12 (q, *J* = 7.1 Hz, 2H), 4.00 – 3.98 (m, 2H), 2.51 – 2.21 (m, 3H), 2.11 – 1.97 (m, 2H), 1.96 – 1.84 (m, 2H), 1.65 – 1.56 (m, 1H), 1.52 – 1.40 (m, 3H), 1.37 – 1.33 (m, 1H), 1.27 – 1.22 (m, 2H), 1.25 (t, *J* = 7.1 Hz, 3H), 1.09 – 0.96 (m, 1H), 0.91 (s, 9H), 0.87 (d, *J* = 6.1 Hz, 2H), 0.06 (s, 6H); ^{13}C-NMR ($CDCl_3$, 75 MHz): δ 173.5 (C1), 138.6 (C7’), 120.1 (C8’), 67.5 (C10’), 60.3 (C3), 42.1 (C4a’), 39.9 (C1’), 38.7 (C2), 35.4 (C3’), 26.1 (C7), 25.3 (C5’),

19.9 (C9'), 18.6 (C6), 14.4 (C4), −5.0 (C5), −5.1 (C5); HRMS (*m/z*): $[M+Na]^+$ calc. for $C_{22}H_{40}O_3SiNa$: 403.26389, found: 403.26411.

Ethyl 2-((1*S*,4*R*,4a*S*,8a*S*)-7-(((*tert*-butyldimethylsilyl)oxy)methyl)-4-methyl-1,2,3,4,4a,5,6,8a-octahydronaphthalen-1-yl)acetate (15)

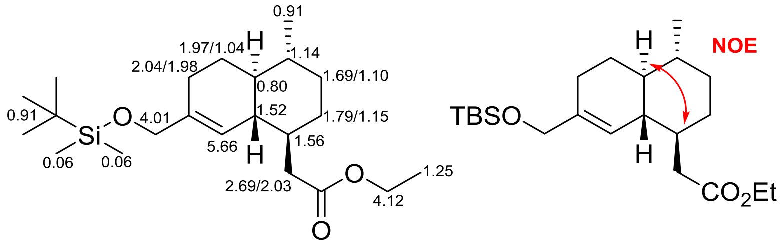

Lithium (5.51 mg, 0.79 mmol) was added in portions to liquid ammonia (10 mL) at −70 °C. The resulting blue suspension was stirred for 30 min at this temperature. Afterwards, the mixture was cooled to −76 °C and EtOH (13.4 mg, 20.1 µL, 0.29 mmol) and the alkene **13** (0.10 g, 0.26 mmol) solved in Et_2O (2 mL) was added. The mixture was stirred for 10 min and saturated NH_4Cl solution (5 mL) was added slowly. The suspension was warmed to room temperature and stirred for 3 h. The phases were separated and the aqueous phase was extracted with MTBE (2 × 10 mL). The combined organic phases were washed with water (20 mL) and saturated NaCl-solution (20 mL), dried over Na_2SO_4 and filtered. The solvent was removed under reduced pressure and the crude product was purified by silica gel chromatography (*n*-hexane/EtOAc, 30:1 v/v) to yield **15** (61.1 mg, 0.16 mmol, 61%, d.r. = 1 : 0.4) as colorless oil. TLC (*n*-hexane/EtOAc, 20:1 v/v): $R_f = 0.48$; IR (film): $\tilde{\nu}_{max}$ 2952, 2927, 2856, 1737, 1463, 1254, 1180, 1069, 837 cm^{-1}; ^{1}H-NMR ($CDCl_3$, 300 MHz): δ 5.70 – 5.61 (m, 1H), 4.12 (q, J = 7.1 Hz, 2H), 4.02 – 3.98 (m, 2H), 2.69 (dd, J = 14.6, 3.4 Hz, 1H), 2.36 – 2.18 (m, 1H), 2.08 – 1.93 (m, 4H), 1.82 – 1.69 (m, 1H), 1.68 – 1.62 (m, 2H), 1.61 – 1.50 (m, 3H), 1.25 (t, J = 7.1 Hz, 3H), 1.21 – 1.04 (m, 2H), 0.91 (d, J = 5.6 Hz, 3H), 0.91 (s, 9H), 0.06 (s, 3H), 0.06 (s, 3H); ^{13}C-NMR ($CDCl_3$, 75 MHz): δ 173.6 (C1), 138.1 (C7'), 122.5 (C8'), 67.5 (C10'), 60.3 (C3), 46.8 (C4a'), 45.4 (C8a'), 39.0 (C2), 38.9 (C1'), 36.5 (C4'), 35.4 (C3'), 33.0 (C2'), 26.3 (C6'), 26.1 (C7), 19.9 (C9'), 18.6 (C6), 14.4 (C4), −5.1 (C5); HRMS (*m/z*): $[M+Na]^+$ calc. for $C_{22}H_{40}O_3SiNa$: 403.26389, found: 403.26331.

Ethyl 2-((1*S*,4*R*,4a*S*,8a*R*)-7-(hydroxymethyl)-4-methyl-1,2,3,4,4a,5,6,8a-octahydronaphthalen-1-yl)acetate (22)

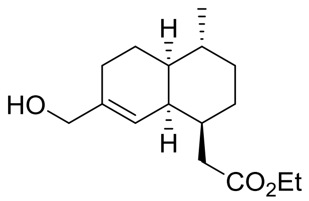

To a stirred solution of **14** (0.50 g, 1.31 mmol) in THF (50 mL) TBAF (2.63 mmol, 1.0 M in THF) was added and the resulting mixture was stirred for 30 min under reflux. Afterwards, the solution was cooled to room temperature, water (50 mL) and MTBE (50 mL) were added and the phases were separated. The aqueous phase was extracted with MTBE (2 × 30 mL). The combined organic phases were washed with water (50 mL) and saturated NaCl solution (50 mL), dried over Na_2SO_4 and filtered. The solvent was removed under reduced pressure and the crude product was purified by silica gel chromatography (DCM/MeOH, 20:1 v/v) to yield **22** (0.33 g, 1.24 mmol, 94%, *d.r.* = 1 : 0.03) as colorless oil. TLC (*n*-hexane/EtOAc, 2:1 v/v): R_f = 0.42; IR (film): $\tilde{\nu}_{max}$ 3437, 2921, 2867, 1734, 1449, 1375, 1263, 1178, 1163, 1032 cm^{-1}; ^{1}H-NMR ($CDCl_3$, 300 MHz): δ 5.56 – 5.46 (m, 1H), 4.13 (q, *J* = 7.1 Hz, 2H), 4.02 – 3.96 (m, 2H), 2.46 – 2.22 (m, 3H), 2.08 – 1.90 (m, 4H), 1.67 – 1.58 (m, 2H), 1.56-1.48 (m, 21H), 1.46 – 1.37 (m, 2H), 1.25 (t, *J* = 7.1 Hz, 3H), 1.24-1.20 (m, 1H), 1.12 – 0.98 (m, 1H), 0.88 (d, *J* = 6.1 Hz, 3H); ^{13}C-NMR ($CDCl_3$, 101 MHz): δ 173.4, 139.0, 121.7, 67.8, 60.4, 41.9, 40.0, 38.7, 38.4, 35.3, 28.7, 27.7, 25.3, 22.2, 19.8, 14.4; HRMS (*m/z*): $[M+Na]^+$ calc. for $C_{16}H_{26}O_3Na$: 289.17742, found: 289.17610, $[2M+Na]^+$ calc. for $C_{32}H_{52}O_6Na$: 555.36561, found: 555.36363.

Ethyl 2-((1*S*,4*R*,4a*S*,8a*S*)-7-(hydroxymethyl)-4-methyl-1,2,3,4,4a,5,6,8a-octahydronaphthalen-1-yl)acetate (23)

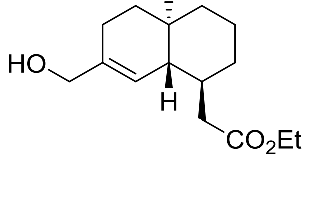

To a stirred solution of **15** (0.17 g, 0.45 mmol) in THF (50 mL) TBAF (0.89 mmol, 1.0 M in THF) was added and the resulting mixture was stirred for 30 min under reflux. Afterwards, the solution was cooled to room temperature, water (30 mL) and MTBE (30 mL) were added and the phases were separated. The aqueous phase was extracted with MTBE (2 × 20 mL). The combined organic phases were washed with water (40 mL) and saturated NaCl-solution (40 mL), dried over Na_2SO_4 and filtered. The solvent was removed under reduced pressure and the crude product was purified by silica gel chromatography (*n*-hexane/EtOAc, 4:1 v/v) to yield **23** (0.11 g, 0.41 mmol, 88%, *d.r.* = 1 : 0.4) as colorless oil. TLC (*n*-hexane/EtOAc, 2:1 v/v): R_f = 0.47; IR (film): $\tilde{\nu}_{max}$ 3417, 2917, 2857, 1734, 1462, 1373, 1183, 1148, 1033 cm^{-1}; ^{1}H-NMR ($CDCl_3$, 300 MHz): δ 5.69-5.54 (m, 1H), 4.13 (q, *J* = 7.2 Hz, 2H), 4.02 – 3.97 (m,2H), 2.68 (dd, *J* = 14.7, 3.8 Hz, 1H), 2.36 – 2.15 (m, 1H), 2.11 – 1.98 (m, 5H), 1.82 – 1.75 (m, 1H), 1.72 – 1.63 (m, 1H), 1.63 – 1.50 (m, 2H), 1.25 (t, *J* = 8.4 Hz, 3H), 1.24-1.20 (m, 2H), 1.18 – 1.06 (m, 3H), 0.91 (d, *J* = 6.1 Hz, 3H), 0.88 – 0.79 (m, 1H); ^{13}C-NMR ($CDCl_3$, 101 MHz): δ 173.7, 138.4, 123.8, 67.5, 60.4, 46.6, 45.6, 39.1, 38.9, 36.5, 35.4, 33.1, 26.5, 26.3, 19.9, 14.4; HRMS (*m/z*): $[M+Na]^+$ calc. for $C_{16}H_{26}O_3Na$: 289.17742, found: 289.17746, $[2M+Na]^+$ calc. for $C_{32}H_{52}O_6Na$: 555.36561, found: 555.36556.

(+)-3-Hydroxymethyl-9-desmethyl-artemisinin (16)

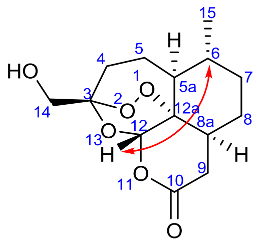

Through a solution of the ester **22** (0.45 g, 1.69 mmol) and methylene blue (~5 mg) in DCM (60 mL) was bubbled a continuous stream of O_2 (~50 mL/min) and the reaction mixture was irradiated with light (150 W) for 30 h at −30 °C. Afterwards, the solution was filtered through a short silica gel column chromatography (EtOAc) and all volatile components were removed und reduced pressure (30 °C). The crude hydroperoxide was dissolved in DCM (40 mL) and some drops of TFA were added at 0 °C. The resulting solution was stirred under room temperature under an O_2 atmosphere for

further two days. Saturated $NaHCO_3$ solution (15 mL) and water (30 mL) were added. The phases were separated and the aqueous phase was extracted with DCM (2 × 50 mL). The combined organic phases washed with water (50 mL) and saturated NaCl solution (50 mL), dried over Na_2SO_4 and filtered. The solvent was removed under reduced pressure and the crude product was purified by silica gel chromatography (*n*-hexane/EtOAc, 1:1 v/v) to yield **16** (0.12 g, 0.42 mmol, 24%) as colorless oil (the same reaction procedure is performed with ester **23**, 16%). TLC (DCM/MeOH, 5:1 v/v): $R_f = 0.68$; $[\alpha]_D^{22}$ (deg cm^3 g^{-1} dm^{-1}) = +98.1° (c = 0.0080 in $CHCl_3$); IR (film): $\tilde{\nu}_{max}$ 3437, 2953, 2928, 2870, 1738, 1455, 1232, 1214, 1144, 1111, 1069, 1032, 998 cm^{-1}; ^{1}H-NMR ($CDCl_3$, 400 MHz): *δ* 5.97 (s, 1H, H12), 3.62 (d, *J* = 1.5 Hz, 2H, H14), 3.20 (dd, *J* = 18.2, 6.9 Hz, 1H, H9), 2.40 – 2.33 (m, 1H, H4), 2.29 (dd, *J* = 18.3, 1.3 Hz, 1H, H9), 2.20 – 2.12 (m, 1H, H4), 2.10 – 2.02 (m, 1H, H5), 1.96 – 1.89 (m, 1H, H8a), 1.79 – 1.71 (m, 2H, H7, H8), 1.50 – 1.41 (m, 4H, H5, H5a, H6, H8), 1.17 – 1.07 (m, 1H, H7), 1.01 (d, *J* = 5.3 Hz, 3H, H15); ^{13}C-NMR ($CDCl_3$, 101 MHz): *δ* 168.6 (C10), 105.8 (C3), 93.6 (C12), 79.3 (C12a), 65.8 (C14), 50.2 (C5a), 38.8 (C8a), 38.0 (C6), 33.9 (C7), 31.8 (C4), 31.7 (C9), 29.5 (C8), 24.5 (C5), 20.0 (C15); HRMS (*m/z*): $[M+Na]^+$ calc. for $C_{14}H_{20}O_6Na$: 307.11521, found: 307.11535, $[2M+Na]^+$ calc. for $C_{28}H_{40}O_{12}Na$: 591.24120, found: 591.24192.

(+)-3-Triethylsilyloxymethyl-9-desmethyl-artemisinin (24)

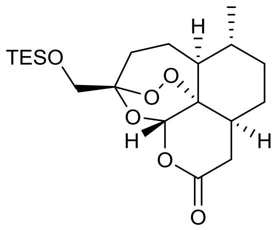

To a solution of **16** (70.1 mg, 0.26 mmol) in DCM (20 mL) chlorotriethylsilane (59.4 mg, 66.1 µL, 0.39 mmol) and NEt_3 (39.9 mg, 54.7 µL, 0.39 mmol) were added at 0 °C. The resulting solution was warmed to room temperature and stirred overnight. Water (20 mL) was added to this orange solution, the phases were separated and the aqueous phase was extracted with DCM (2 × 20 mL). The combined organic phases washed with water (30 mL) and saturated NaCl solution (30 mL), dried over Na_2SO_4 and filtered. The solvent was removed under reduced

pressure and the crude product was purified by silica gel chromatography (*n*-hexane/EtOAc, 5:1 v/v) to yield **24** (81.2 mg, 0.20 mmol, 77%) as colorless oil. TLC (*n*-hexane/EtOAc, 1:1 v/v): R_f = 0.72; $[\alpha]_D^{22}$ (deg cm^3 g^{-1} dm^{-1}) = +45.5° (c = 0.0099 in $CHCl_3$); IR (film): $\tilde{\nu}_{max}$ 2924, 2875, 1745, 1650, 1540, 1458, 1382, 1096, 1003 cm^{-1}; ^{1}H-NMR ($CDCl_3$, 400 MHz): *δ* 5.92 (s, 1H), 3.66 (d, *J* = 11.5 Hz, 1H), 3.57 (d, *J* = 11.5 Hz, 1H), 3.20 (dd, *J* = 18.2, 6.9 Hz, 1H), 2.29 (s, 3H), 2.09 – 2.01 (m, 1H), 1.93 – 1.86 (m, 1H), 1.78 – 1.67 (m, 2H), 1.50 – 1.36 (m, 4H), 1.18 – 1.05 (m, 1H), 1.00 (d, *J* = 5.3 Hz, 3H), 0.94 (t, *J* = 7.9 Hz, 9H), 0.59 (q, *J* = 7.9 Hz, 6H); ^{13}C-NMR ($CDCl_3$, 101 MHz): *δ* 168.8, 106.4, 93.7, 79.0, 66.2, 50.3, 38.9, 38.0, 34.0, 31.7, 31.6, 29.5, 24.4, 20.0, 6.8, 4.4; HRMS (*m/z*): $[M+Na]^+$ calc. for $C_{20}H_{34}O_6SiNa$: 421.20169, found: 421.19867.

(+)-3-Triethylsilyloxymethyl-9-*epi*-methylartemisinin (17)

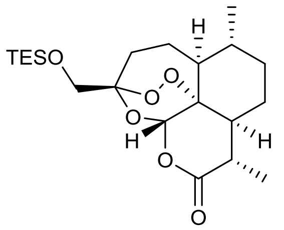

To a solution of diisopropylamine (18.2 mg, 25.3 µL, 0.18 mmol) in THF (20 mL) *n*-BuLi (72.2 µL, 0.18 mmol, 2.5 M in hexane) was added slowly at −60 °C. The solution was warmed slowly to −30 °C and HMPA (32.3 mg, 31.5 µL, 0.18 mmol) was added. The resulting mixture was warmed to −20 °C and afterwards cooled to −78 °C. **24** (55.2 mg, 0.14 mmol) solved in THF (5 mL) was slowly added to the solution and the mixture was warmed to −40 °C within 1 h. Then, the reaction was cooled again to −78 °C and MeI (29.5 mg, 13.0 µL, 0.21 mmol) was added. The solution was slowly warmed to −50 °C within 1 h and then finished by addition of water (10 mL). The suspension was extracted with MTBE (3 × 20 mL). The combined organic phases washed with water (30 mL) and saturated NaCl solution (30 mL), dried over Na_2SO_4 and filtered. The solvent was removed under reduced pressure and the crude product was purified by silica gel chromatography (*n*-hexane/EtOAc, 2:1 v/v) to yield **17** (53.9 mg, 0.13 mmol, 95%) as colorless oil. TLC (*n*-hexane/EtOAc, 3:1 v/v): R_f = 0.46; $[\alpha]_D^{22}$ (deg cm^3 g^{-1} dm^{-1}) = +23.5° (c = 0.0098 in $CHCl_3$); IR (film):

$\tilde{\nu}_{max}$ 2954, 2924, 2874, 1746, 1460, 1378, 1103, 1002 cm^{-1}; ^{1}H-NMR ($CDCl_3$, 400 MHz): δ 5.93 (s, 1H), 3.68 (d, J = 11.4 Hz, 1H), 3.56 (d, J = 11.5 Hz, 1H), 2.32 – 2.24 (m, 3H), 2.05-1.95 (m, 1H), 1.83 – 1.75 (m, 1H), 1.72-1.65 (m, 3H), 1.47 (d, J = 7.5 Hz, 3H), 1.46 – 1.37 (m, 3H), 1.17 (s, 1H), 1.00 (d, J = 5.8 Hz, 3H), 0.94 (t, J = 7.9 Hz, 9H), 0.59 (q, J = 7.8 Hz, 6H); ^{13}C-NMR ($CDCl_3$, 101 MHz): δ 172.6, 106.2, 94.0, 81.2, 66.4, 50.7, 45.6, 39.9, 37.8, 34.2, 31.6, 31.3, 24.4, 20.7, 20.1, 6.8, 4.4; HRMS (m/z): [M+Na]$^{+}$ calc. for $C_{21}H_{36}O_6SiNa$: 435.21734, found: 435.21728, [2M+Na]$^{+}$ calc. for $C_{42}H_{72}O_{12}Si_2Na$: 847.44545, found: 847.44672.

(+)-3-Hydroxymethyl-9-*epi*-methylartemisinin (18)

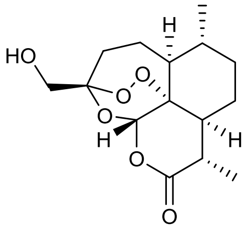

To a stirred solution of **17** (45.0 mg, 0.11 mmol) in THF (10 mL) TBAF (0.14 mL, 0.14 mmol, 1.0 M in THF) was added at 0 °C and the resulting was stirred for 10 min at this temperature. Water (10 mL) was added and the mixture was extracted with EtOAc (3 × 20 mL). The combined organic phases washed with water (30 mL) and saturated NaCl solution (30 mL), dried over Na_2SO_4 and filtered. The solvent was removed under reduced pressure and the crude product was purified by silica gel chromatography (n-hexane/EtOAc, 1:1 v/v) to yield **18** (31.5 mg, 0.10 mmol, 95%) as colorless solid. TLC (n-hexane/EtOAc, 1:1 v/v): R_f = 0.27; $[\alpha]_D^{22}$ (deg cm^3 g^{-1} dm^{-1}) = +55.3° (c = 0.0095 in $CHCl_3$); T_m = 156 °C; IR (KBr): $\tilde{\nu}_{max}$ 3440, 2945, 2855, 1732, 1636, 1469, 1378, 1205, 1110, 1032, 991 cm^{-1}; ^{1}H-NMR ($CDCl_3$, 300 MHz): δ 5.98 (s, 1H), 3.62 (d, J = 2.1 Hz, 2H), 2.37 – 2.23 (m, 2H), 2.20 – 2.12 (m, 1H), 2.06 – 1.98 (m, 1H), 1.89-1.79 (m, 1H), 1.76 – 1.62 (m, 2H), 1.52 – 1.32 (m, 2H), 1.46 (d, J = 7.4 Hz, 4H), 1.27 – 1.18 (m, 2H), 1.17 – 1.07 (m, 1H), 1.00 (d, J = 6.0 Hz, 3H); ^{13}C-NMR ($CDCl_3$, 75 MHz): δ 172.5, 105.6, 93.9, 81.6, 65.9, 50.6, 45.6, 39.8, 37.8, 34.1, 31.9, 31.3, 24.4, 20.6, 20.0; HRMS (m/z): [M+Na]$^{+}$ calc. for $C_{15}H_{22}O_6Na$: 321.13086, found: 321.13054, [M+K]$^{+}$ calc. for $C_{15}H_{22}O_6K$: 337.10480, found: 337.10472, [2M+Na]$^{+}$ calc. for $C_{30}H_{44}O_{12}Na$: 619.27250, found: 619.27252.

(+)-3-Hydroxymethylartemisinin (2)

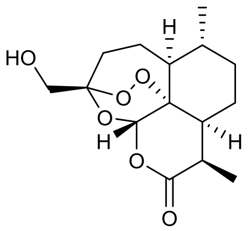

To a solution of **17** (13.1 mg, 31.7 µmol) in DCM (10 mL) 1,8-diazabicyclo[5.4.0]undec-7-ene (9.67 mg, 63.5 µmol) was added and the reaction mixture was stirred for 1 d at room temperature. Water (10 mL) was added and the mixture was extracted with MTBE (2 × 10 mL). The combined organic phases washed with water (30 mL) and saturated NaCl solution (30 mL), dried over Na_2SO_4 and filtered. The solvent was removed under reduced pressure and the crude product was purified by silica gel chromatography (*n*-hexane/EtOAc, 3:1 v/v). The product was solved in THF (10 mL) and at 0 °C TBAF (41.3 µL, 41.3 µmol, 1.0 M in THF) was added. The resulting solution stirred for 10 min, water was added (10 mL) and the mixture was extracted with EtOAc (2 × 10 mL). The combined organic phases washed with water (30 mL) and saturated NaCl solution (30 mL), dried over Na_2SO_4 and filtered. The solvent was removed under reduced pressure and the crude product was purified by silica gel chromatography (*n*-hexane/EtOAc, 1:1 v/v) to yield **2** (8.21 mg, 27.5 µmol, 87% over two steps) as colorless solid. TLC (*n*-hexane/EtOAc, 1:1 v/v): R_f = 0.31; $[\alpha]_D^{22}$ (deg cm^3 g^{-1} dm^{-1}) = +105.4° (c = 0.00100 in $CHCl_3$); T_m = 129 °C; IR (KBr): $\tilde{\nu}_{max}$ 3459, 2925, 2872, 1730, 1647, 1457, 1385, 1210, 1115,6, 1032, 995 cm^{-1}; ^{1}H-NMR ($CDCl_3$, 400 MHz): δ 5.91 (s, 1H), 3.62 (s, 2H), 3.41 (qd, *J* = 7.3, 5.3 Hz, 1H), 2.40 – 2.32 (m, 1H), 2.18 – 2.04 (m, 2H), 1.93 – 1.85 (m, 1H), 1.84 – 1.76 (m, 2H), 1.69 (s, br, 1H), 1.52 – 1.40 (m, 2H), 1.21 (d, *J* = 7.3 Hz, 3H), 1.13 – 1.05 (m, 2H), 1.02 (d, *J* = 5.8 Hz, 3H), 0.91 – 0.82 (m, 1H); ^{13}C-NMR ($CDCl_3$, 101 MHz): δ 171.9, 105.8, 93.6, 80.3, 65.8, 50.2, 45.1, 37.8, 33.7, 33.1, 31.9, 24.6, 23.6, 20.0, 12.7; HRMS (*m/z*): $[M+Na]^+$ calc. for $C_{15}H_{22}O_6Na$: 321.13086, found: 321.13061, $[2M+Na]^+$ calc. for $C_{30}H_{44}O_{12}Na$: 619.27250, found: 619.27133.

Biological studies:

Activity against P. falciparum. In vitro activity against erythrocytic stages of *P. falciparum* was determined using a ^{3}H-hypoxanthine incorporation assay,[4,5] using the drug sensitive NF54 strain[6] (Schipol Airport, The Netherlands) and the standard drugs chloroquine (Sigma C6628) and artesunate (Sigma A3731). Compounds were dissolved in DMSO at 10 mg/mL and further diluted in medium before added to parasite cultures incubated in RPMI 1640 medium without hypoxanthine, supplemented with HEPES (5.94 g/L), $NaHCO_3$ (2.1 g/L), neomycin (100 U/mL), Albumax^R (5 g/L) and washed human red cells A^+ at 2.5% haematocrit (0.3% parasitaemia). Serial drug dilutions of seven 8-fold dilution steps covering a range from 10 to 0.02 µg/mL were prepared. The 96-well plates were incubated in a humidified atmosphere at 37 °C; 4% CO_2, 3% O_2, 93% N_2. After 48 h 50 µL of ^{3}H-hypoxanthine (=0.5 µCi) was added to each well of the plate. The plates were incubated for a further 24 h under the same conditions. The plates were then harvested with a Betaplate™ cell harvester (Wallac, Zurich, Switzerland), and the red blood cells transferred onto a glass fibre filter then washed with distilled water. The dried filters were inserted into a plastic foil with 10 mL of scintillation fluid, and counted in a Betaplate™ liquid scintillation counter (Wallac, Zurich, Switzerland). IC_{50} values were calculated from sigmoidal inhibition curves by linear regression (Huber 1993) using Microsoft Excel. Chloroquine and (+)-artemisinin were used as control.

In vitro cytotoxicity with L-6 cells. Assays were performed in 96-well microtiter plates, each well containing 100 µL of RPMI 1640 medium supplemented with 1% L-glutamine (200 mM) and 10% fetal bovine serum, and 4000 L-6 cells (a primary cell line derived from rat skeletal myoblasts).[7,8] Serial drug dilutions of eleven 3-fold dilution steps covering a range from 100 to 0.002 µg/mL were prepared. After 70 hours of incubation the plates were inspected under an inverted microscope to assure growth of the controls and sterile conditions. 10 µL of Alamar Blue was then

---

[4] Desjardins, R. E.; Canfield, C. J.; Haynes, J. D.; Chulay, J. D. *Antimicrob. Agents Chemother.* **1979**, *16*, 710–718.

[5] Matile H., Pink J. R. L., Plasmodium falciparum malaria parasite cultures and their use in immunology. *In* Lefkovits, I. and B. Pernis (ed.), Immunological Methods. Academic Press, San Diego, **1990**.

[6] Ponnudurai, T.; Leeuwenberg, A. D.; Meuwissen J. H. *Trop. Geogr. Med.* **1981**, *33*, 50–54.

[7] Page, C., Page, M.; Noel, C. *Int. J. Oncol.* **1993**, *3*, 473–476.

[8] Ahmed, S. A.;. Gogal, R. M;. Walsh J. E. *J. Immunol. Methods* **1994**, *170*, 211–224.

added to each well and the plates incubated for another 2 hours. Then the plates were read with a Spectramax Gemini XS microplate fluorometer (Molecular Devices Cooperation, Sunnyvale, CA, USA) using an excitation wave length of 536 nm and an emission wave length of 588 nm. The IC_{50} values were calculated by linear regression[9] from the sigmoidal dose inhibition curves using SoftmaxPro software (Molecular Devices Cooperation, Sunnyvale, CA, USA). Podophyllotoxin (Sigma P4405) was used as control.

[9] W. Huber, J. C. Koella, *Acta Trop*. **1993**, *55*, 257–261.

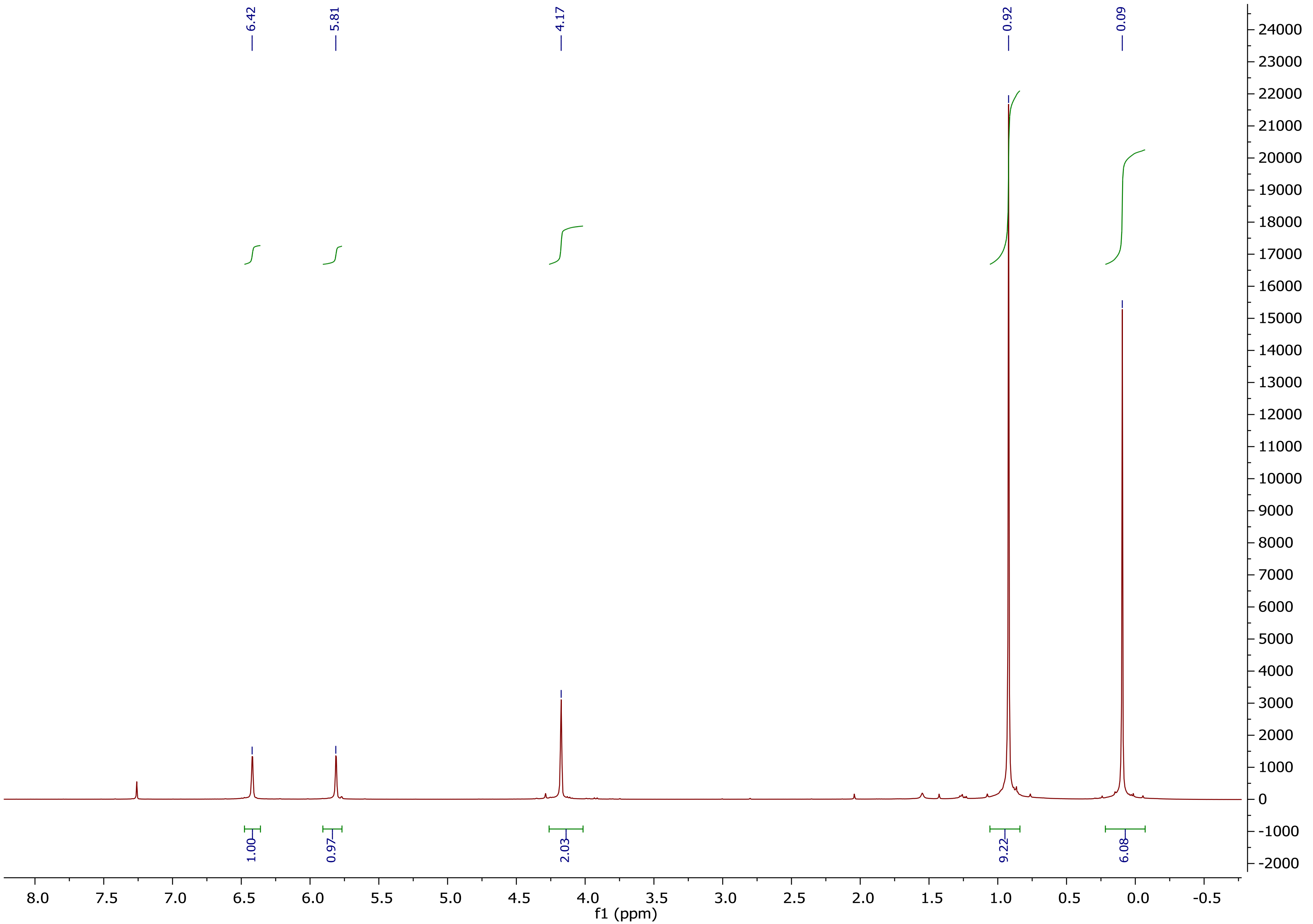

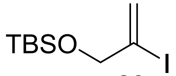

20

($CDCl_3$, 400 MHz)

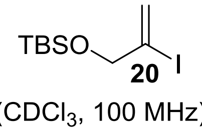

20

(CDCl$_3$, 100 MHz)

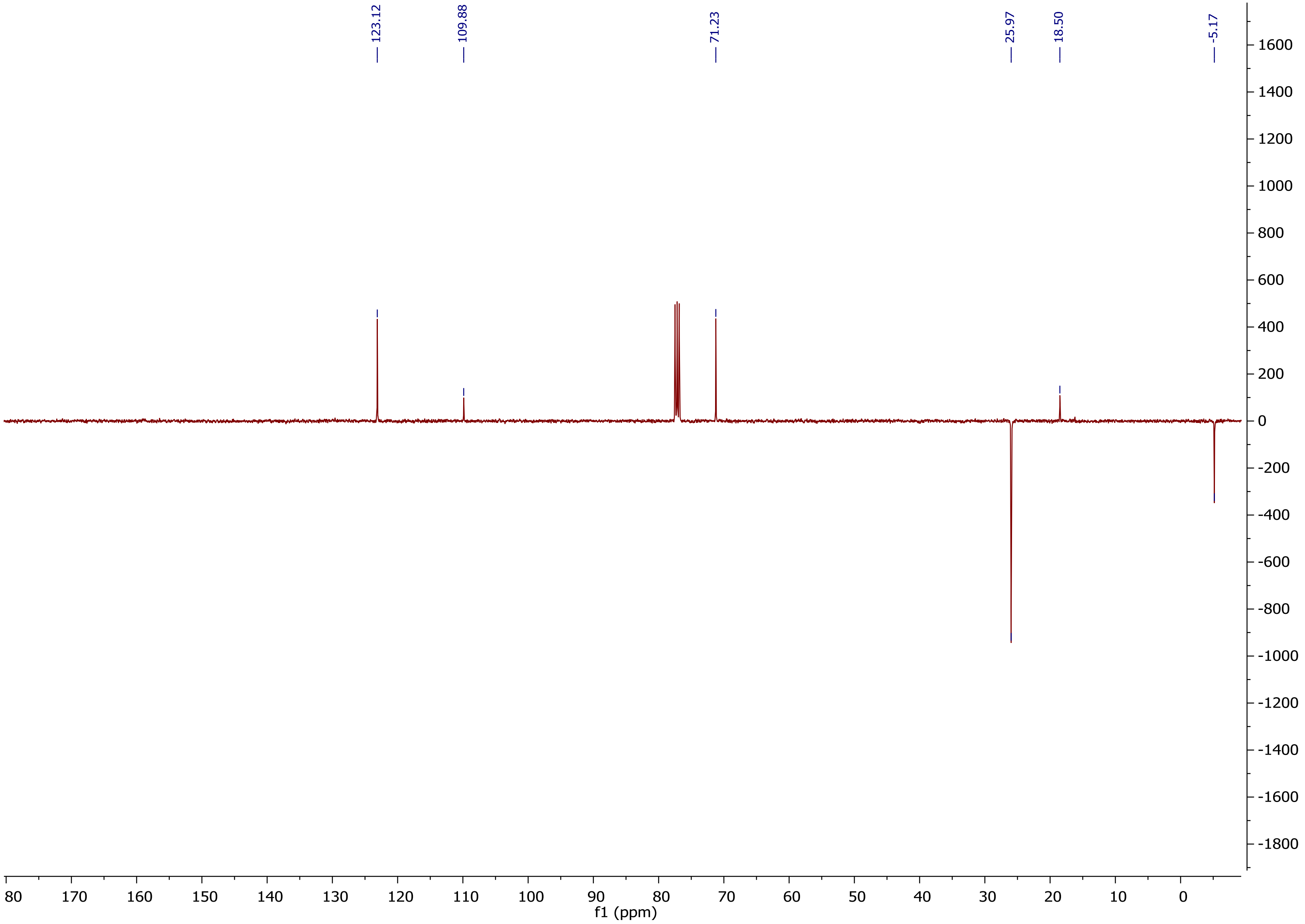

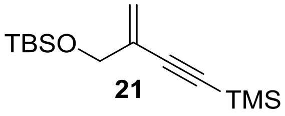

21

($CDCl_3$, 400 MHz)

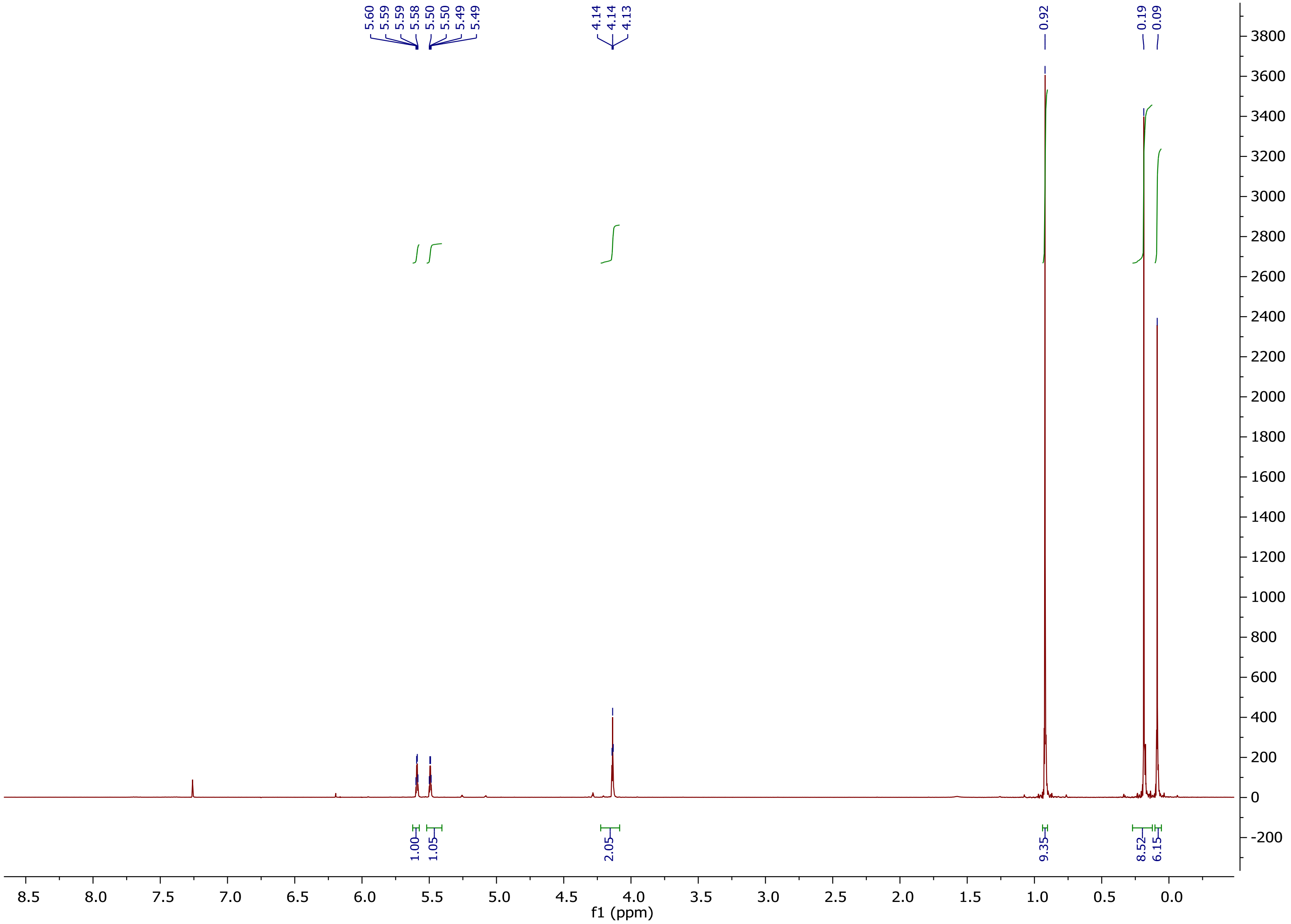

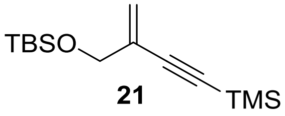

21

($CDCl_3$, 100 MHz)

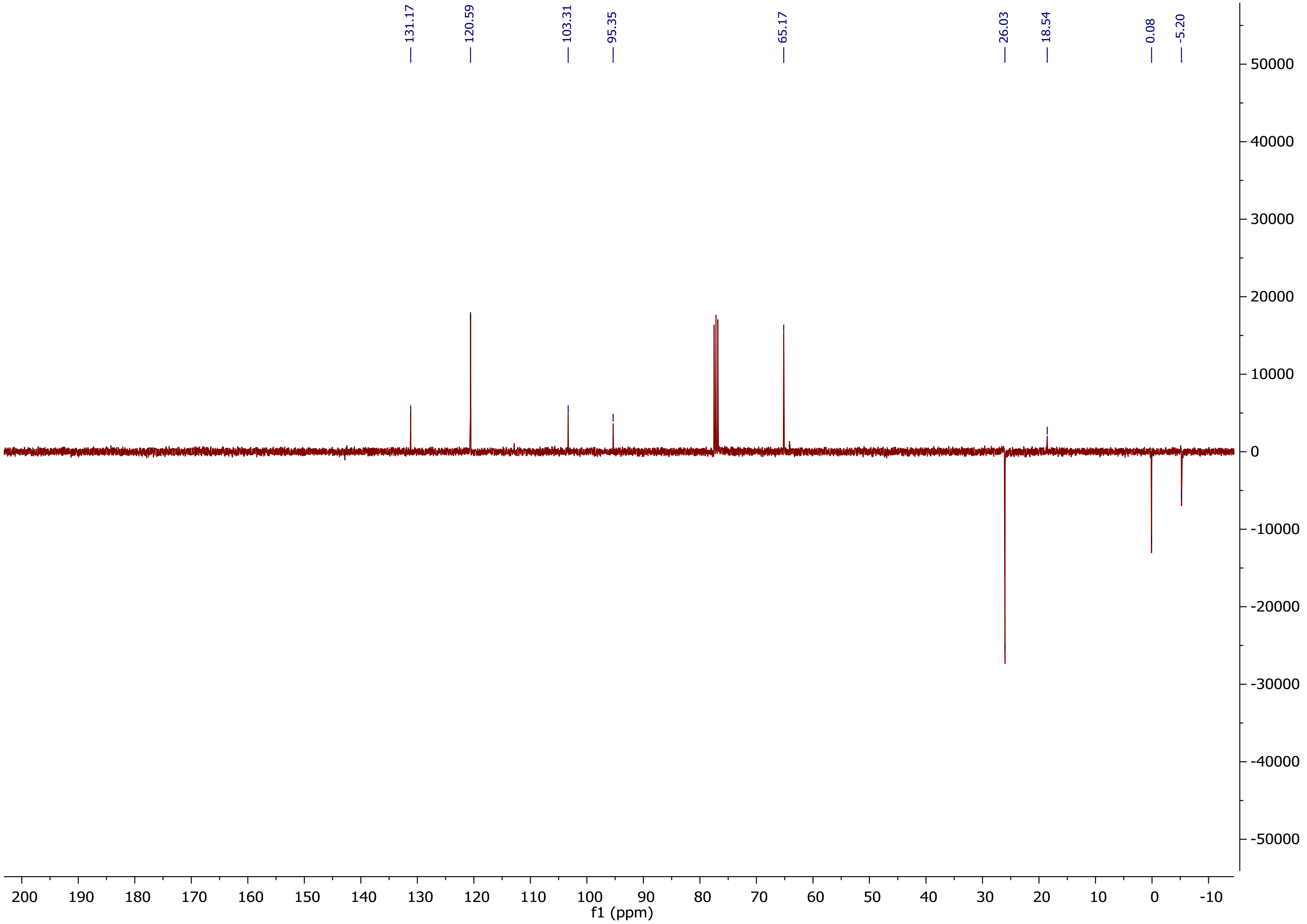

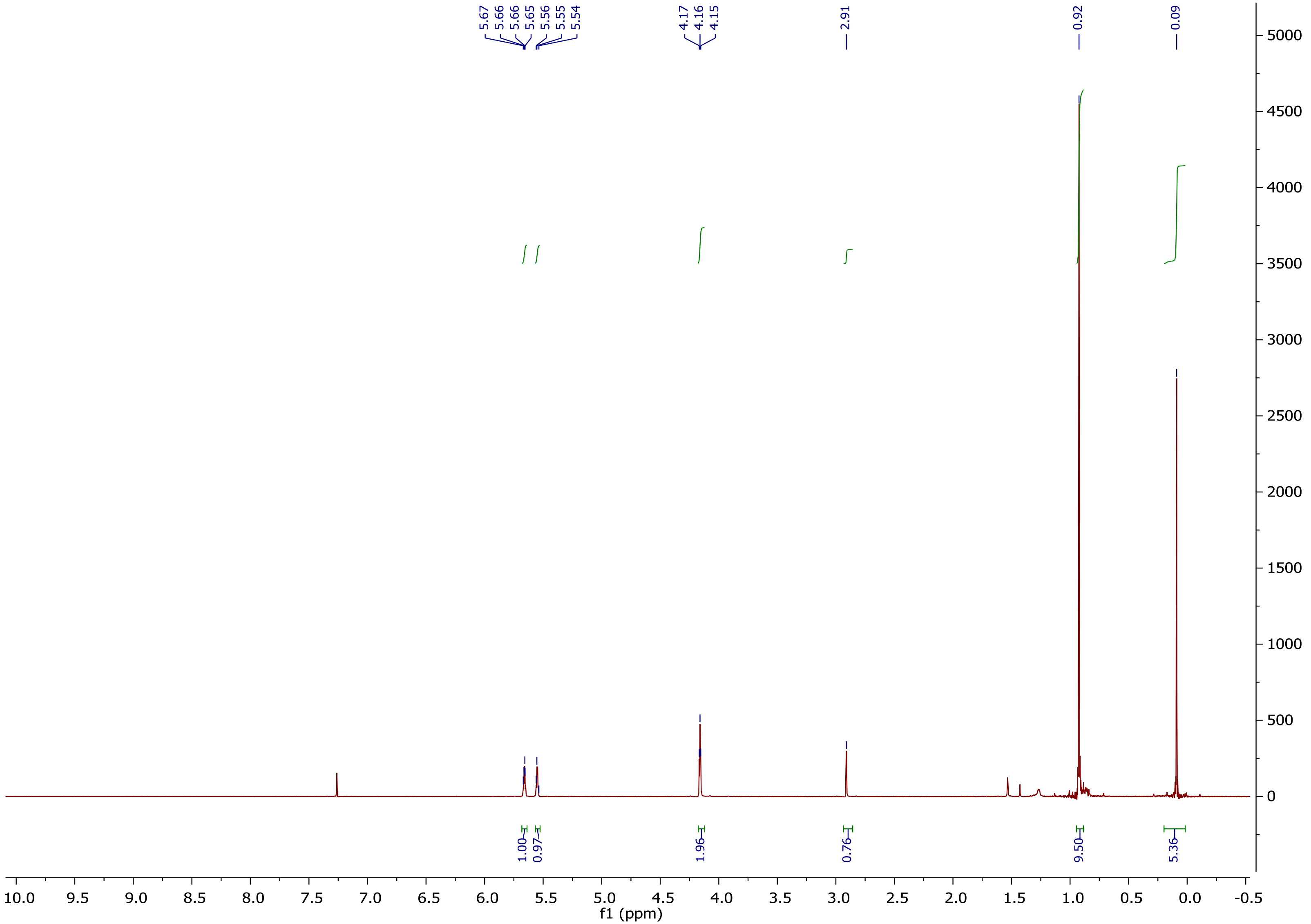

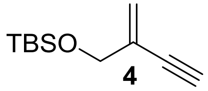

4

($CDCl_3$, 300 MHz)

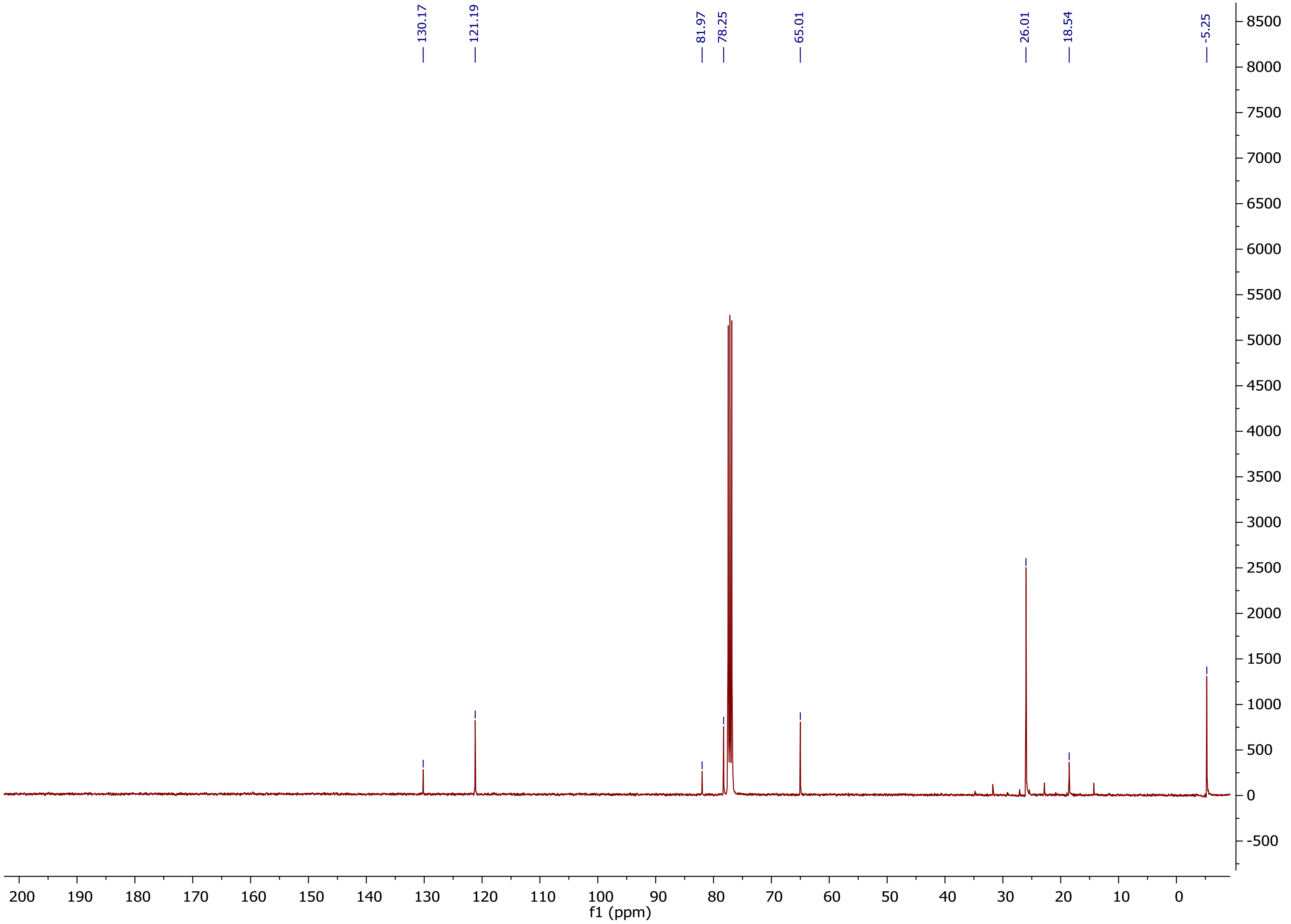

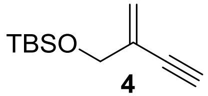

4

($CDCl_3$, 100 MHz)

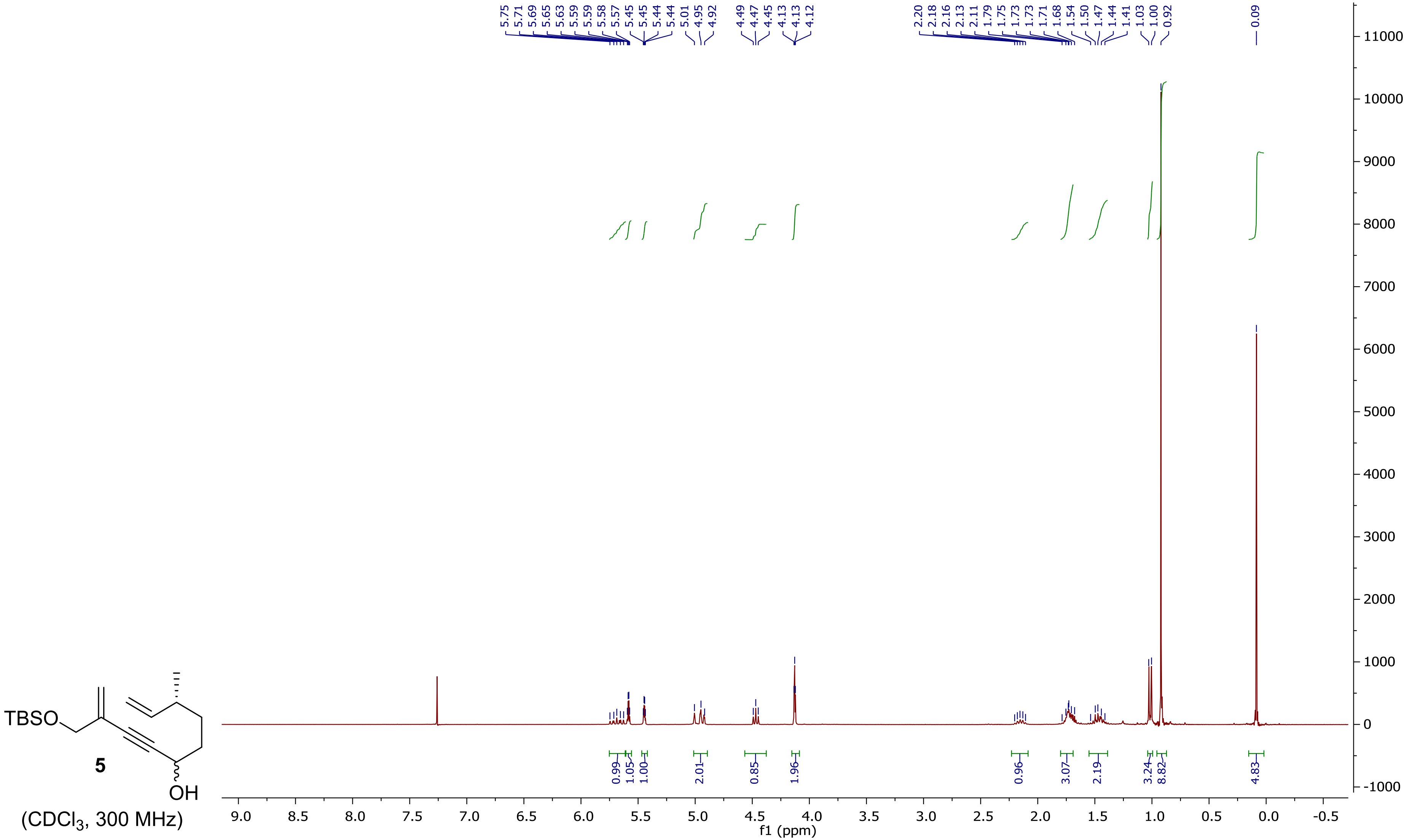

(CDCl$_3$, 300 MHz)

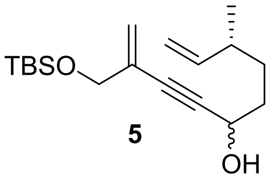

5

($CDCl_3$, 75 MHz)

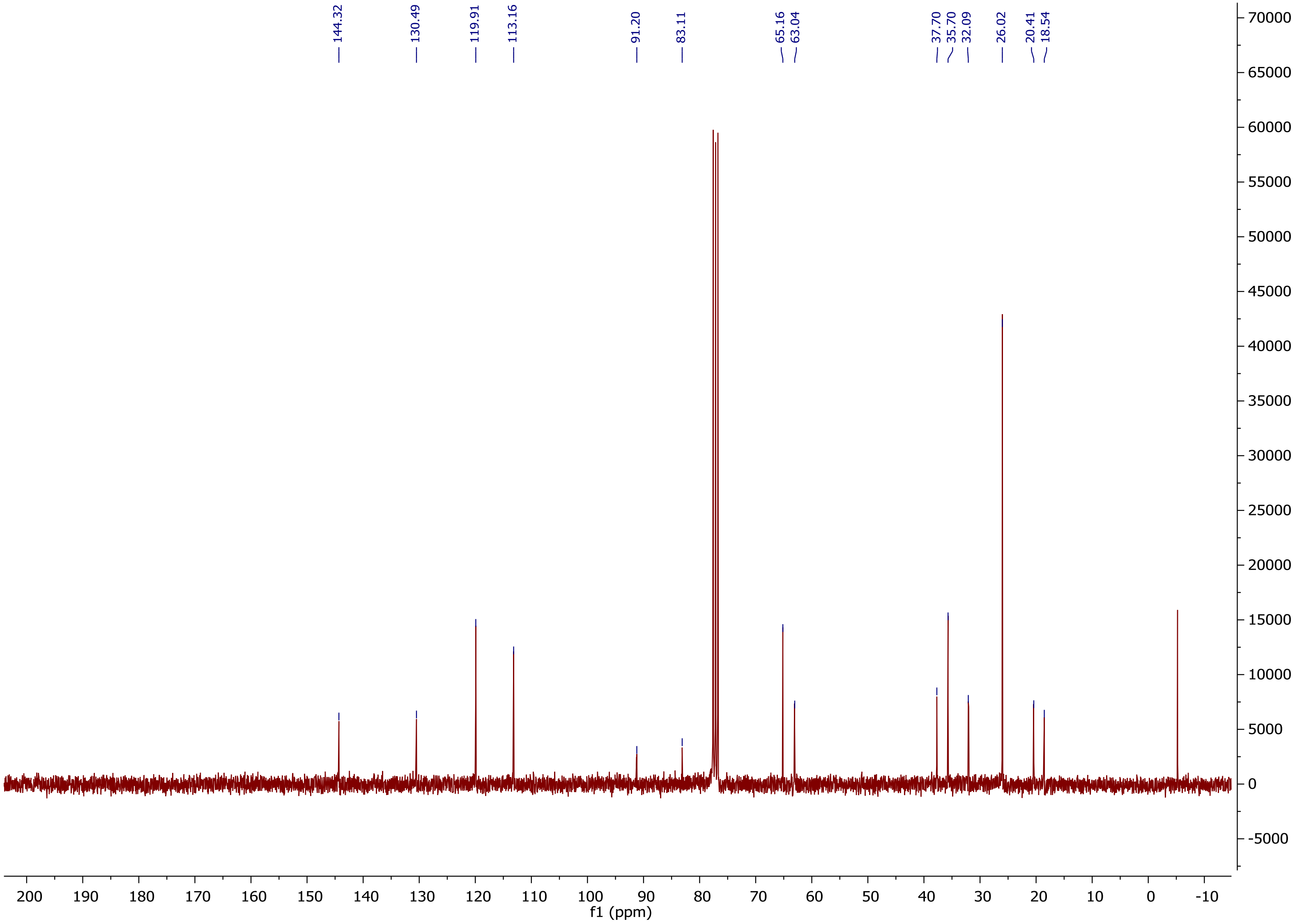

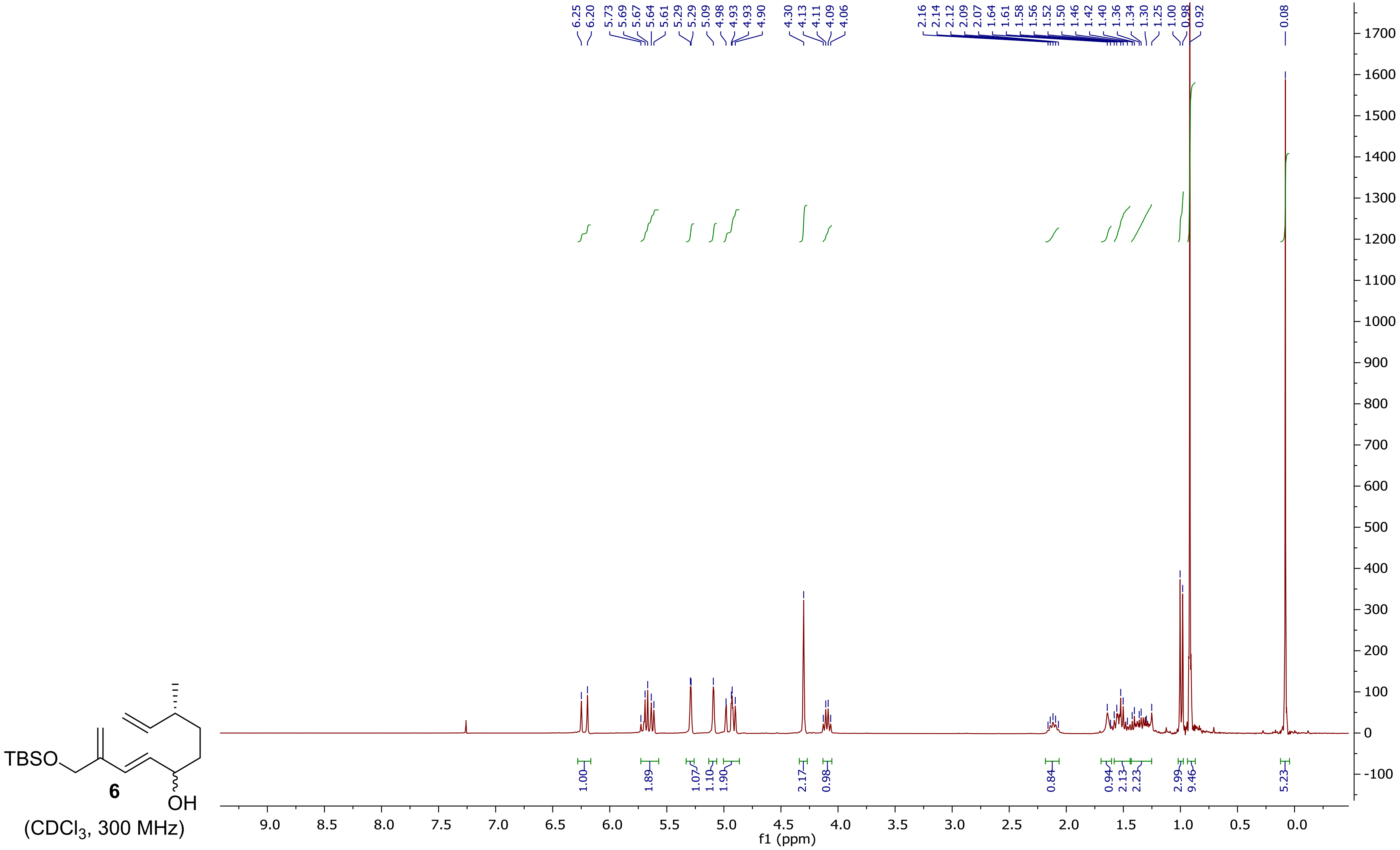

(CDCl$_3$, 300 MHz)

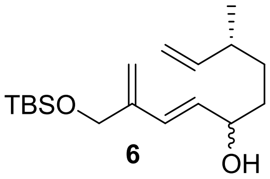

6

($CDCl_3$, 75 MHz)

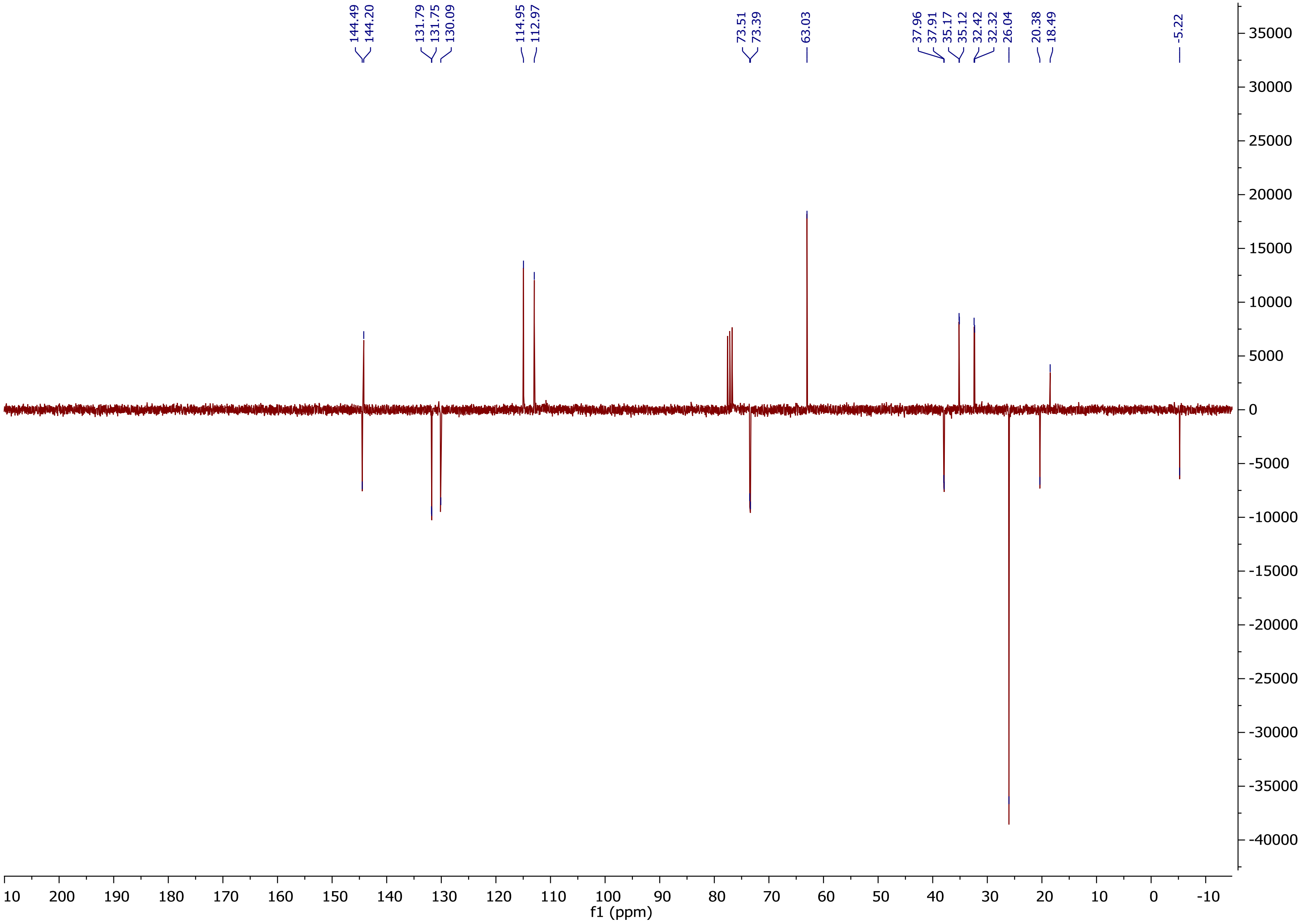

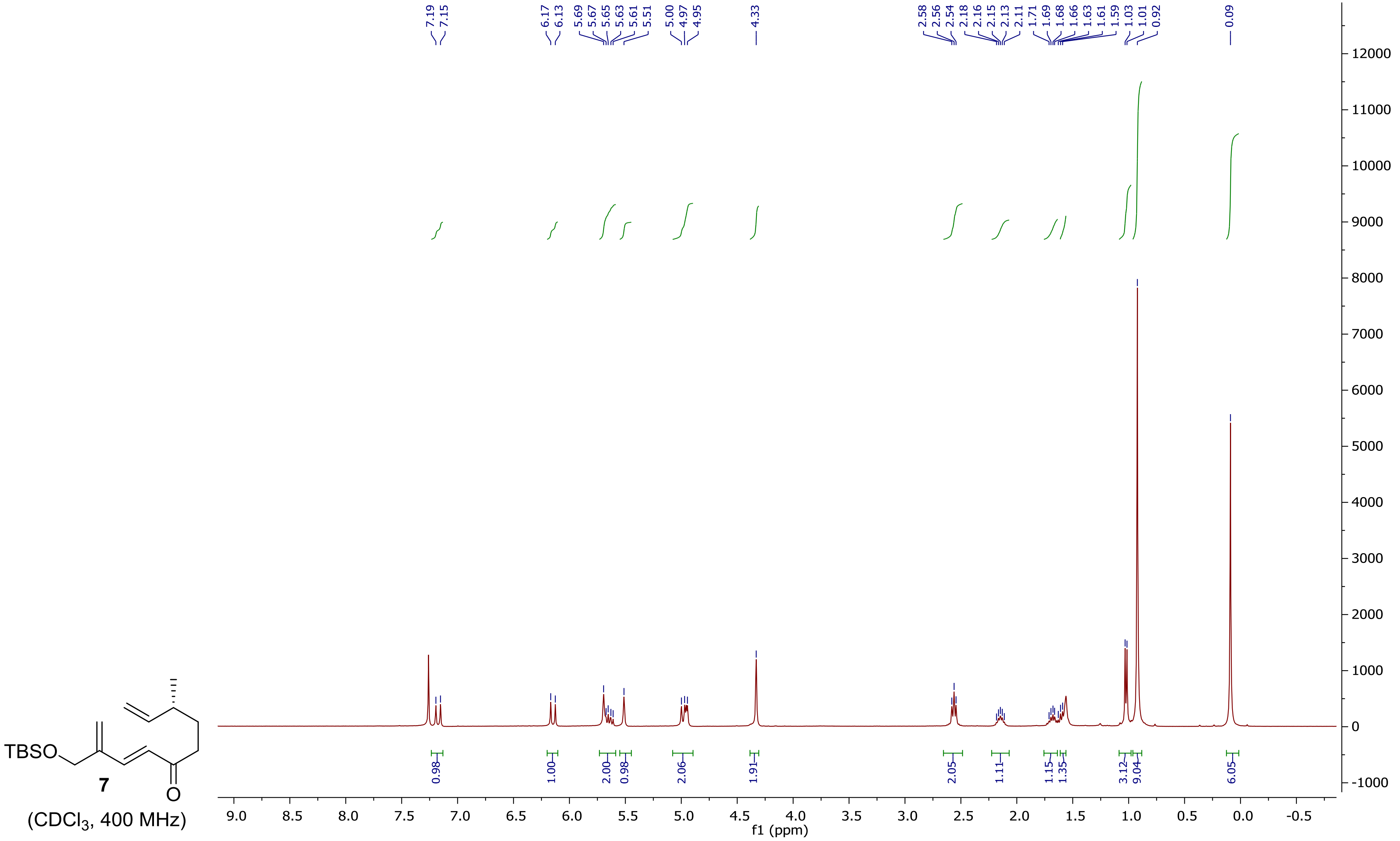

7

($CDCl_3$, 400 MHz)

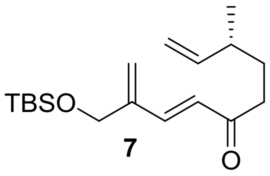

7

(CDCl$_3$, 75 MHz)

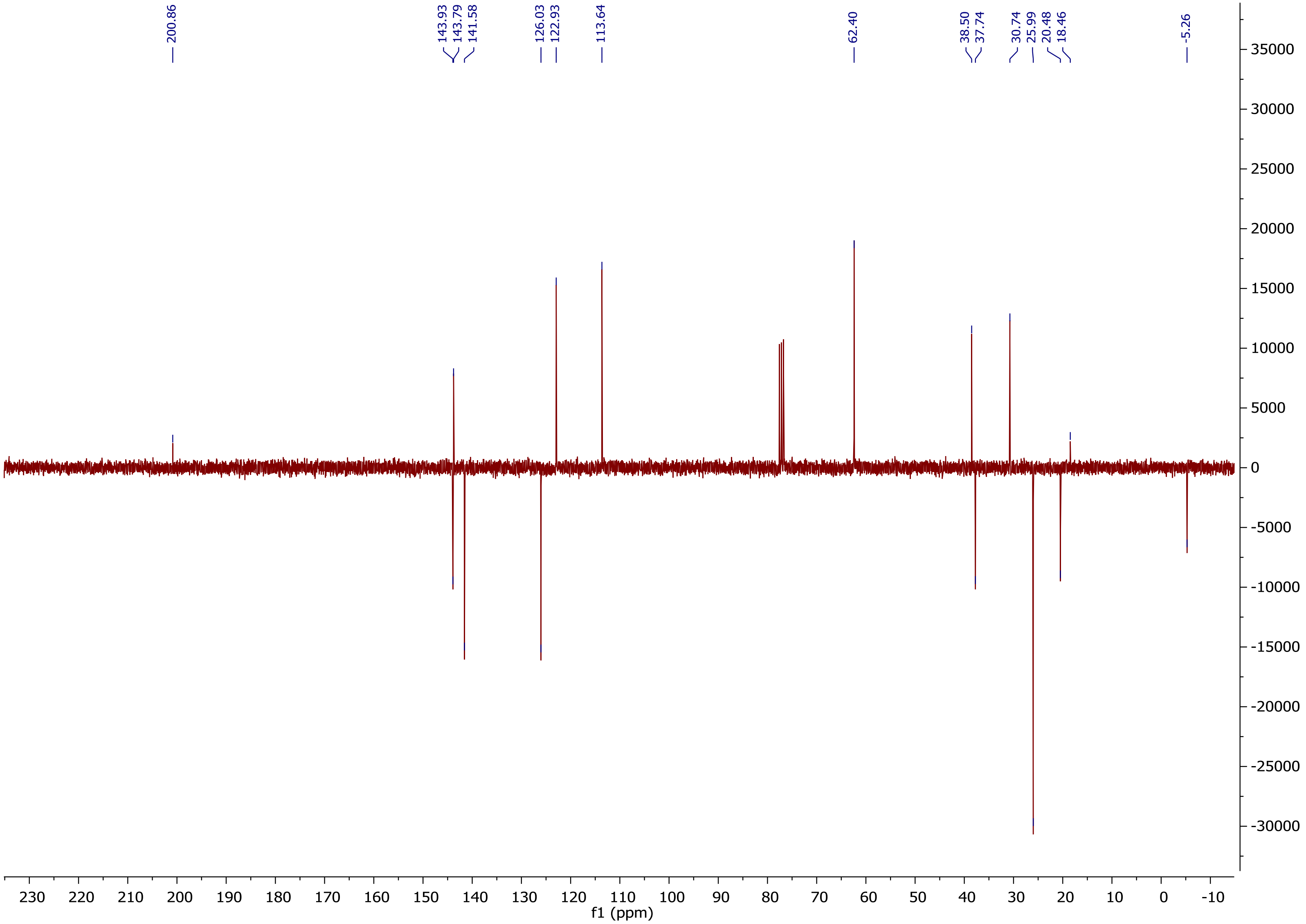

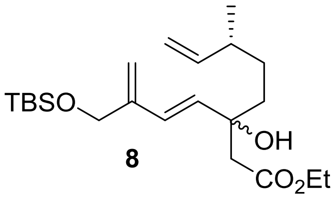

8

($CDCl_3$, 300 MHz)

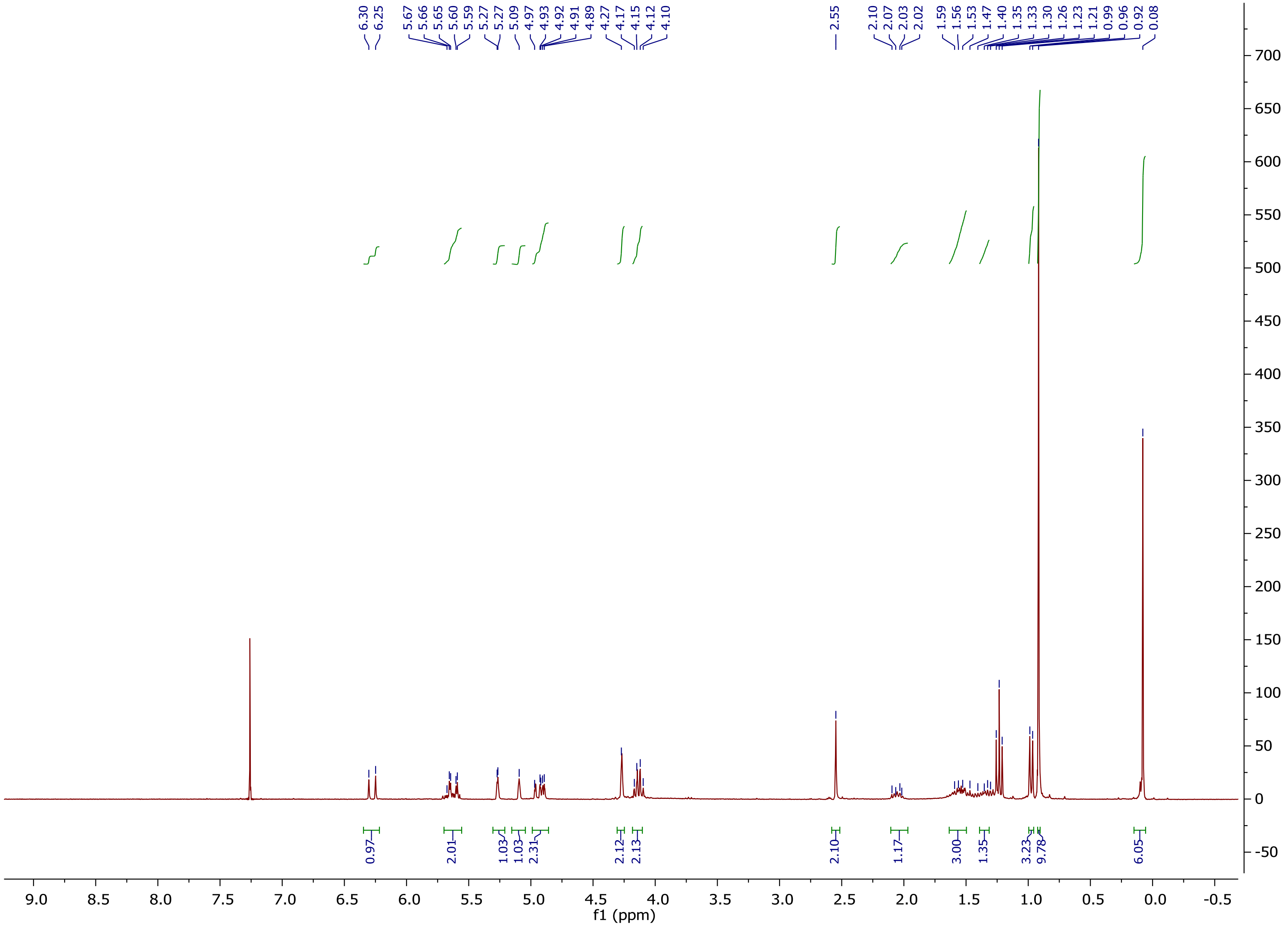

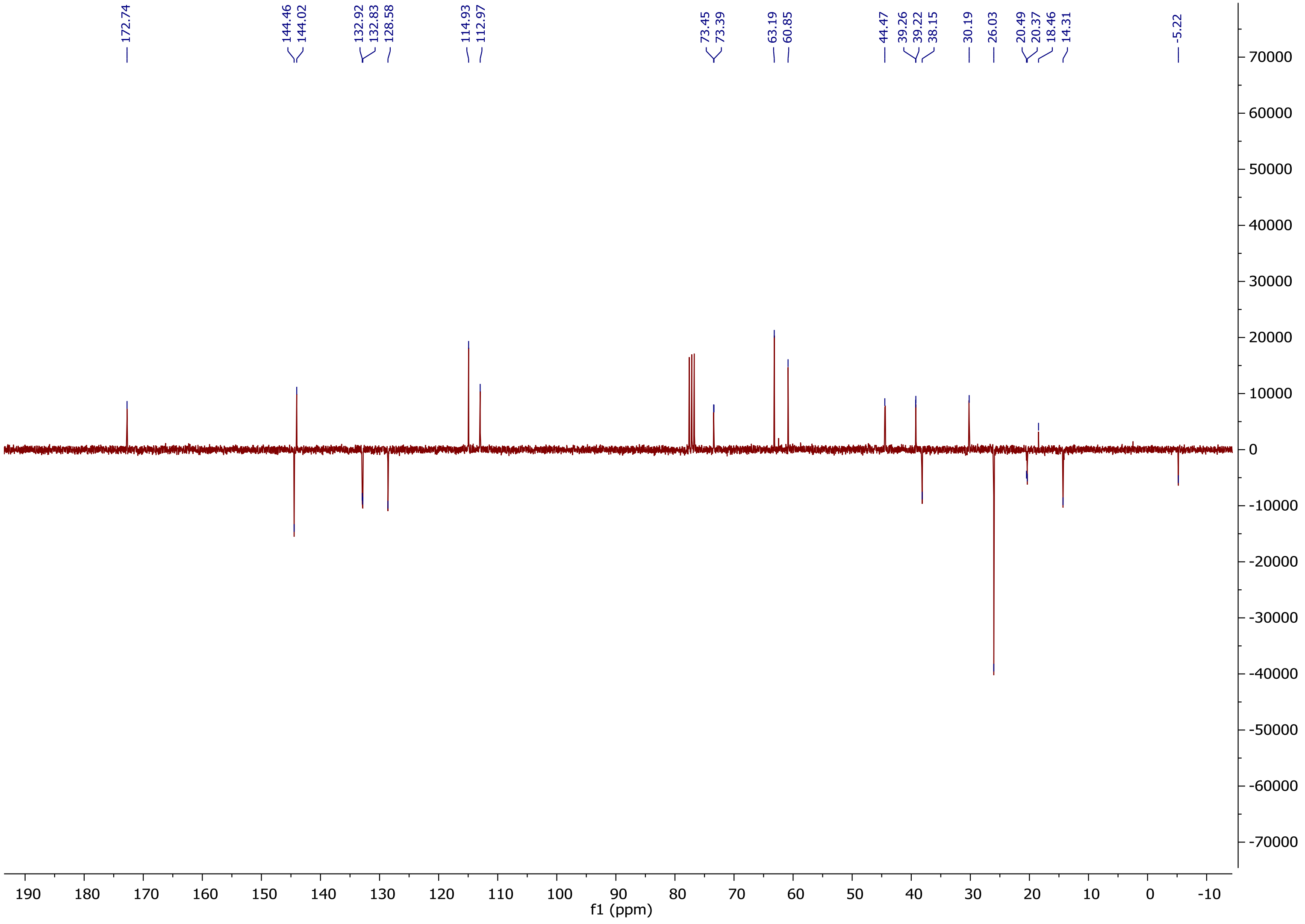

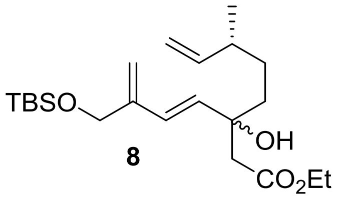

8

($CDCl_3$, 75 MHz)

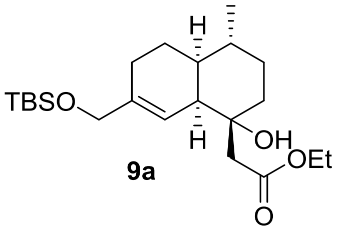

9a

(d$_6$-DMSO, 300 MHz)

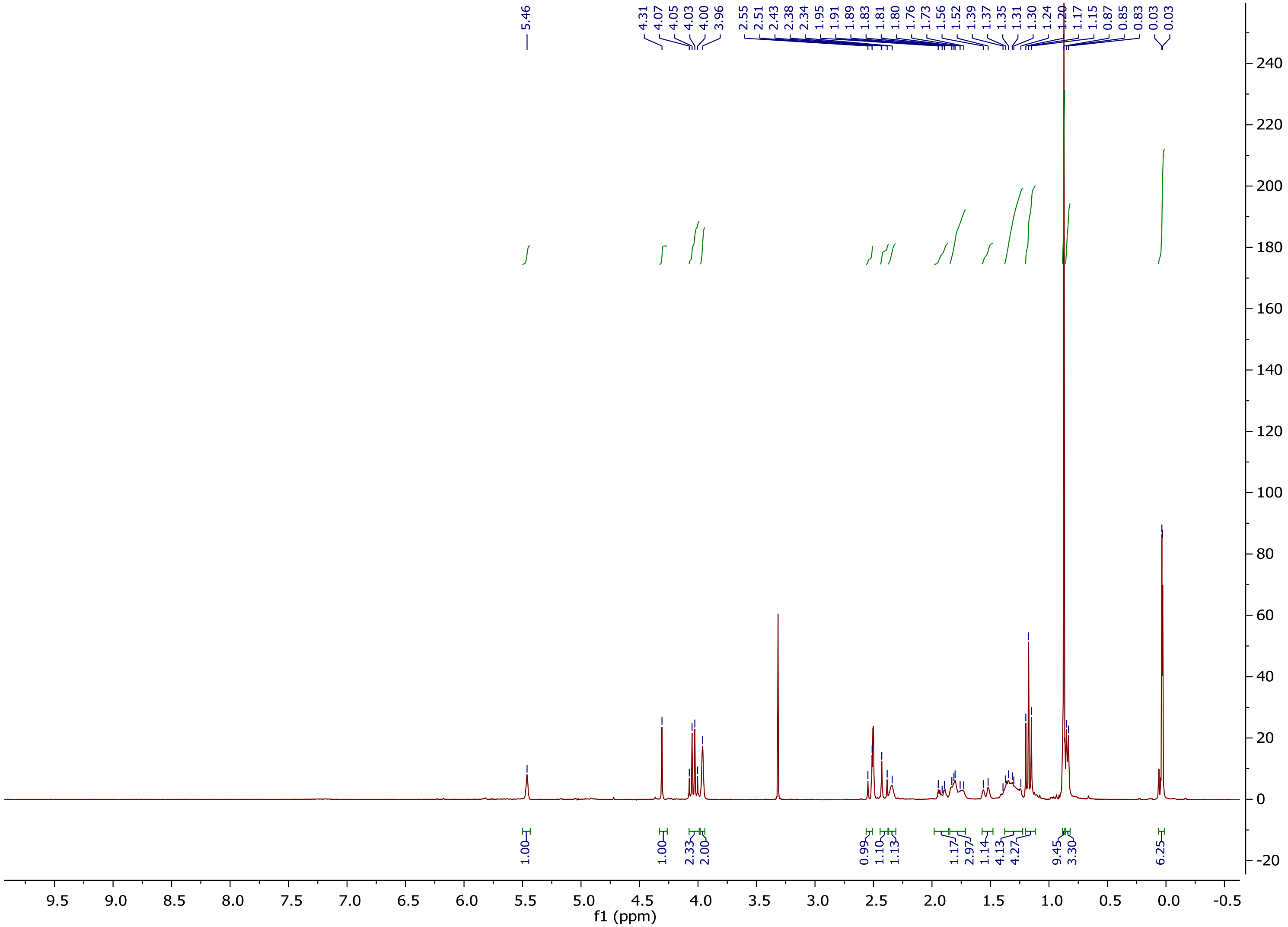

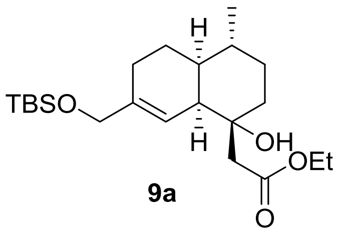

9a

(d$_6$-DMSO, 75 MHz)

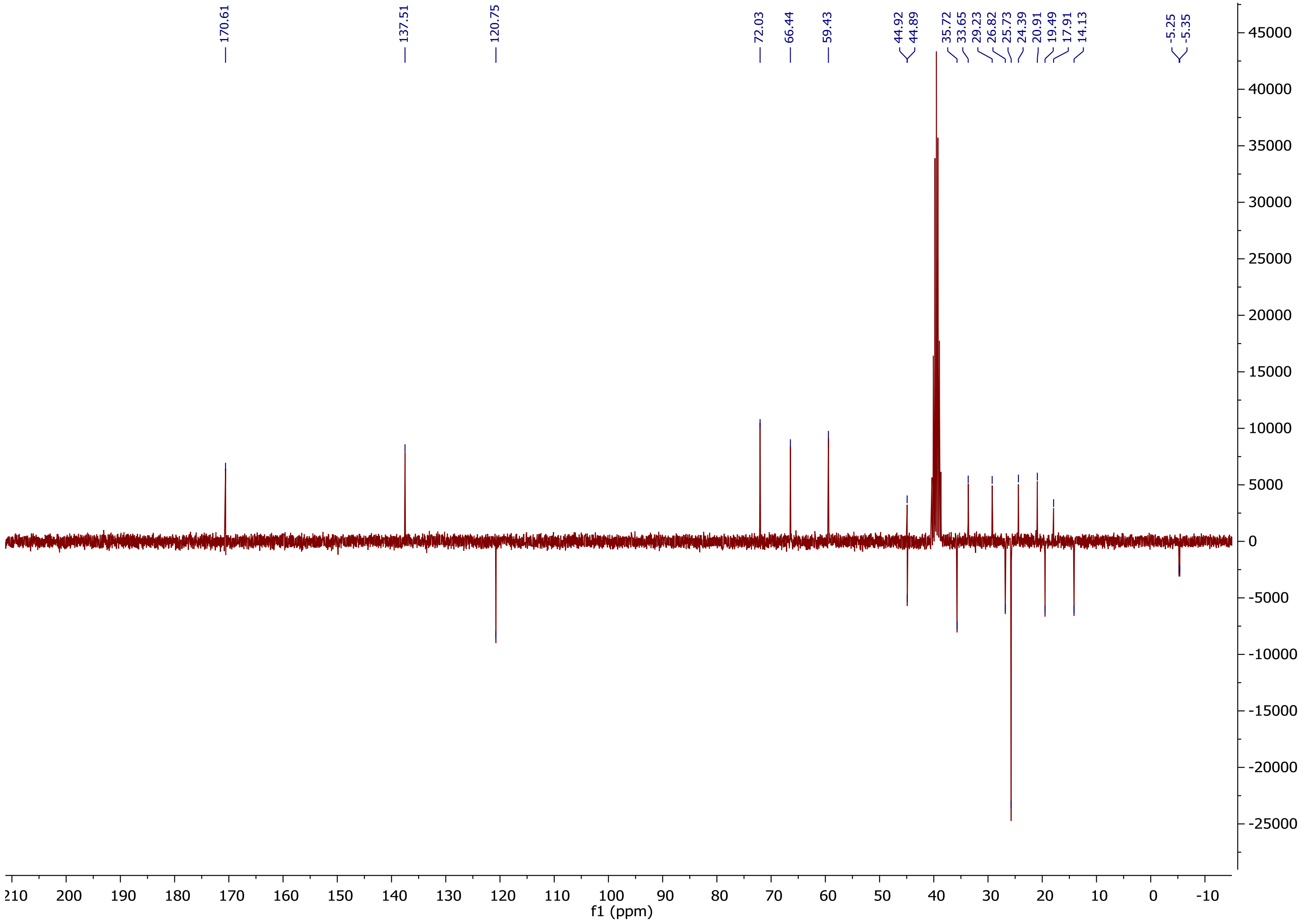

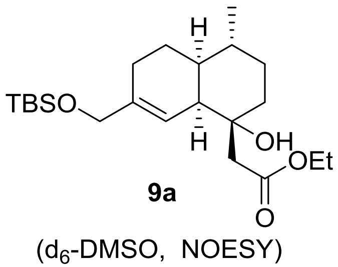

9a

(d_6-DMSO, NOESY)

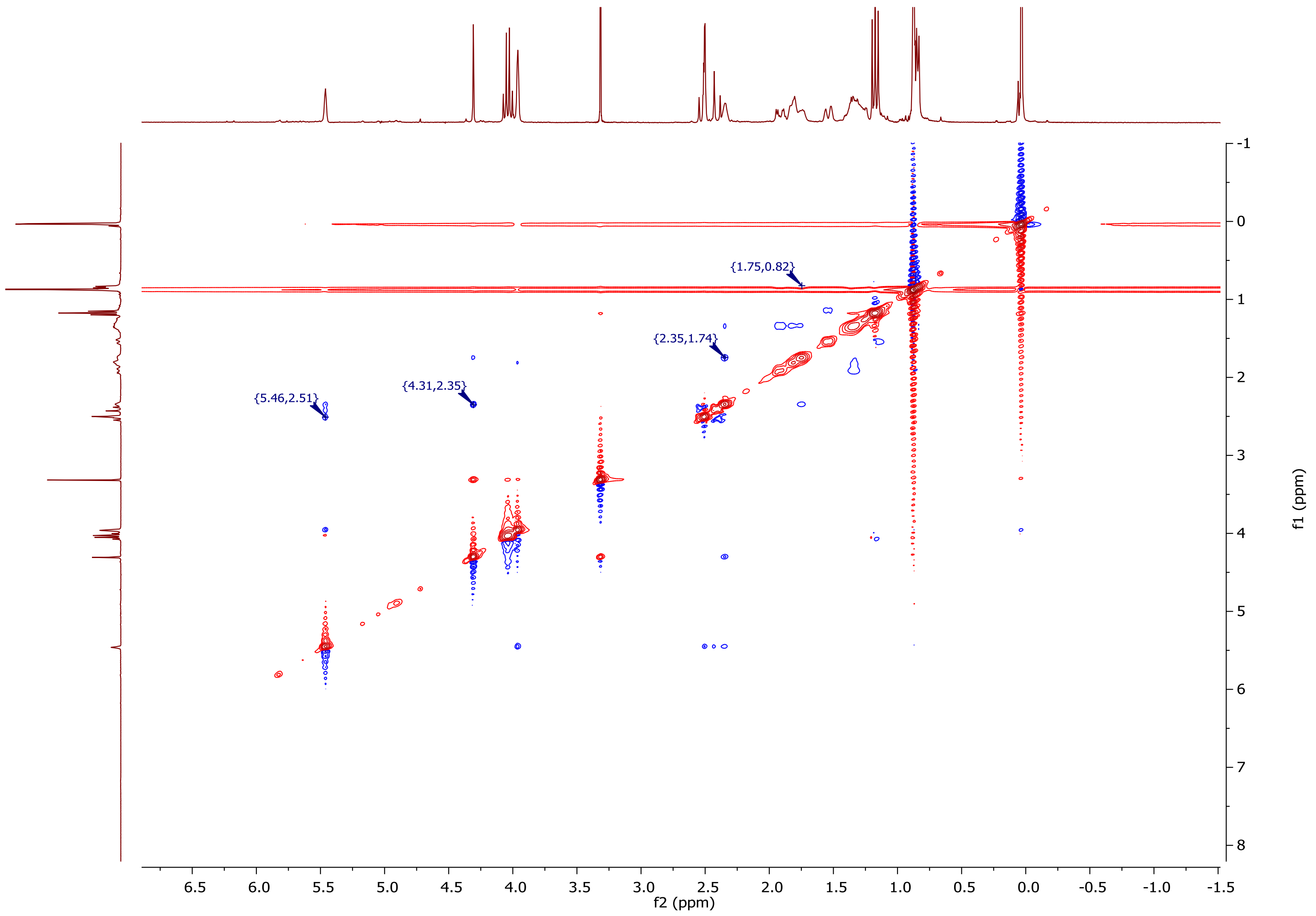

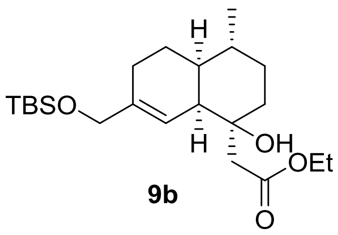

9b

(d$_6$-DMSO, 300 MHz)

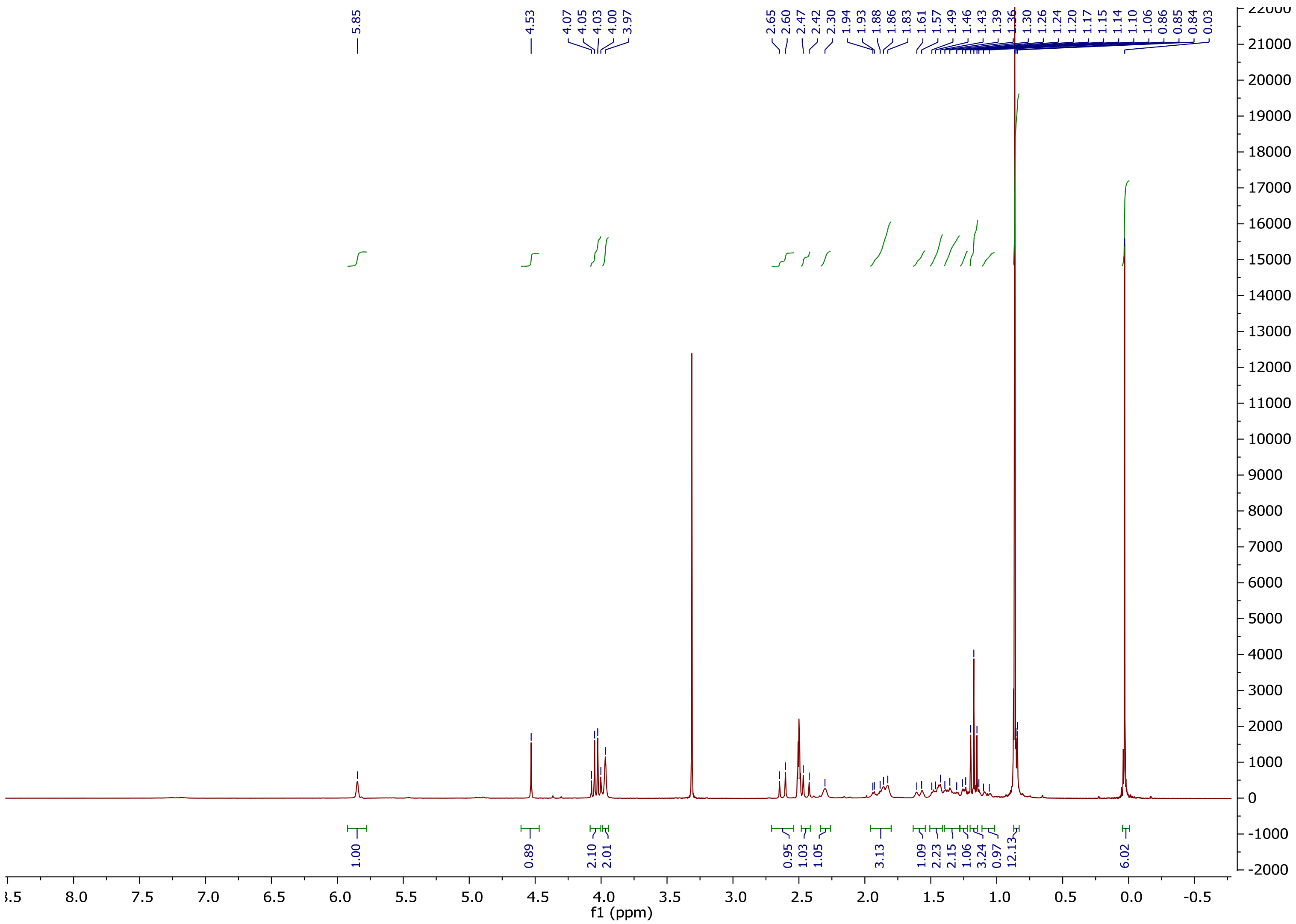

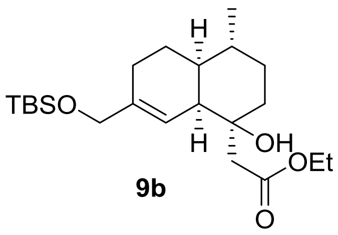

9b

(d$_6$-DMSO, 75 MHz)

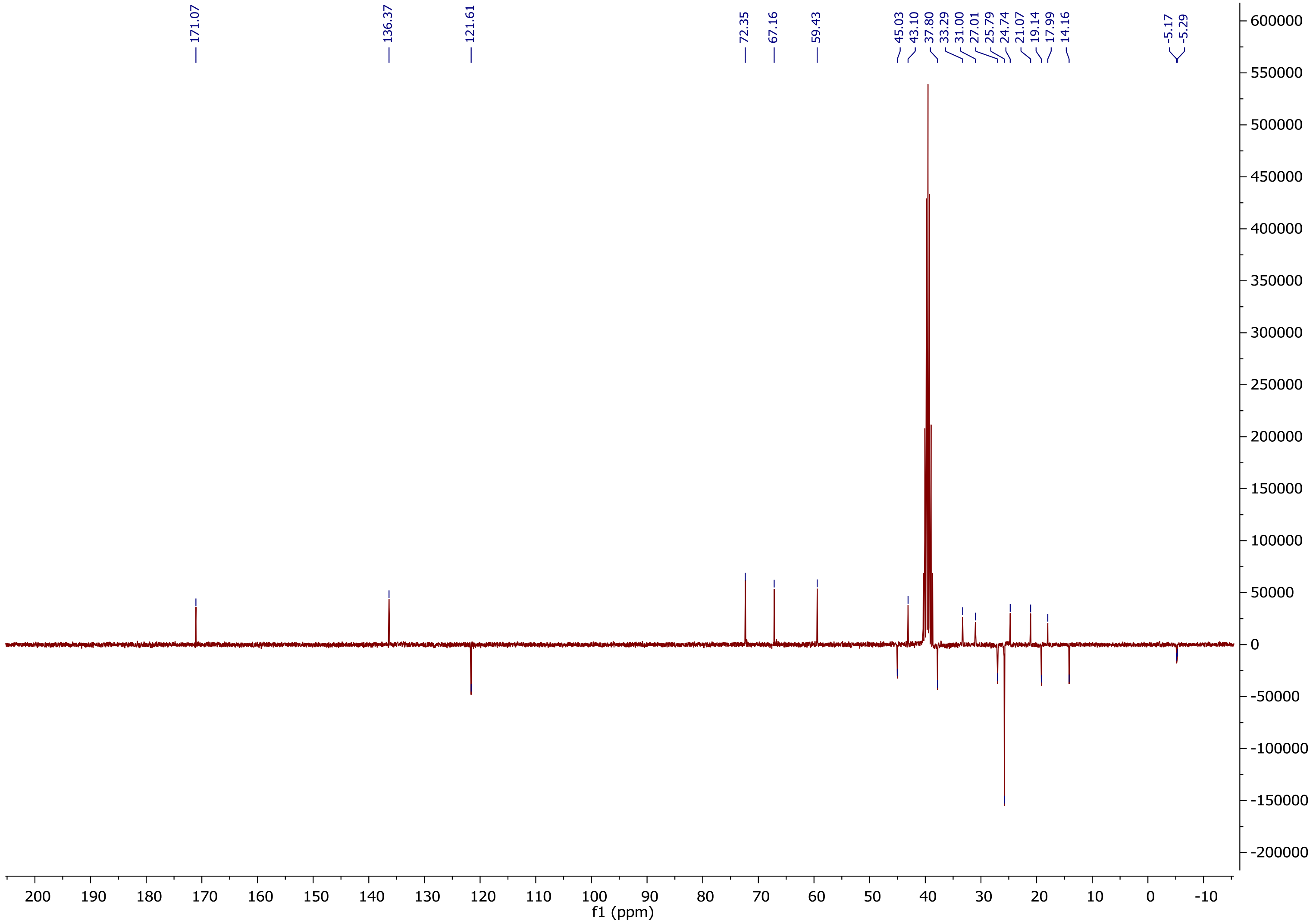

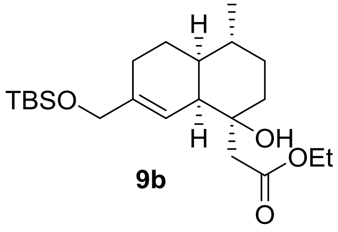

9b

(d_6-DMSO, NOESY)

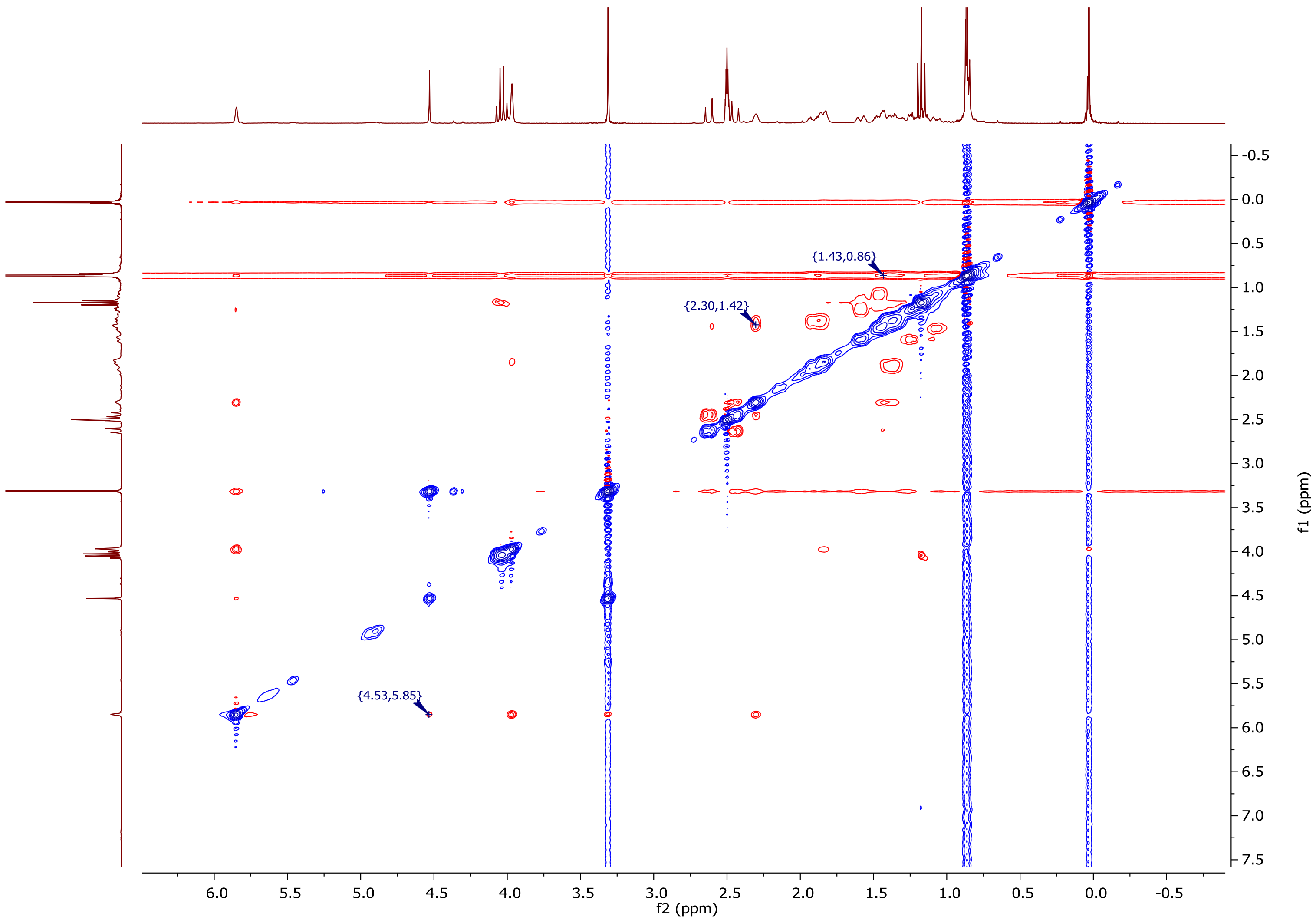

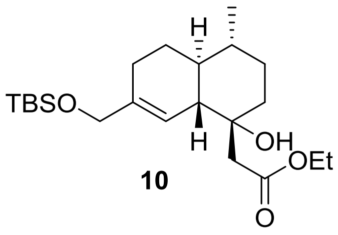

10

(d_6-DMSO, 300 MHz)

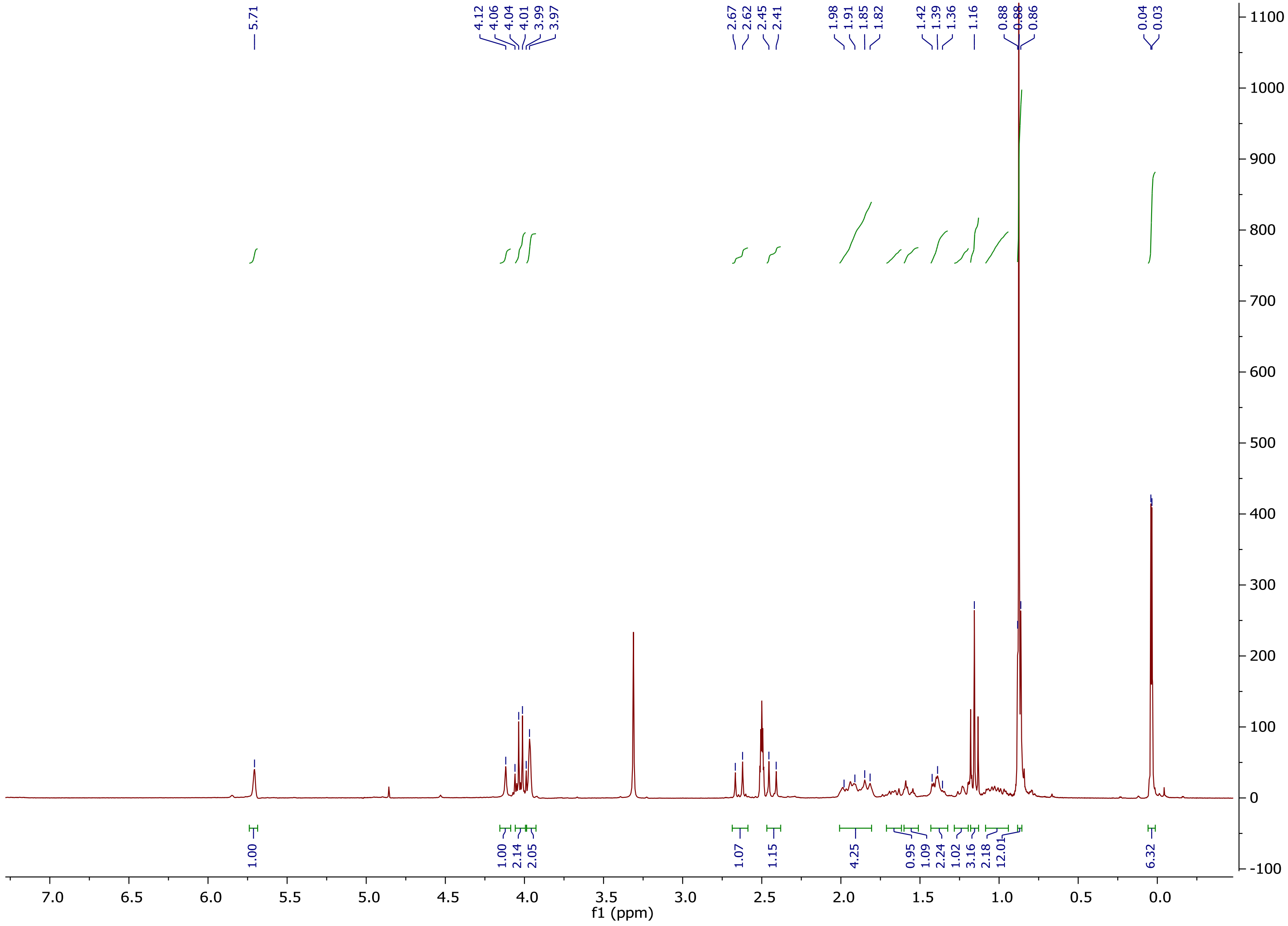

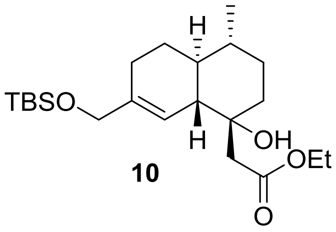

10

(d_6-DMSO, 75 MHz)

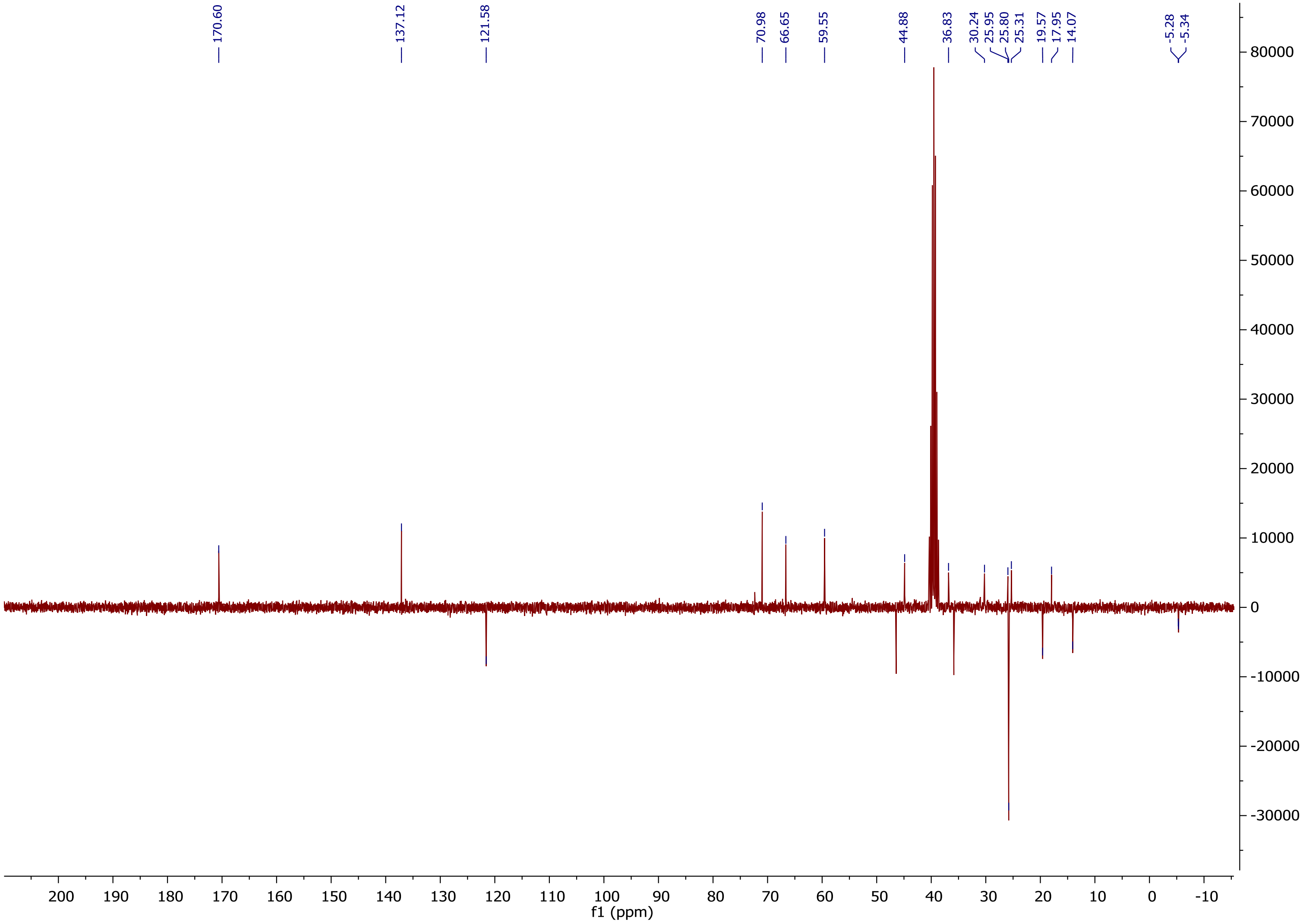

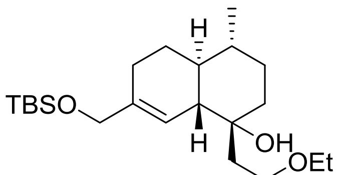

10

(d_6-DMSO, NOESY)

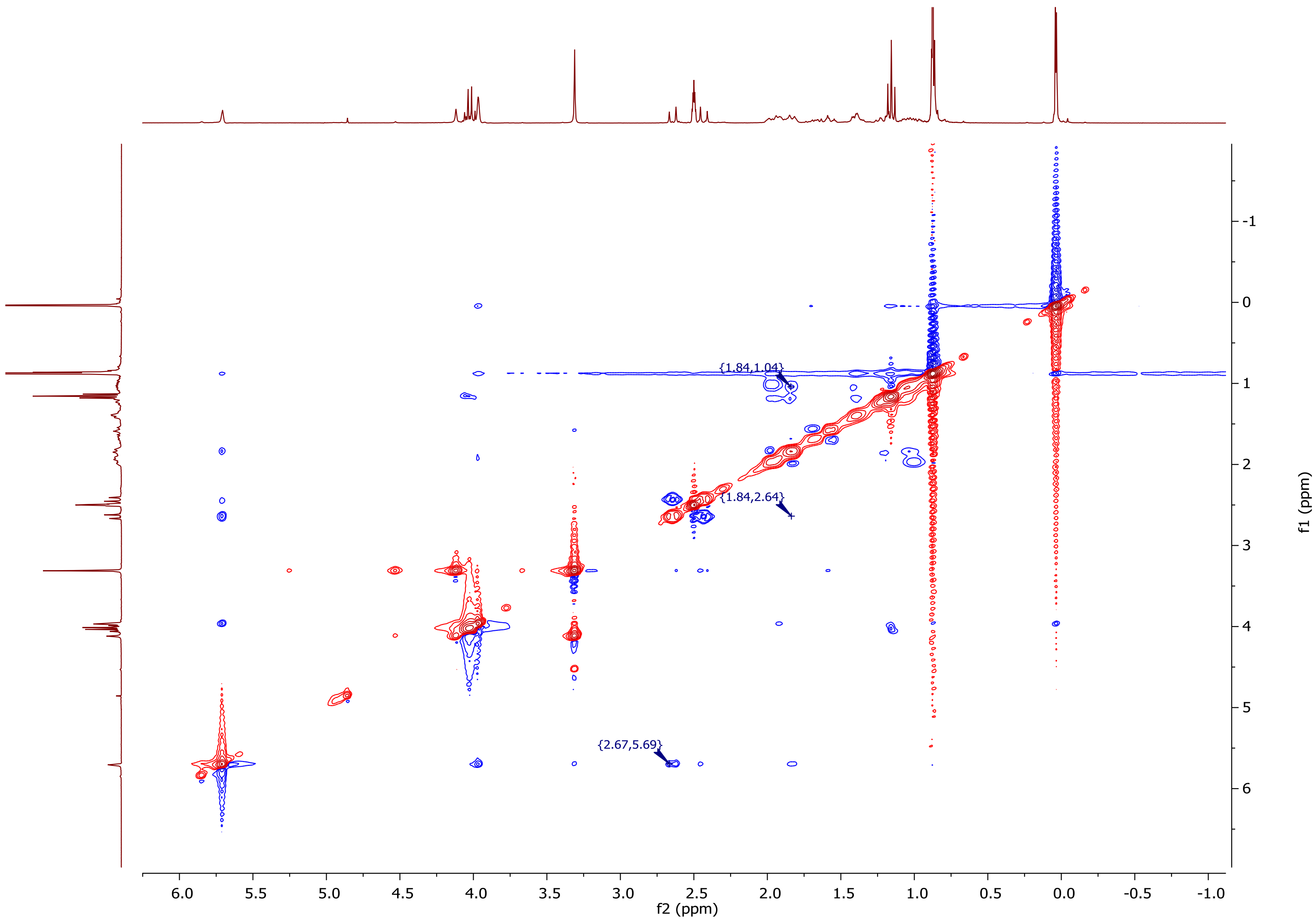

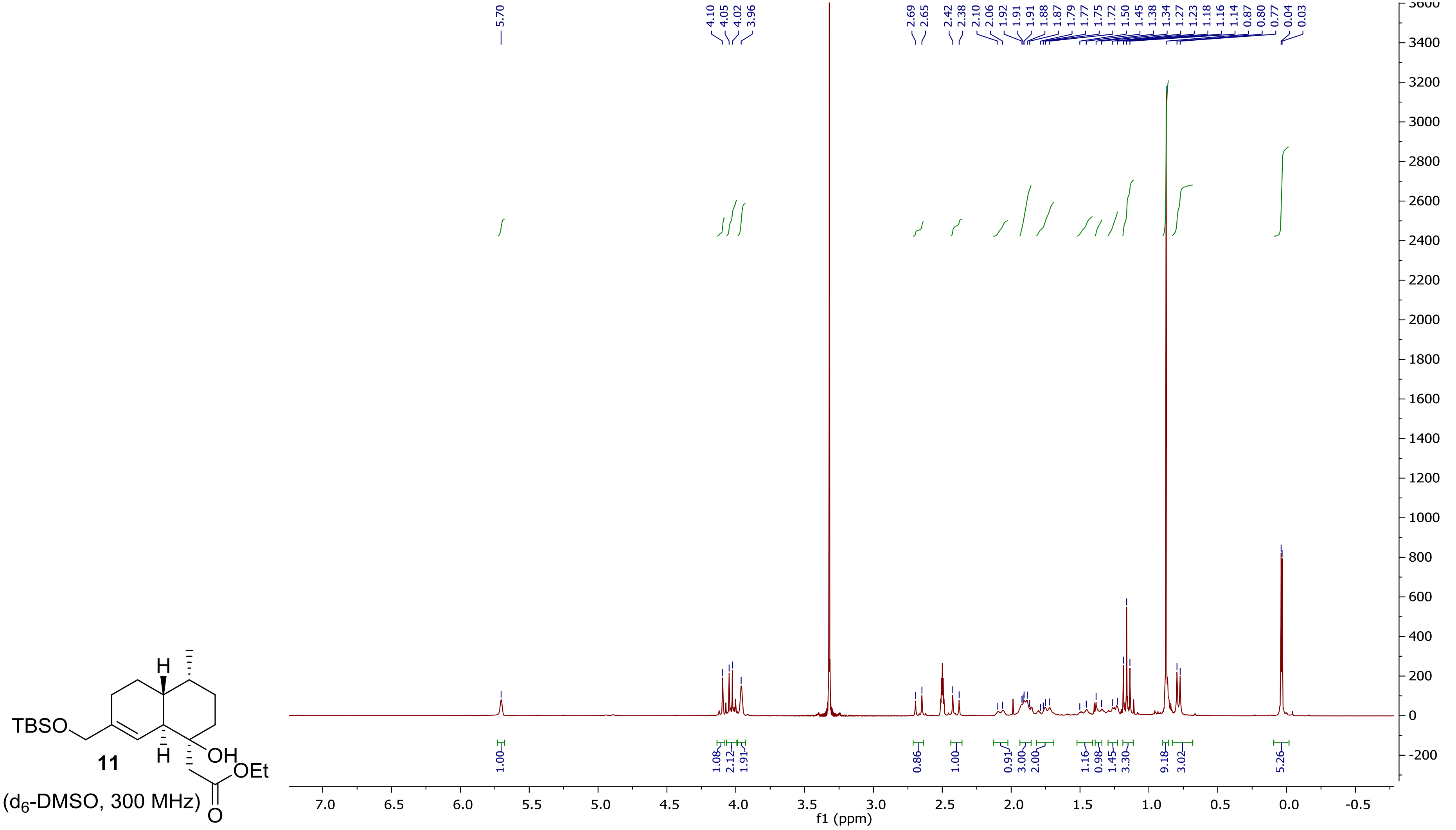

11

(d$_6$-DMSO, 300 MHz)

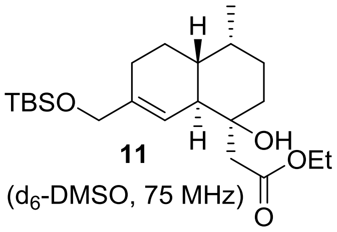

11

(d_6-DMSO, 75 MHz)

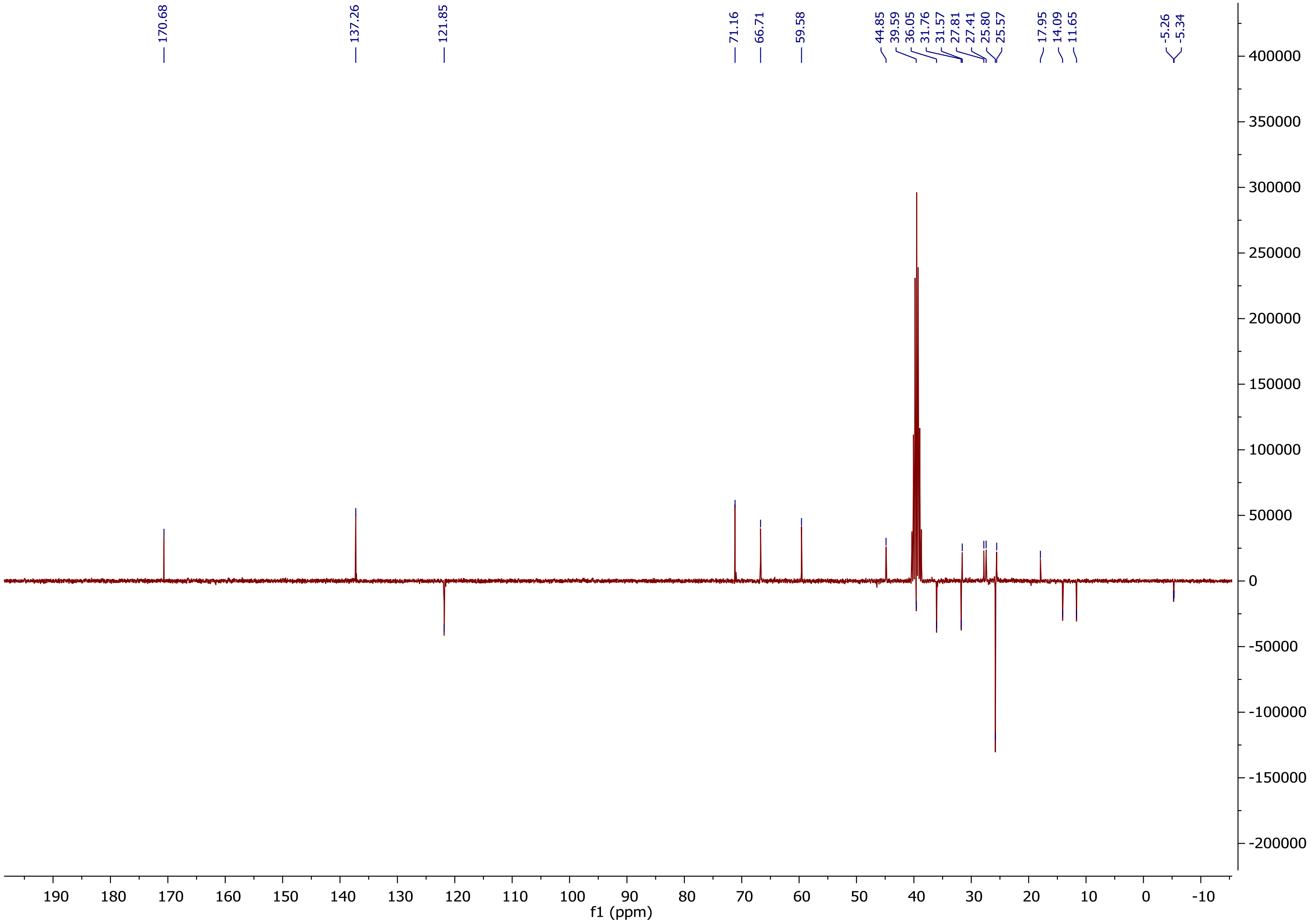

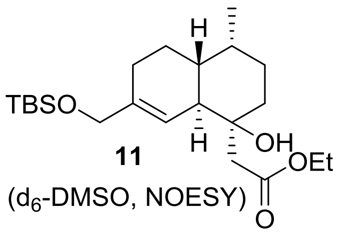

11

(d_6-DMSO, NOESY)

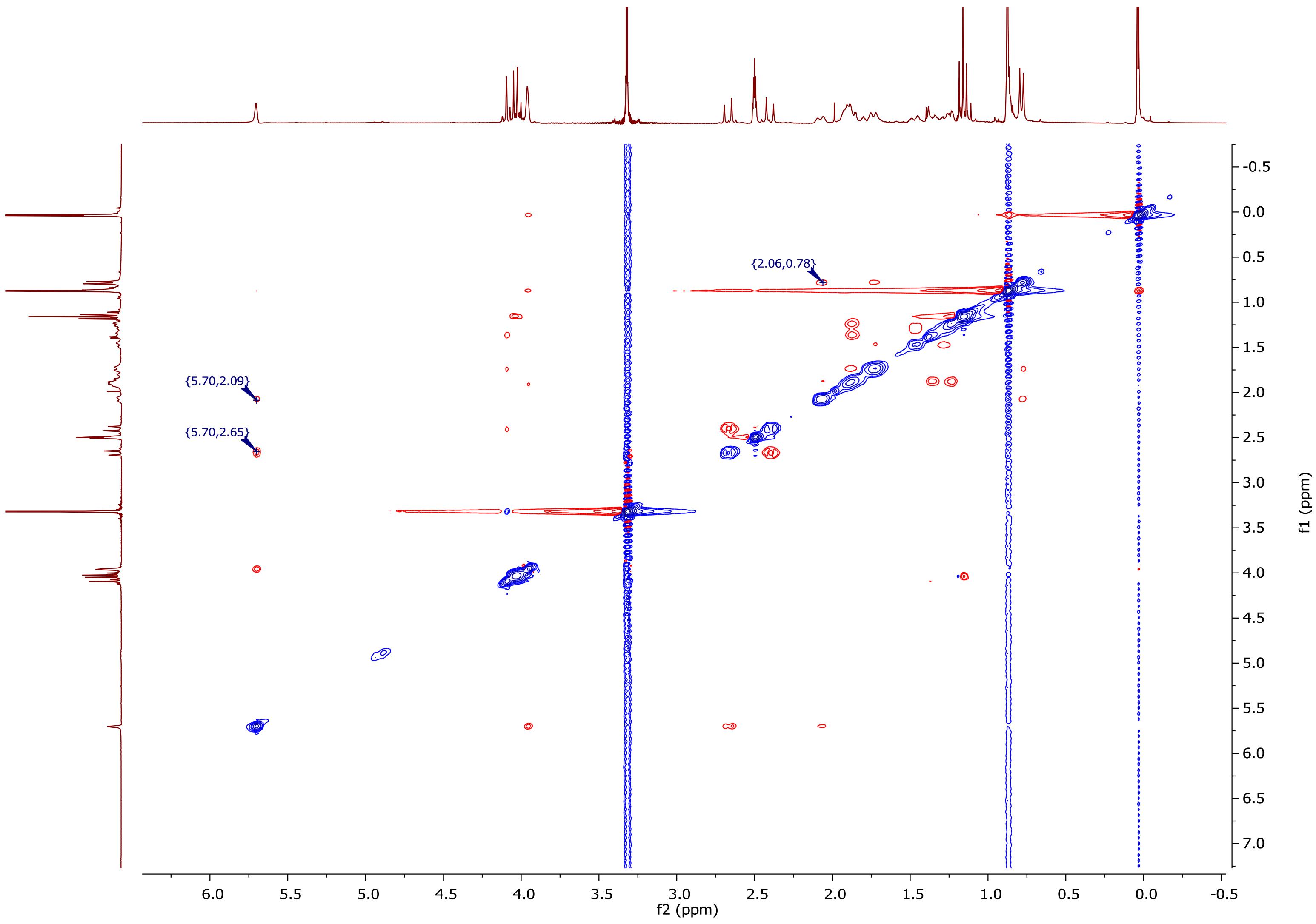

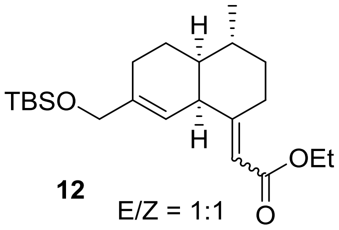

12

E/Z = 1:1

(containing small amounts of other double bond isomers, $CDCl_3$, 400 MHz)

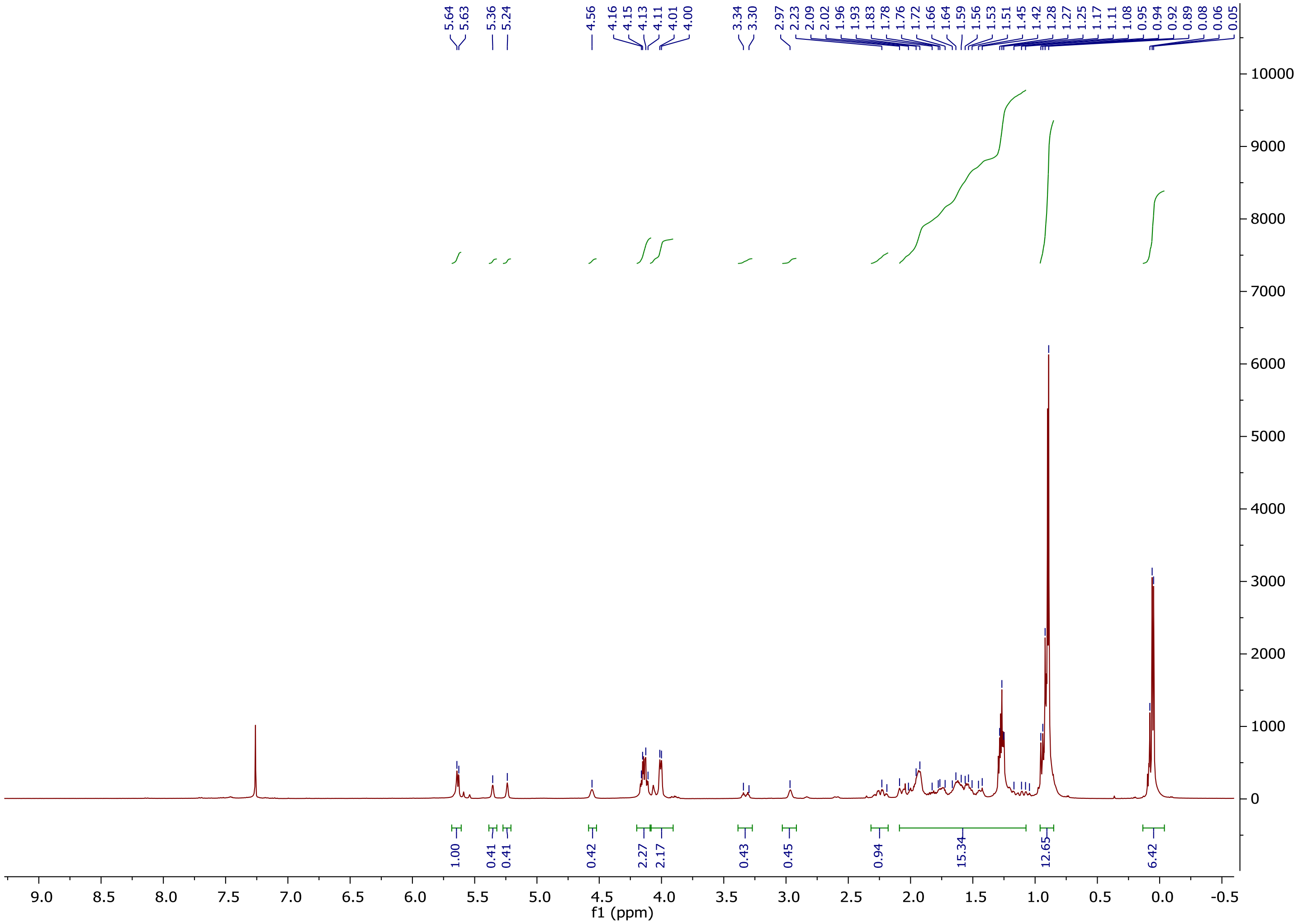

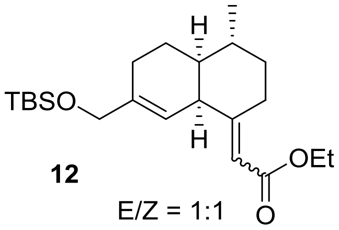

12

E/Z = 1:1

(containing small amounts of other double bond isomers, $CDCl_3$, 75 MHz)

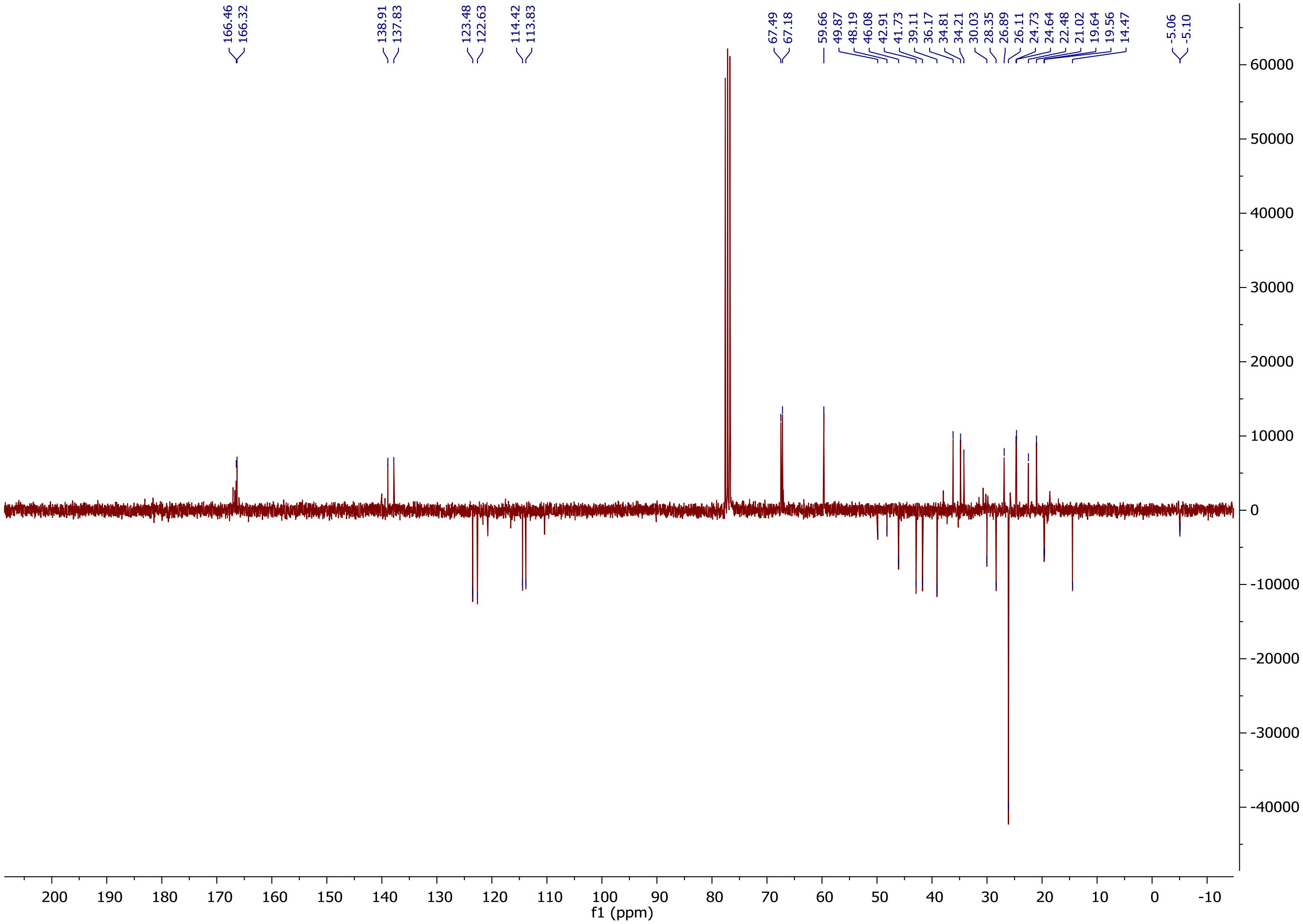

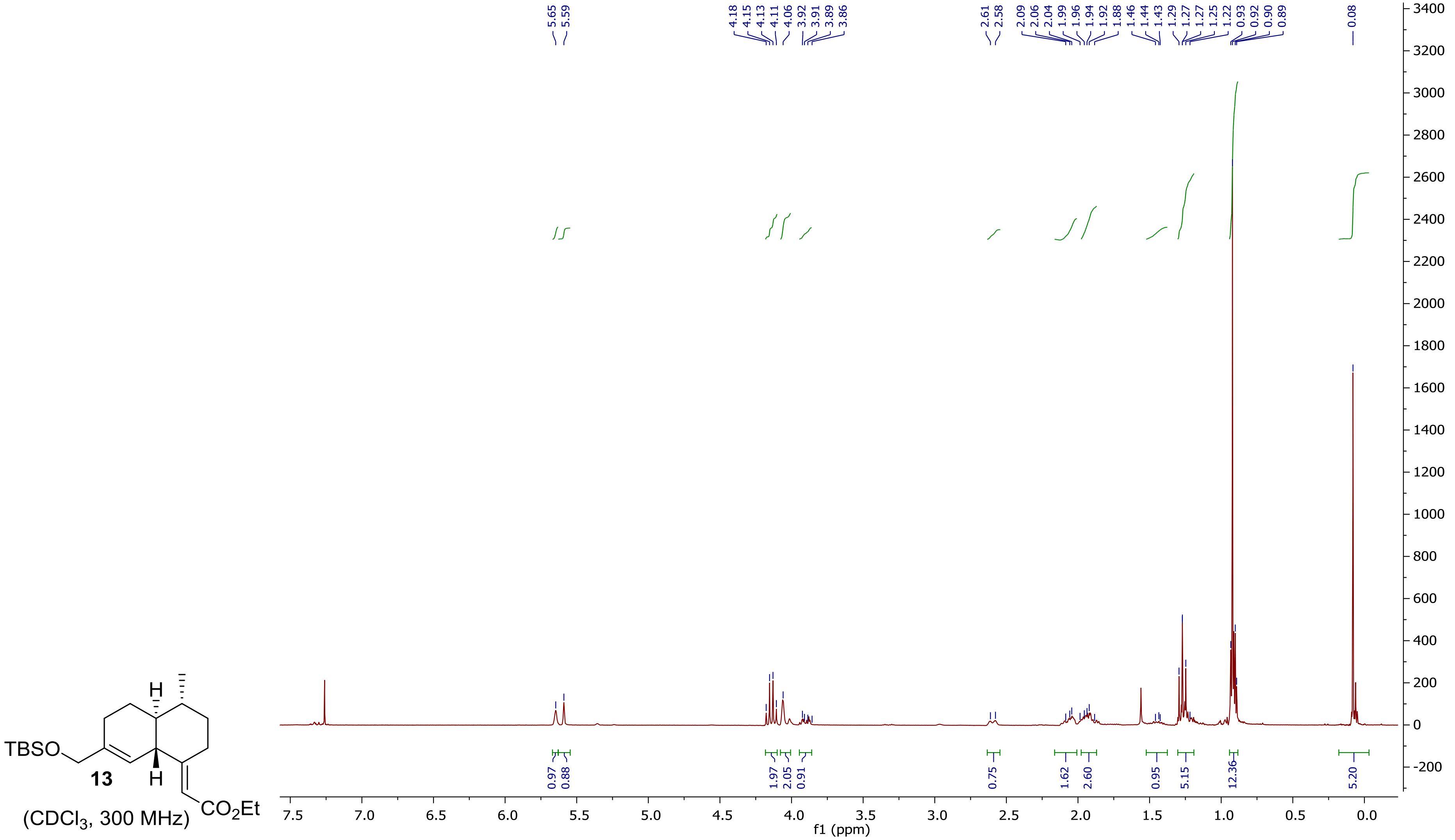

TBSO
13
H
H
CO2Et
(CDCl3, 300 MHz)
5.65 5.59
4.18 4.15 4.13 4.11 4.06 3.92 3.91 3.89 3.86
2.61 2.58 2.09 2.06 2.04 1.99 1.96 1.94 1.92 1.88 1.46 1.44 1.43 1.29 1.27 1.27 1.25 1.22 0.93 0.92 0.90 0.89
0.08
0.97 0.88 1.97 2.05 0.91 0.75 1.62 2.60 0.95 5.15 12.36 5.20
f1 (ppm)

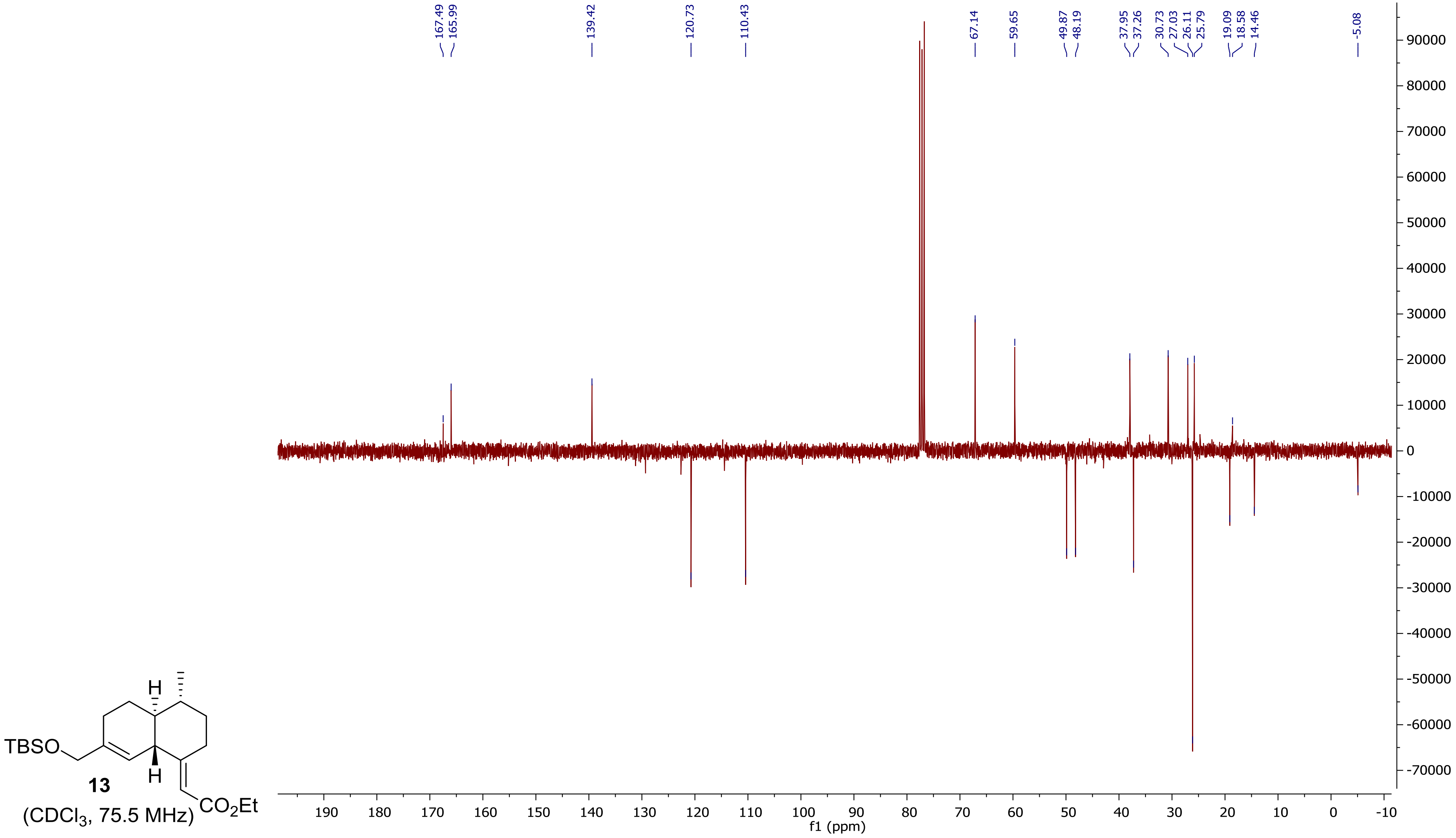

13

($CDCl_3$, 75.5 MHz)

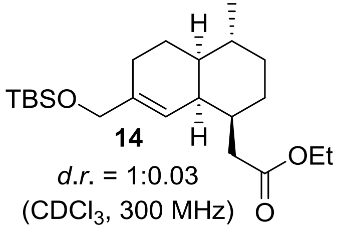

14

d.r. = 1:0.03

($CDCl_3$, 300 MHz)

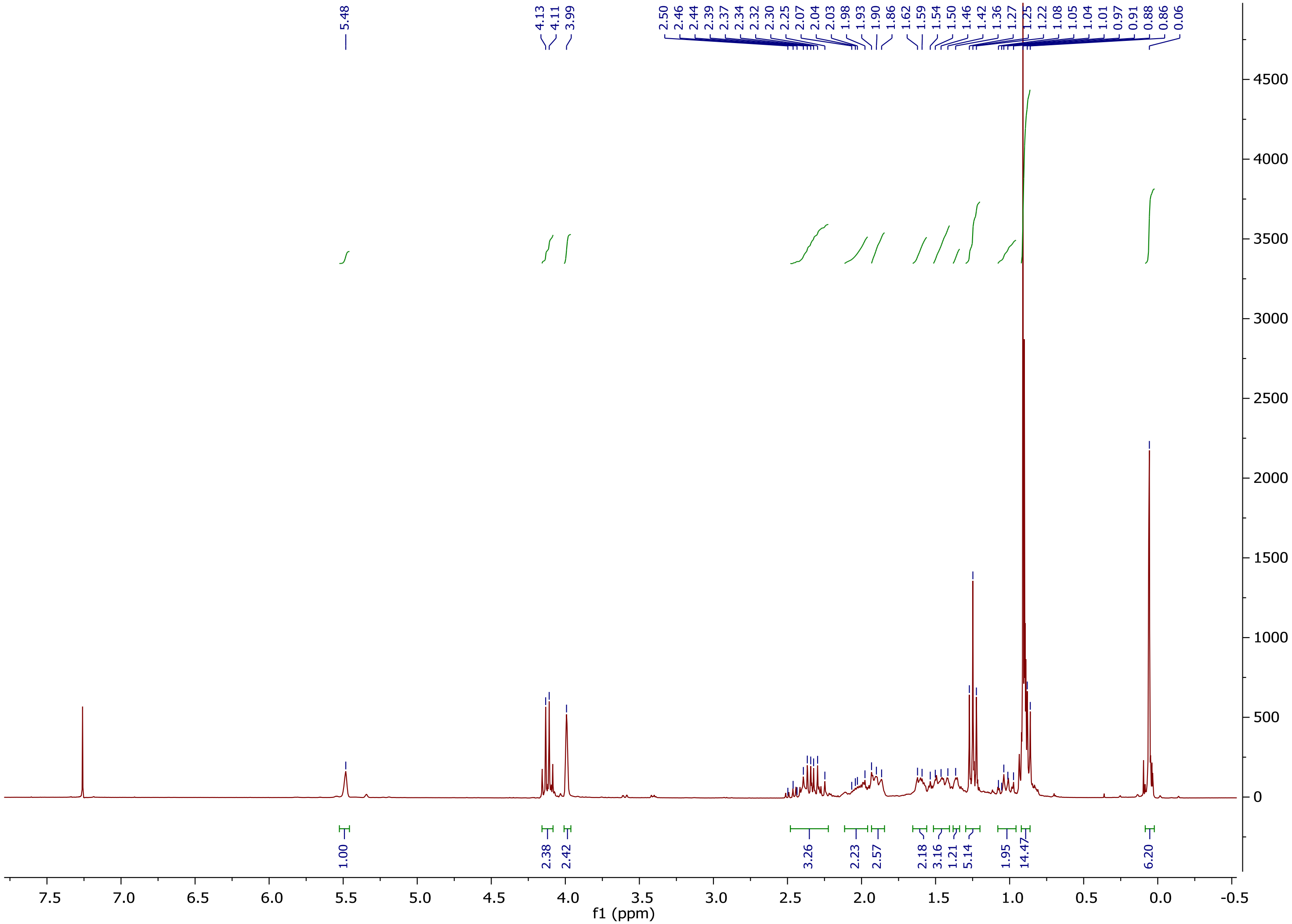

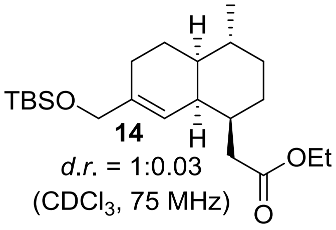

14

d.r. = 1:0.03

($CDCl_3$, 75 MHz)

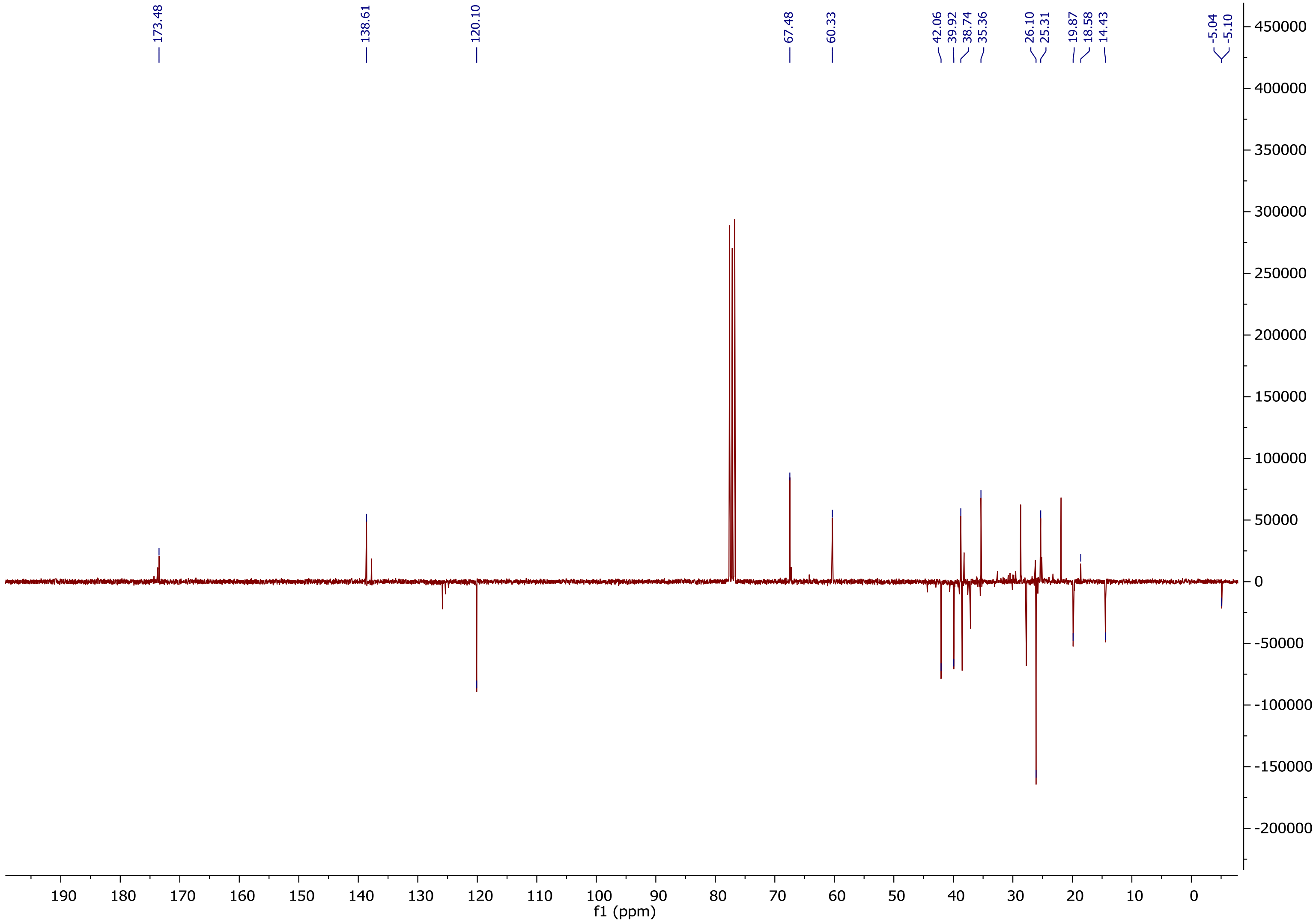

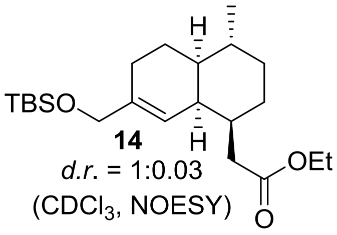

14
d.r. = 1:0.03
($CDCl_3$, NOESY)

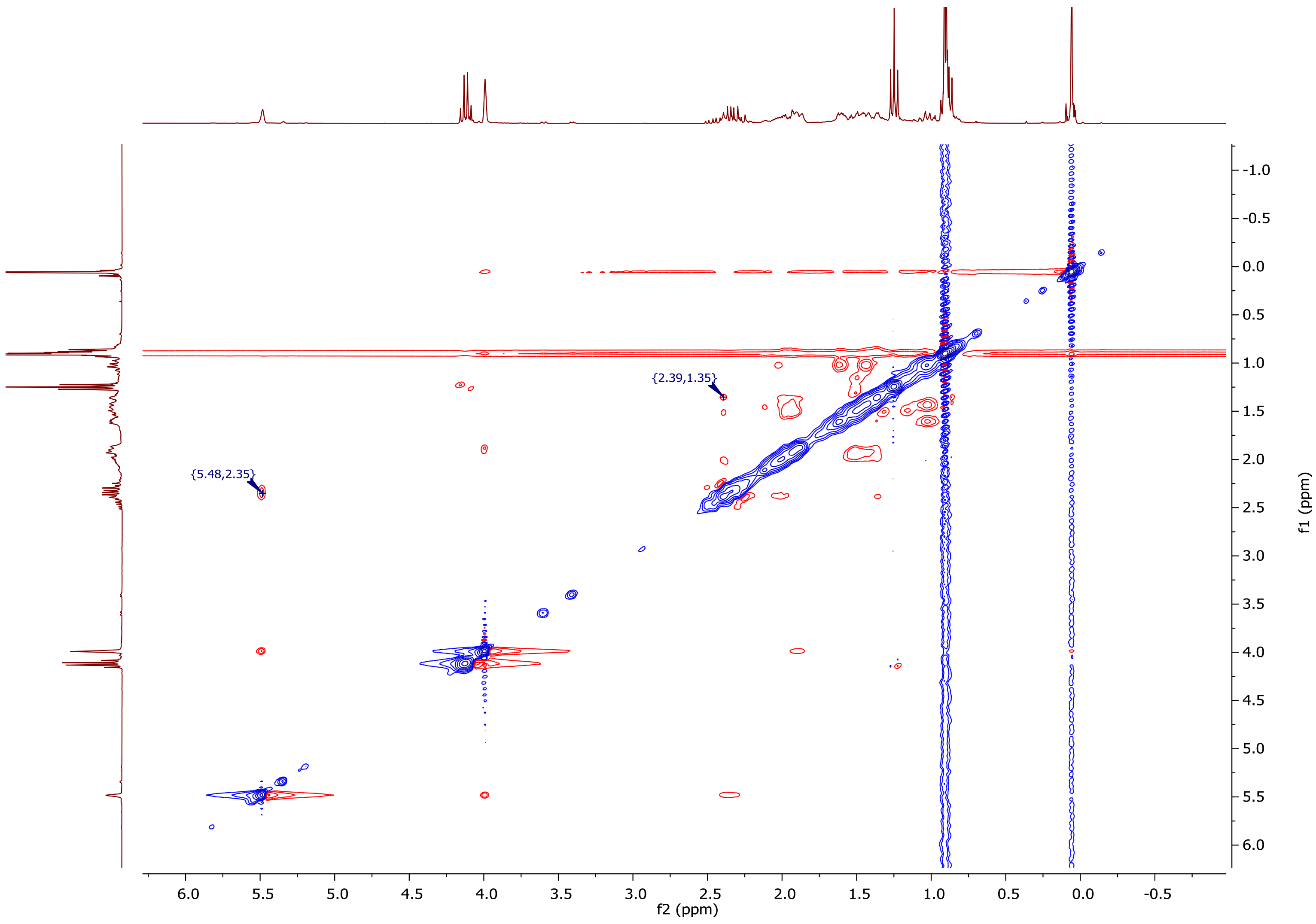

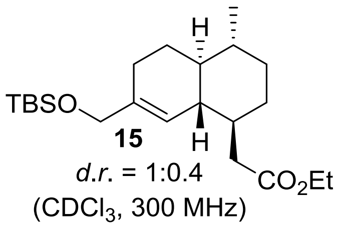

15

d.r. = 1:0.4

($CDCl_3$, 300 MHz)

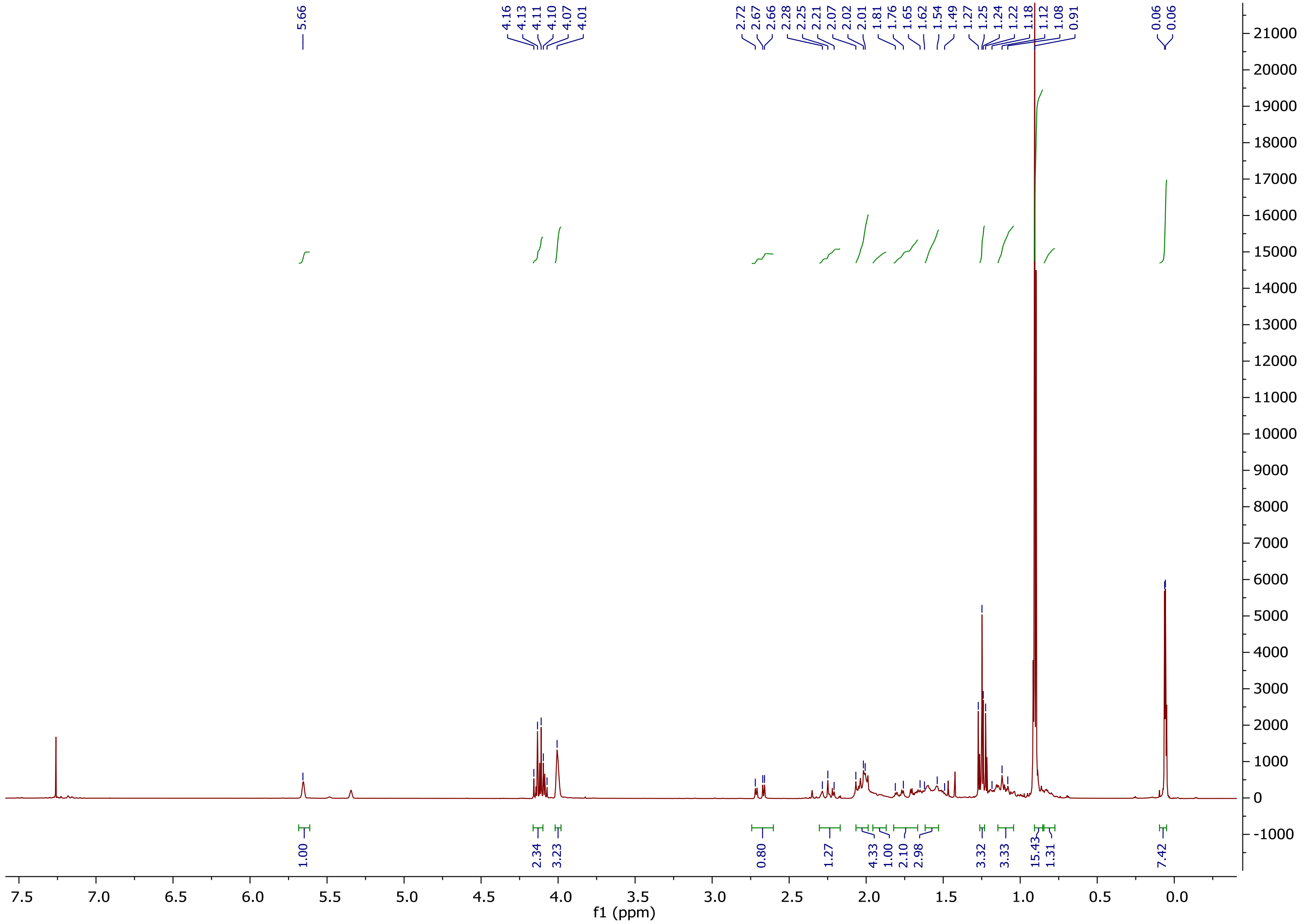

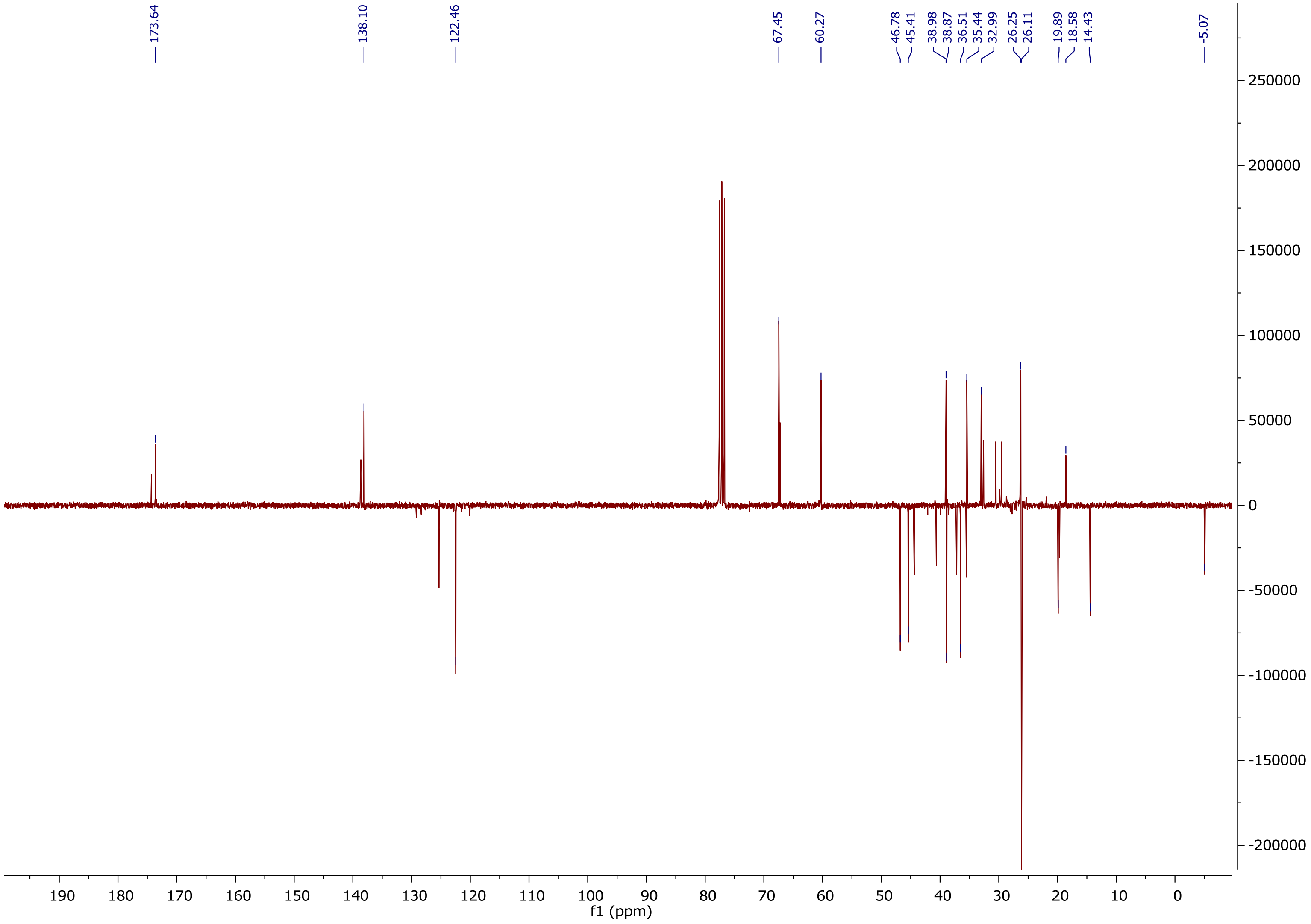

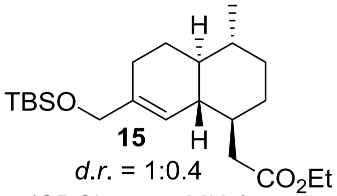

15

d.r. = 1:0.4

($CDCl_3$, 75.5 MHz)

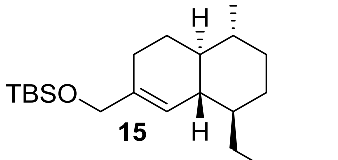

15

(CDCl$_3$, NOESY)

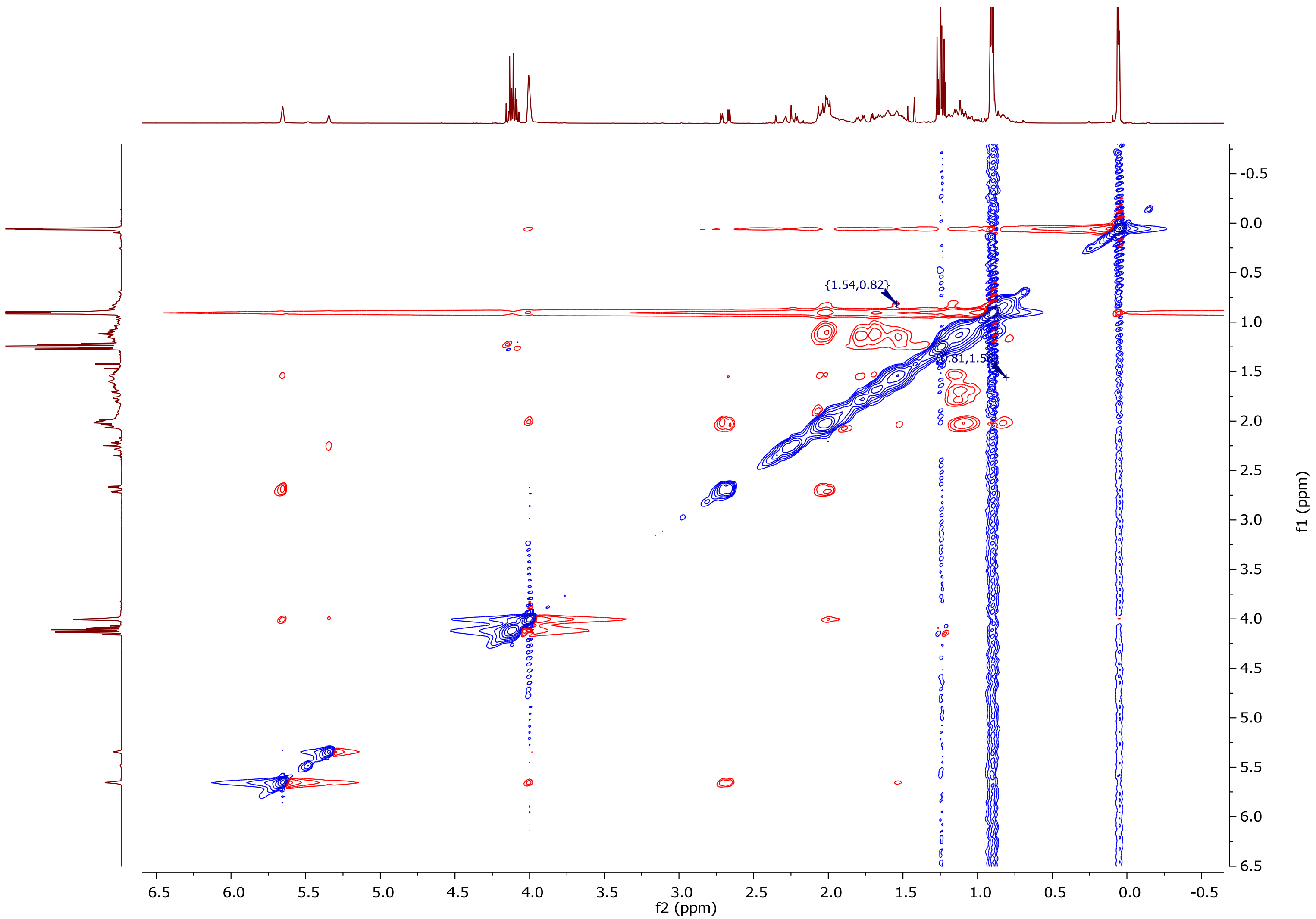

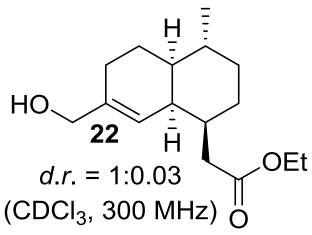

22

d.r. = 1:0.03

($CDCl_3$, 300 MHz)

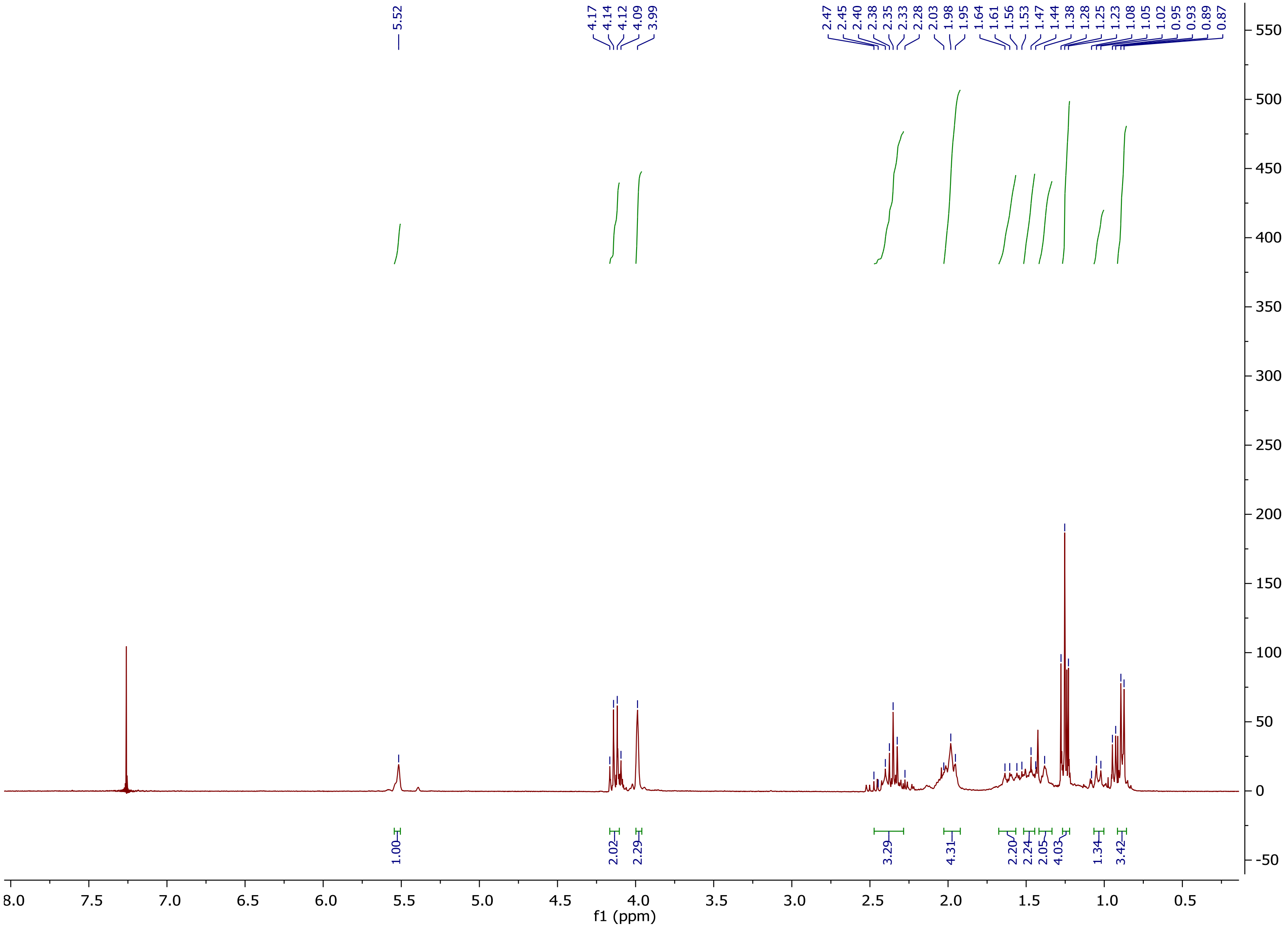

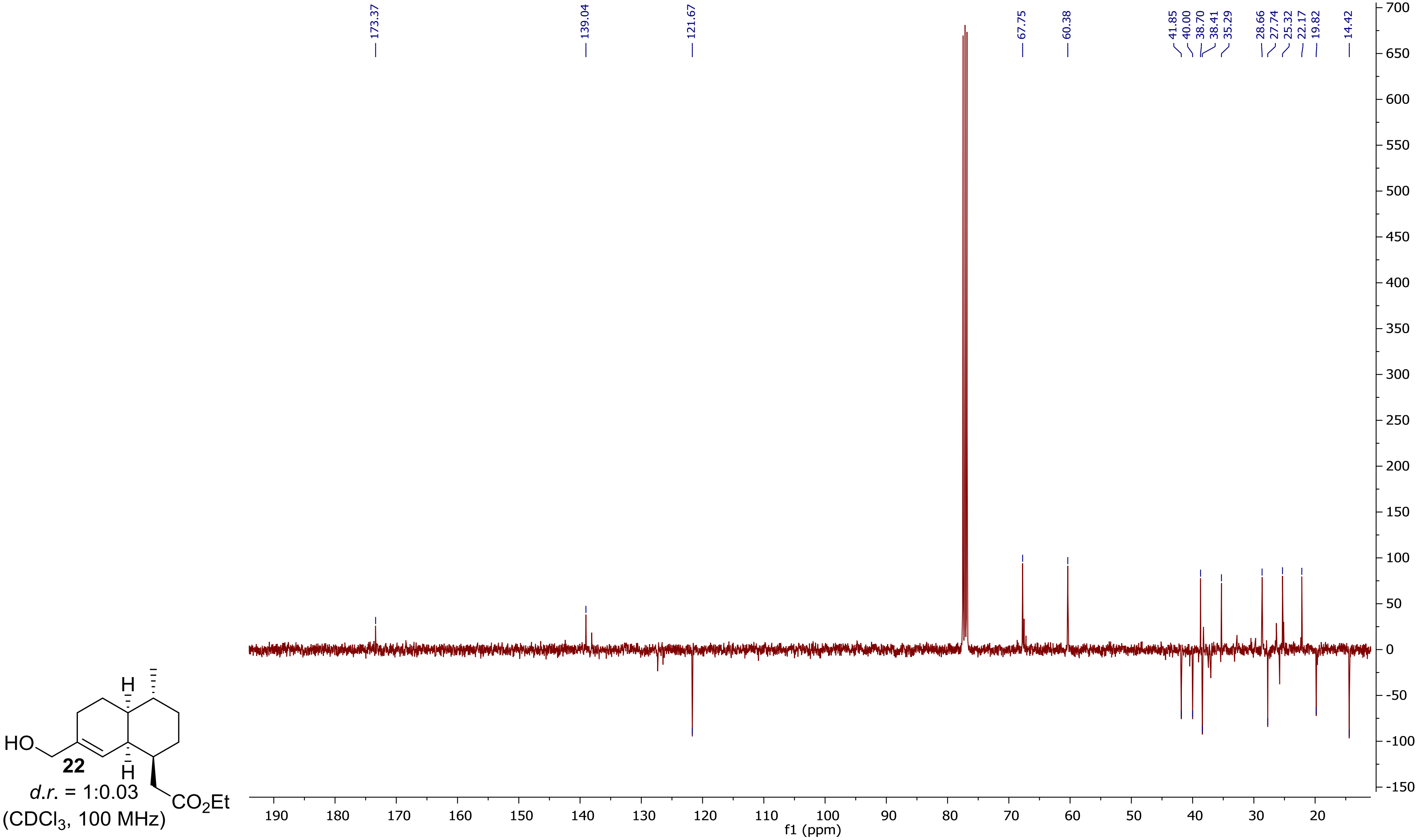

22

d.r. = 1:0.03

($CDCl_3$, 100 MHz)

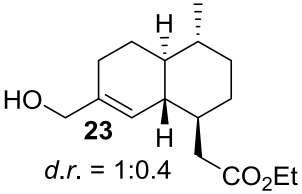

23

d.r. = 1:0.4

($CDCl_3$, 400 MHz)

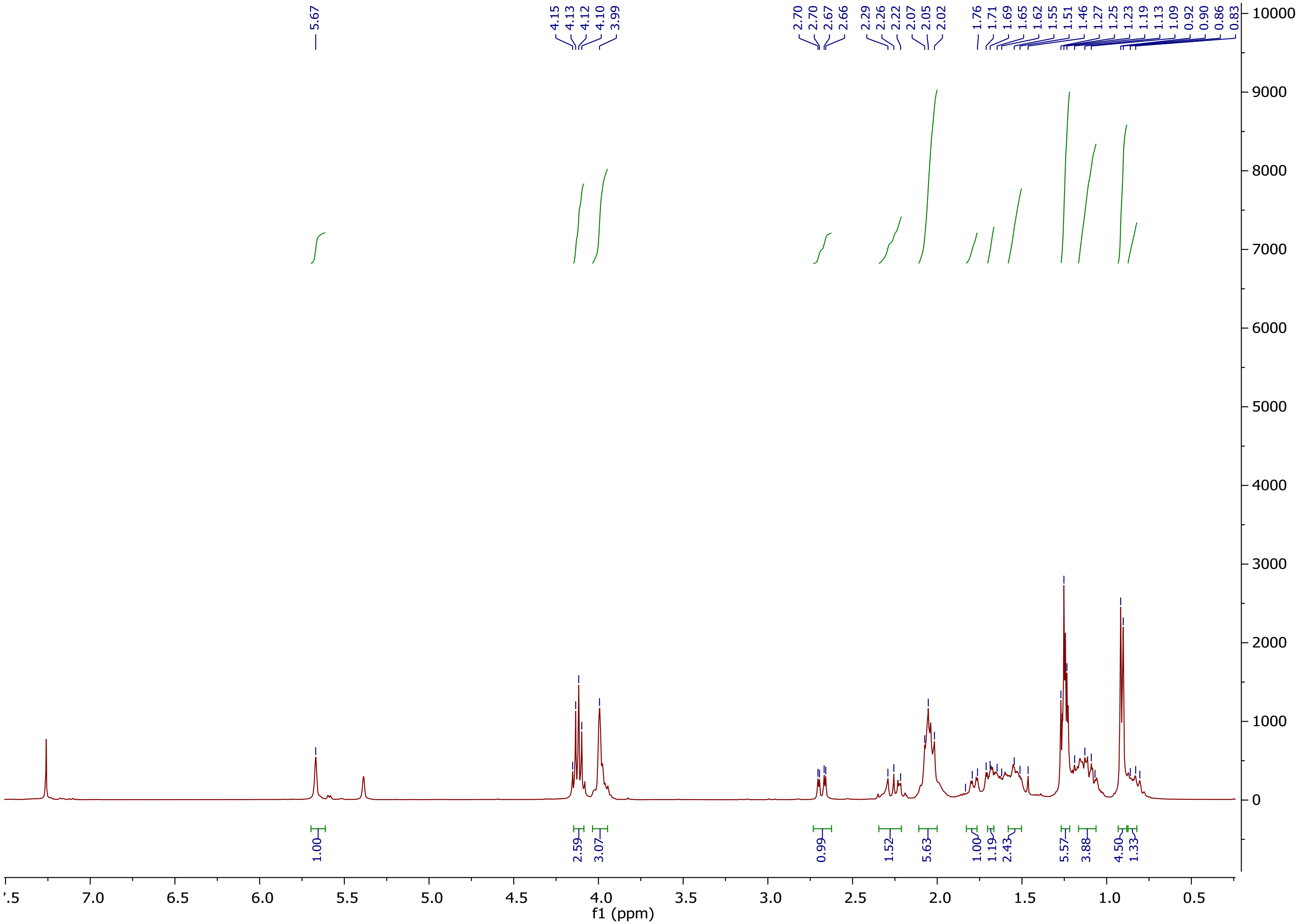

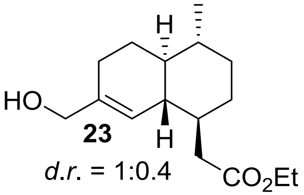

23

d.r. = 1:0.4

($CDCl_3$, 100 MHz)

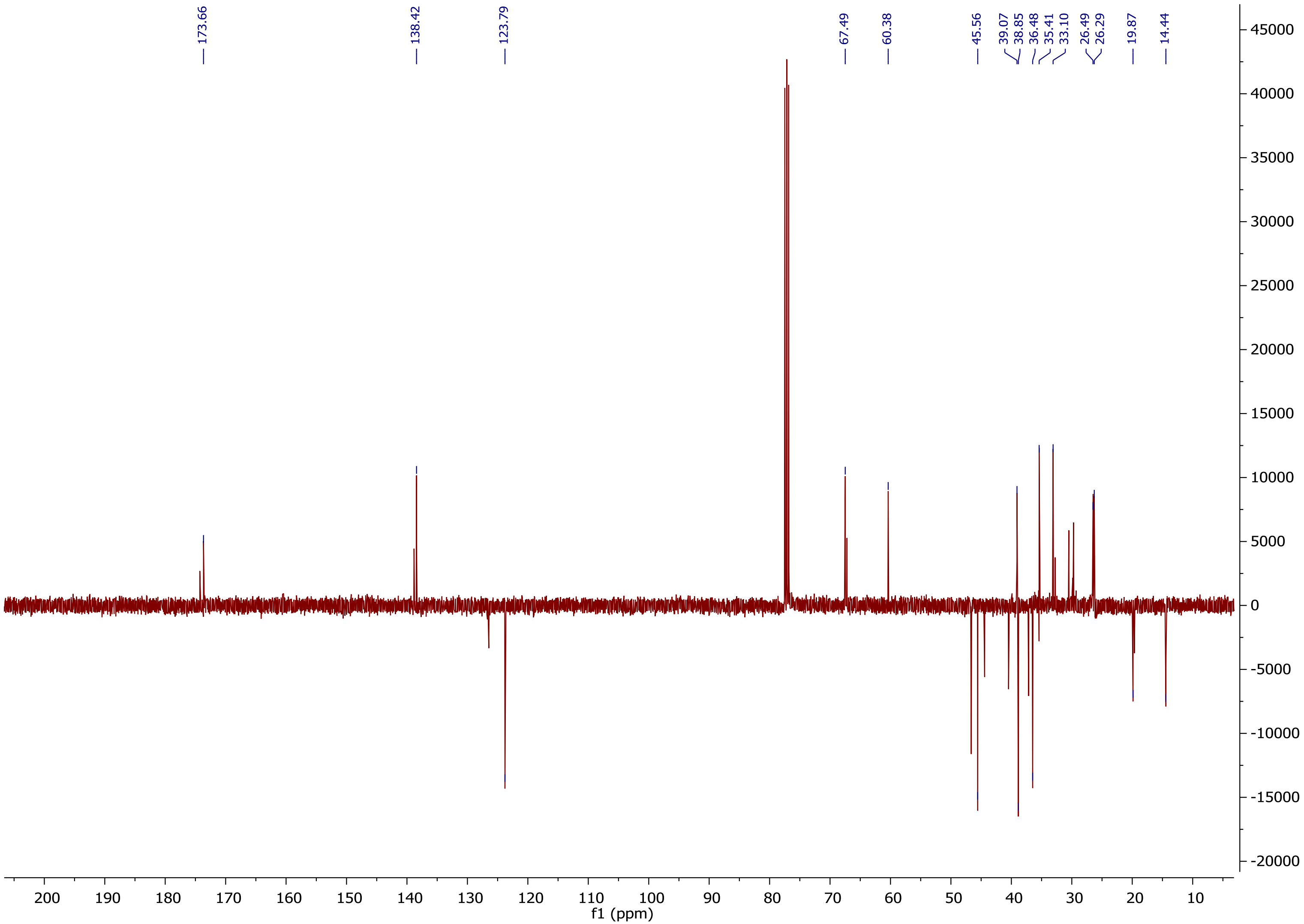

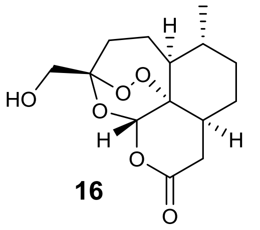

16

($CDCl_3$, 400 MHz)

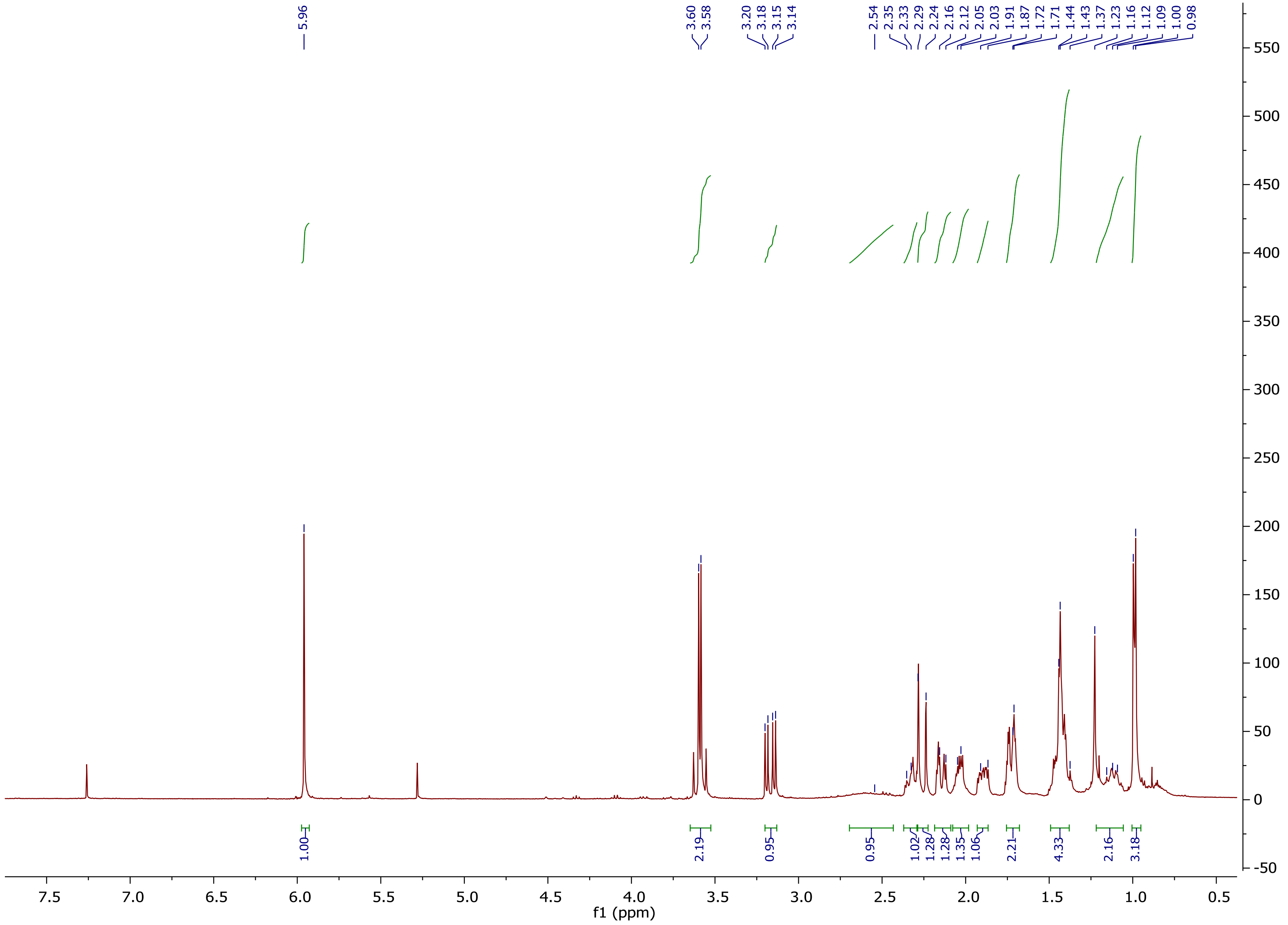

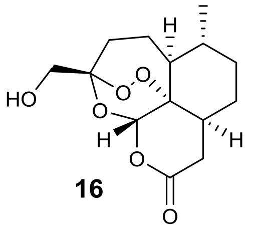

16

($CDCl_3$, 100 MHz)

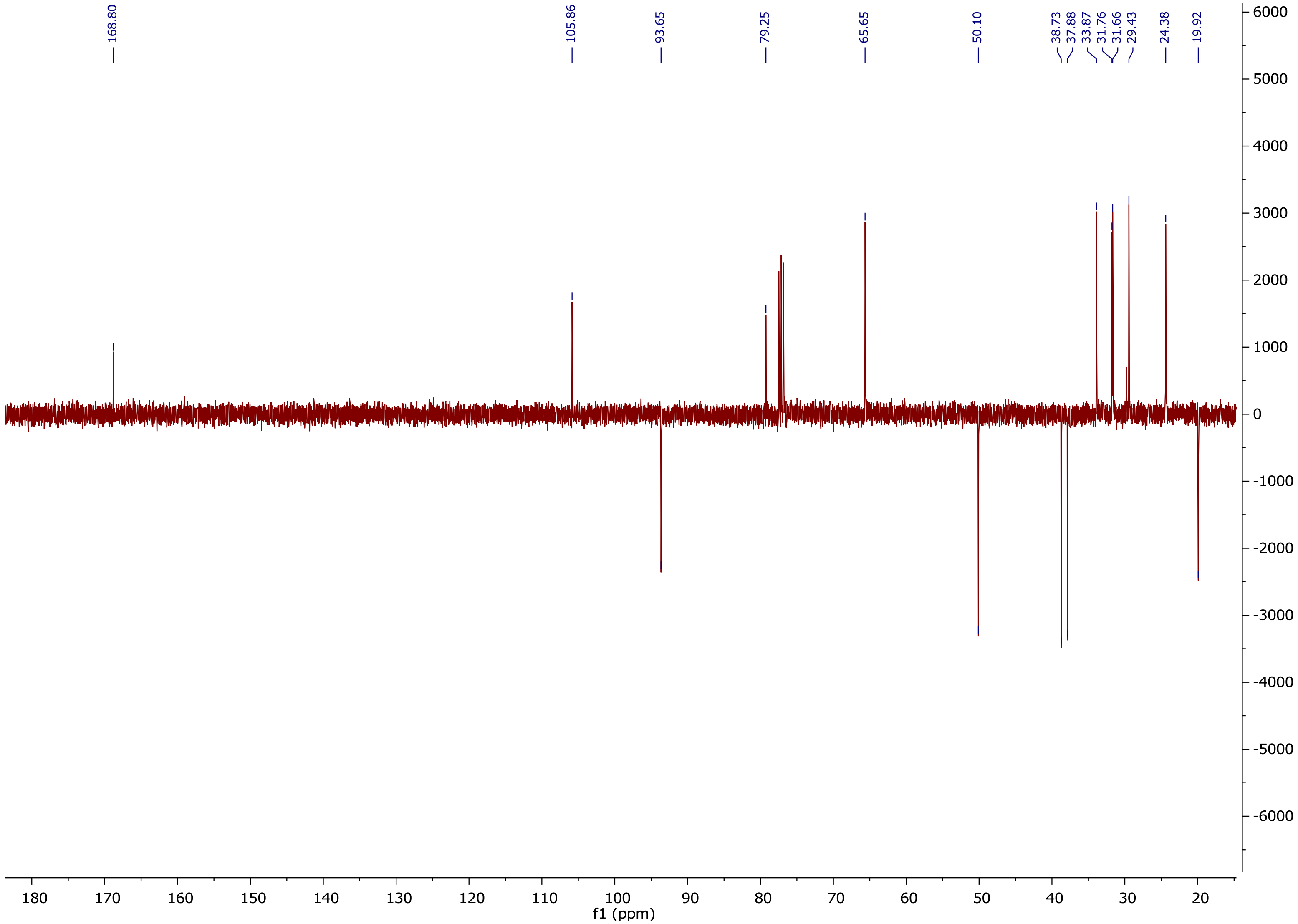

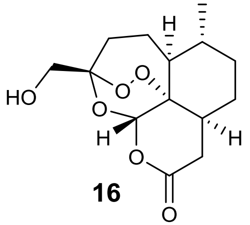

16

(CDCl$_3$, NOESY)

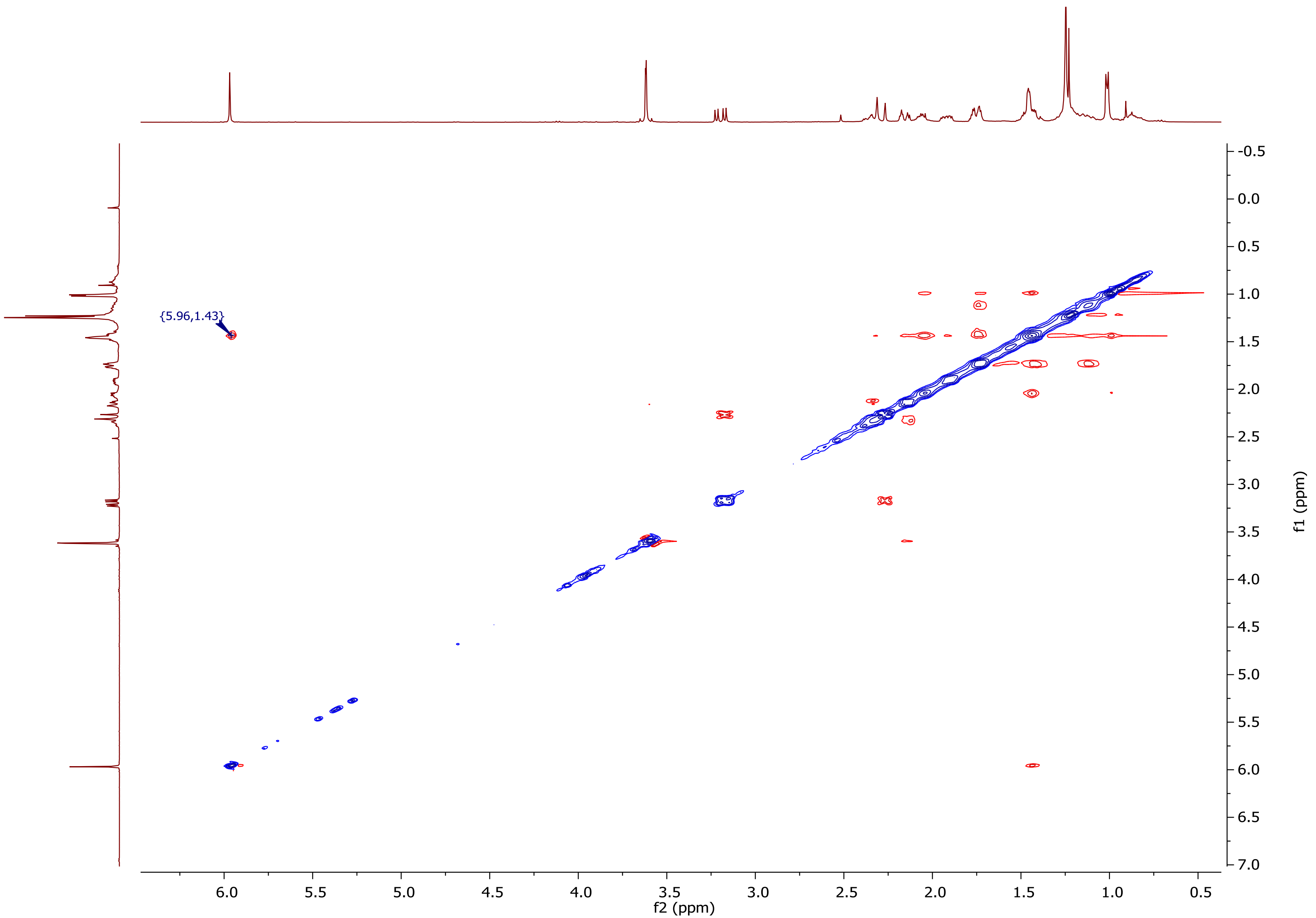

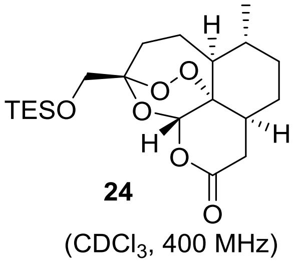

24

($CDCl_3$, 400 MHz)

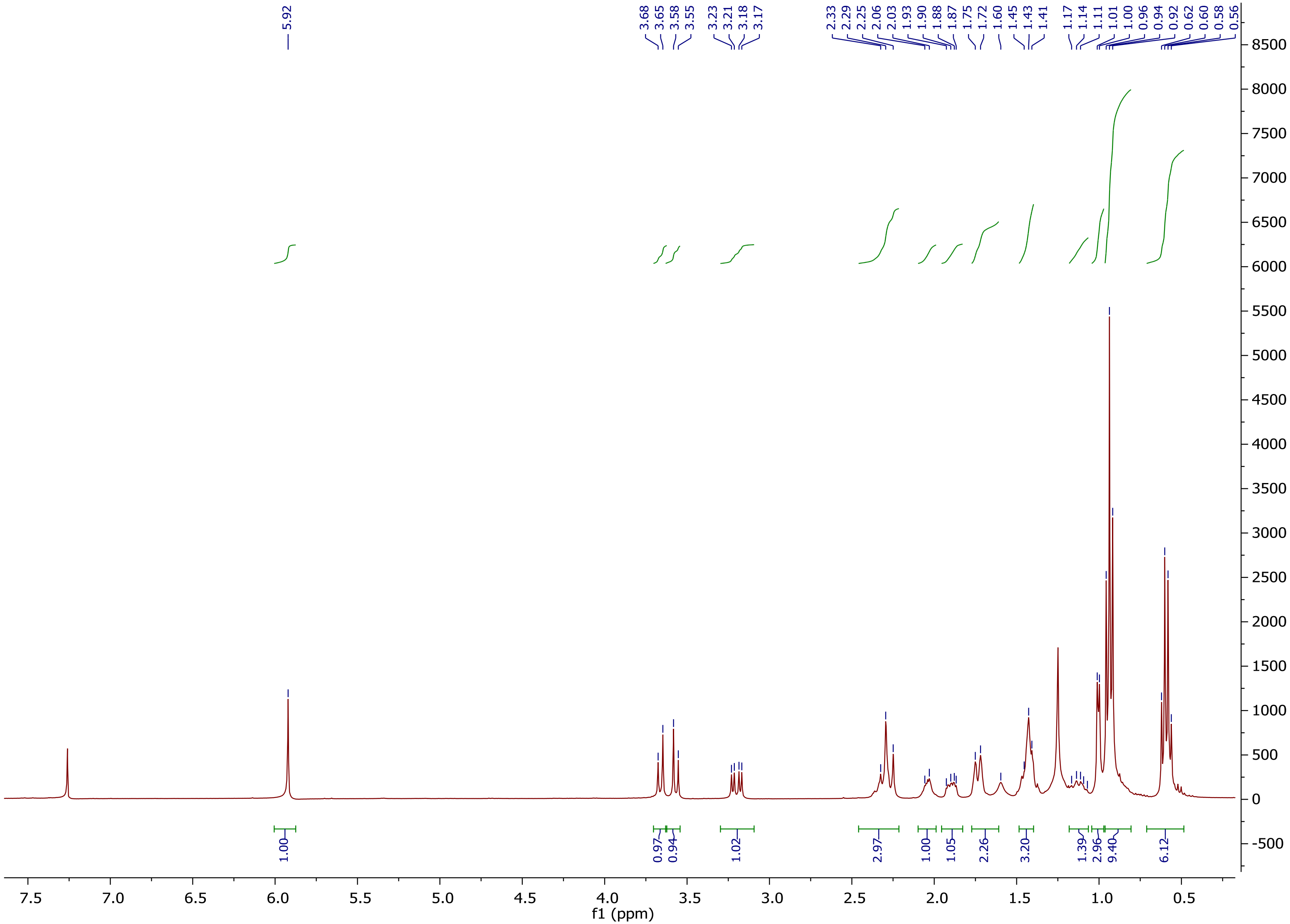

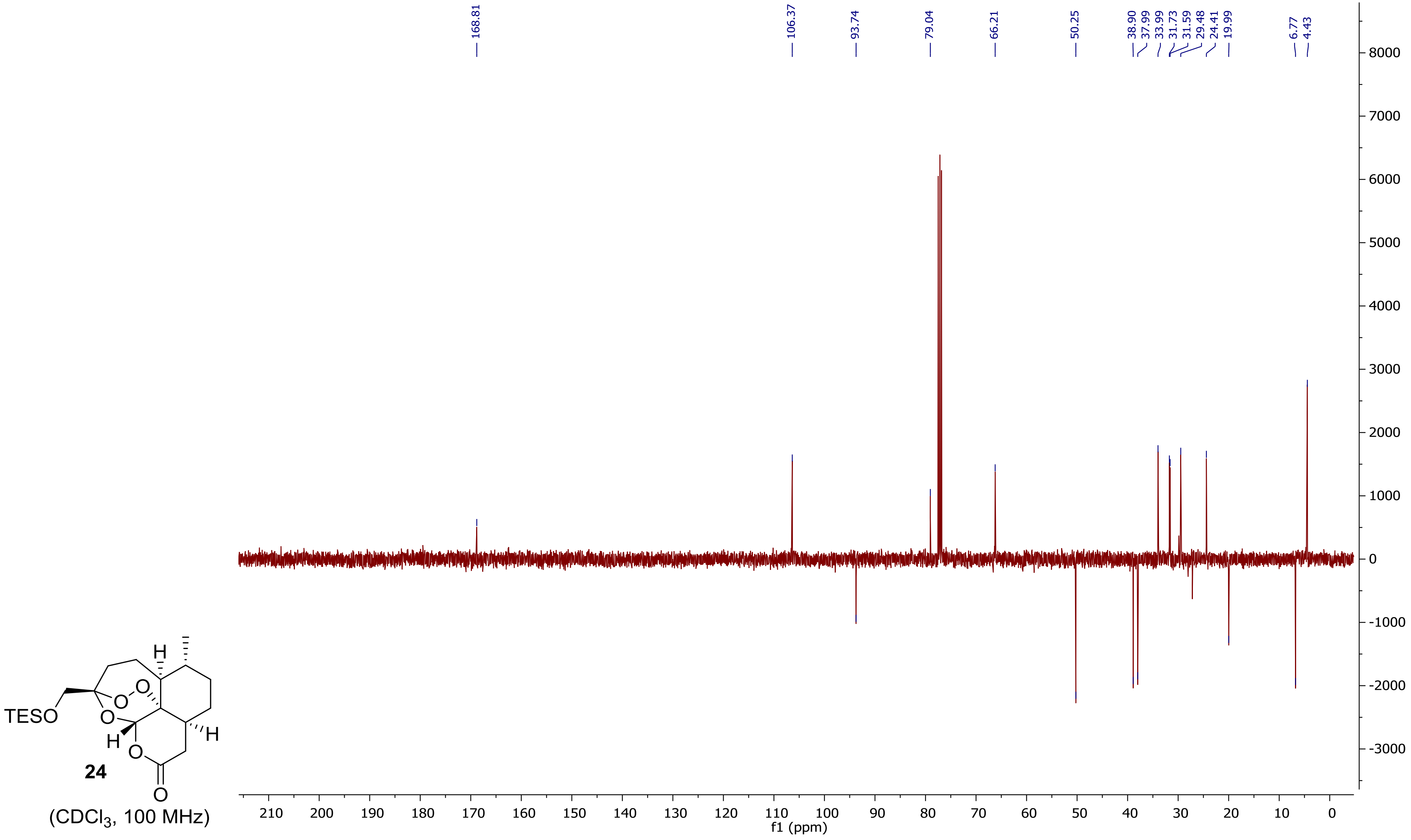
TESO
H
O
24
(CDCl3, 100 MHz)
168.81
106.37
93.74
79.04
66.21
50.25
38.90
37.99
33.99
31.73
31.59
29.48
24.41
19.99
6.77
4.43
f1 (ppm)

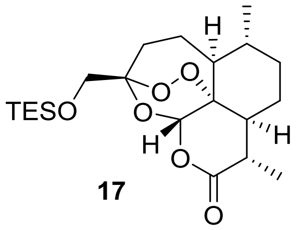

17

($CDCl_3$, 400 MHz)

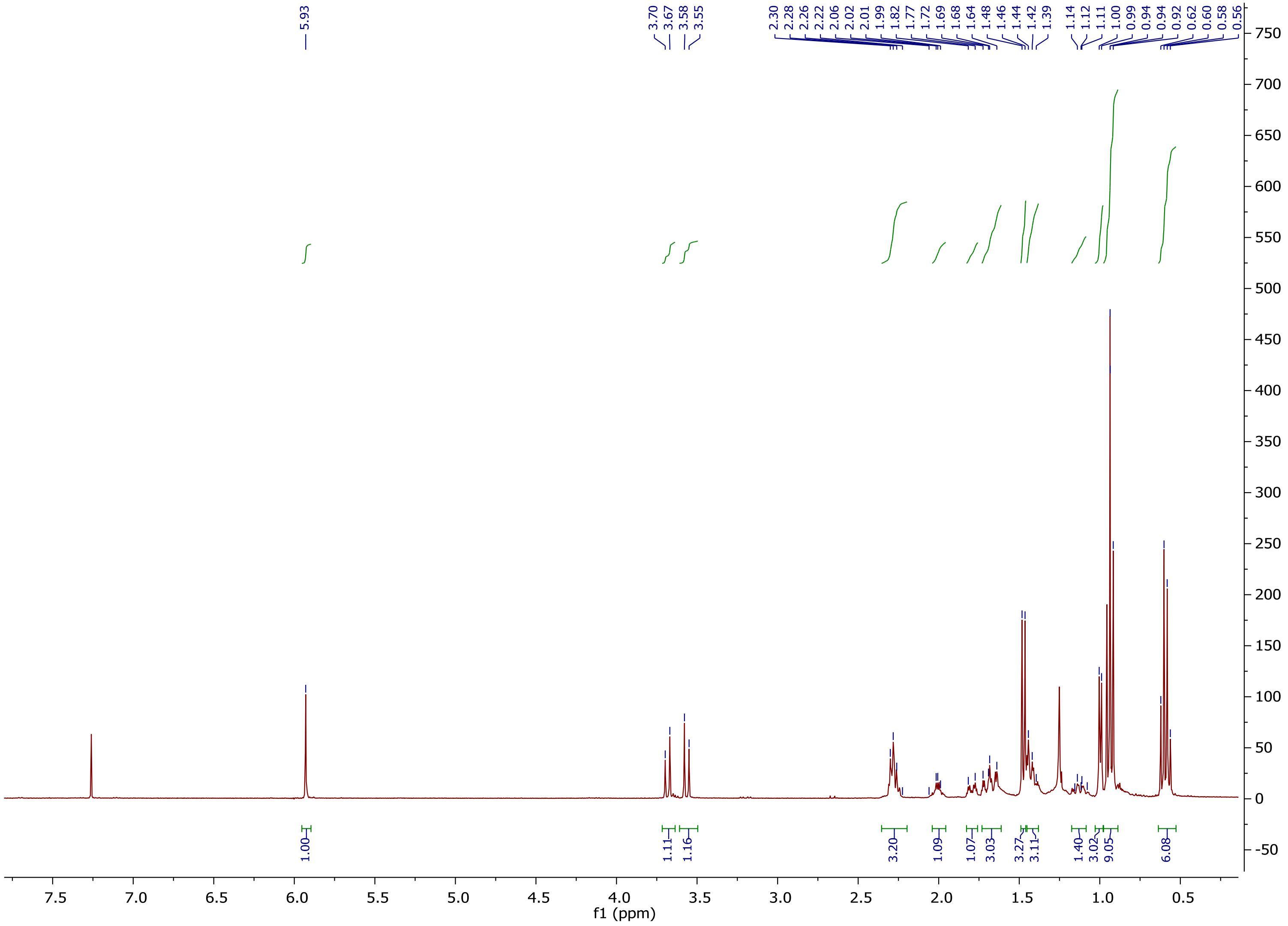

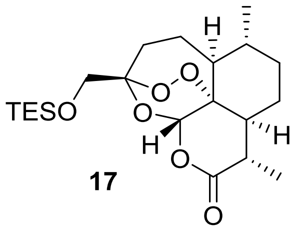

17

($CDCl_3$, 100 MHz)

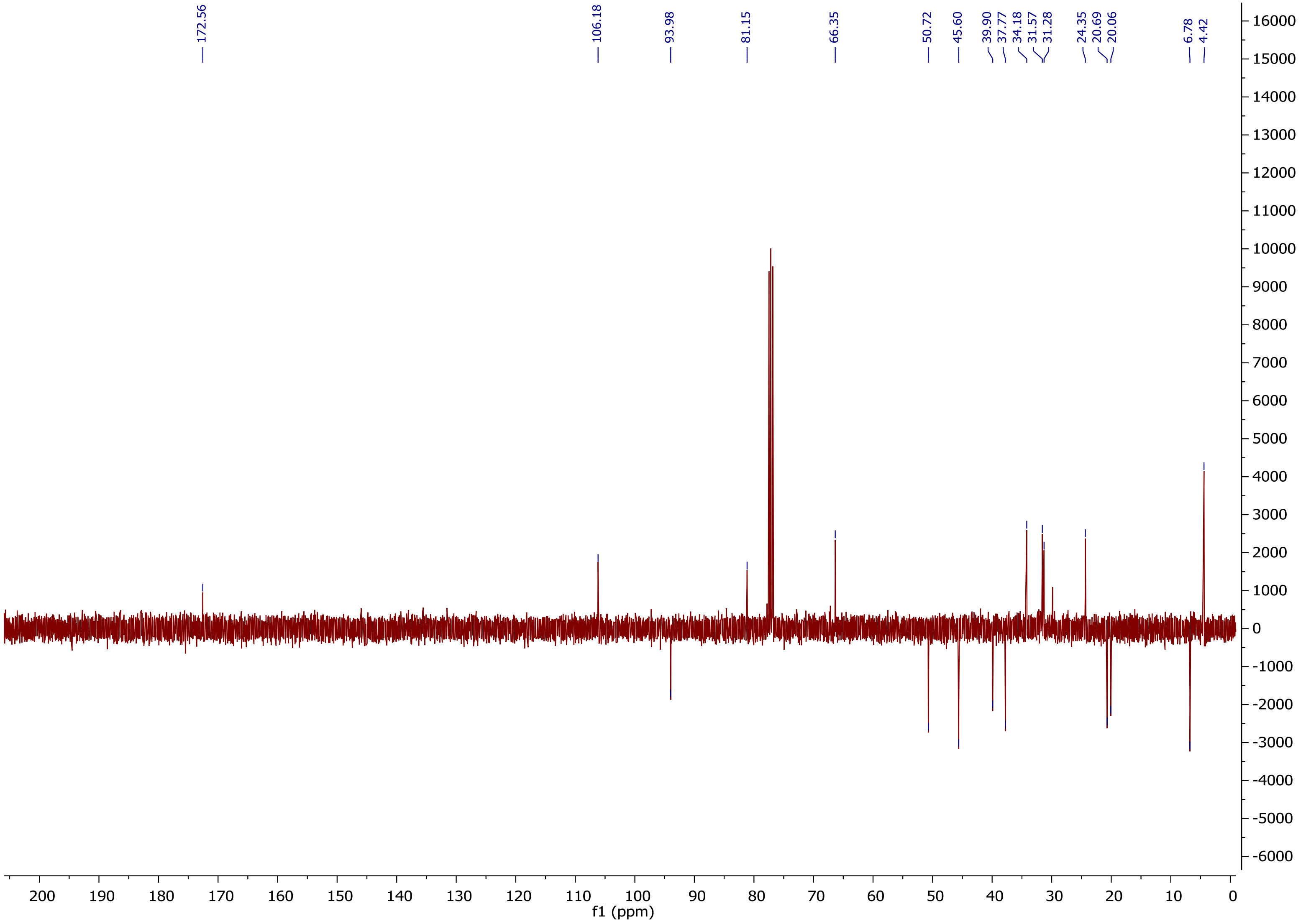

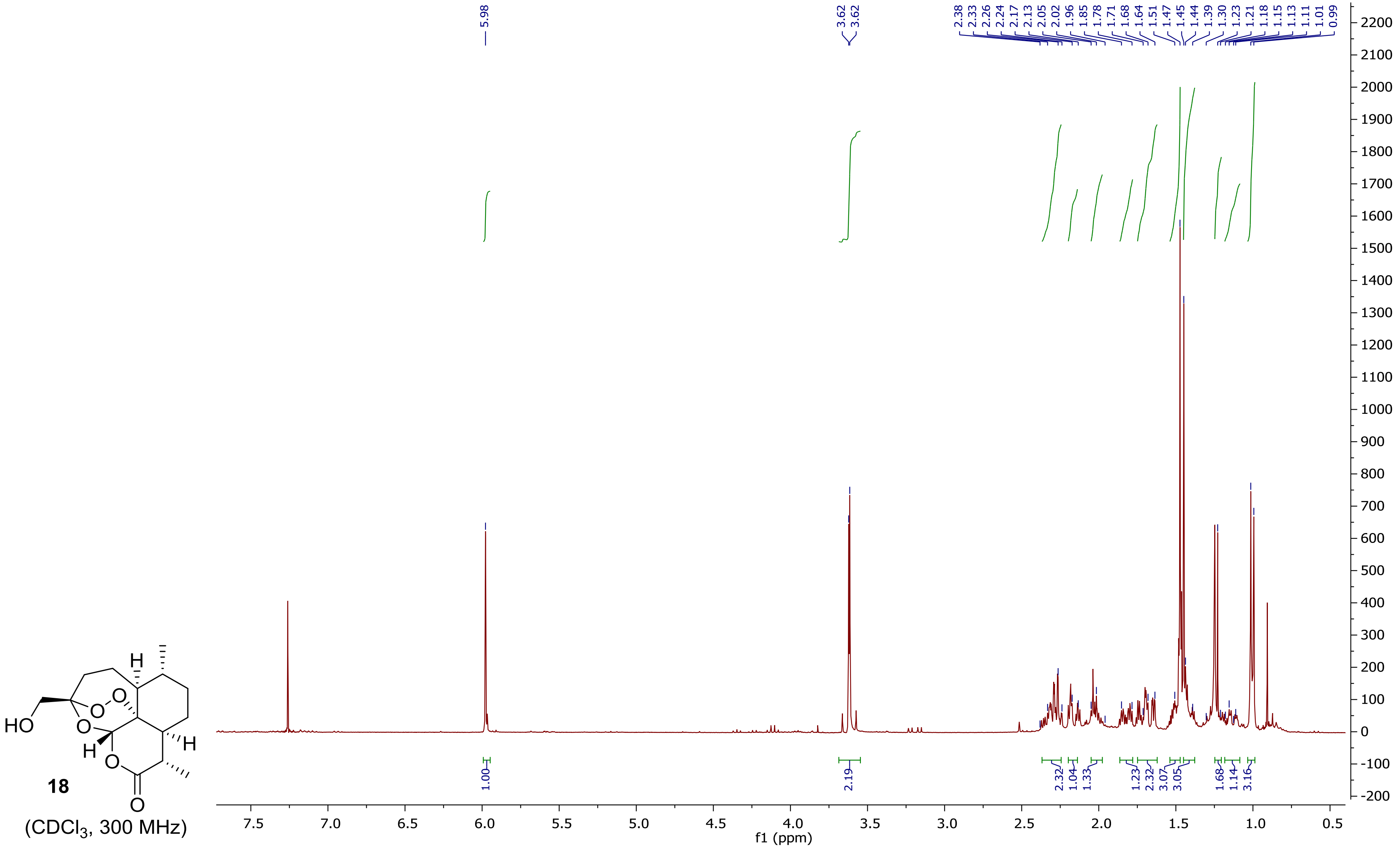

HO
18
(CDCl3, 300 MHz)
5.98
3.62
3.62
2.38
2.33
2.26
2.24
2.17
2.13
2.05
2.02
1.96
1.85
1.78
1.71
1.68
1.64
1.51
1.47
1.45
1.44
1.39
1.30
1.23
1.21
1.18
1.15
1.13
1.11
1.01
0.99
1.00
2.19
2.32
1.04
1.33
1.23
2.32
3.07
3.05
1.68
1.14
3.16
f1 (ppm)

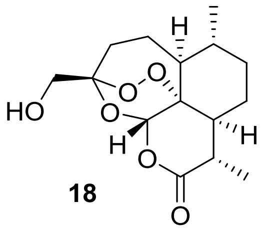

18

($CDCl_3$, 75.5 MHz)

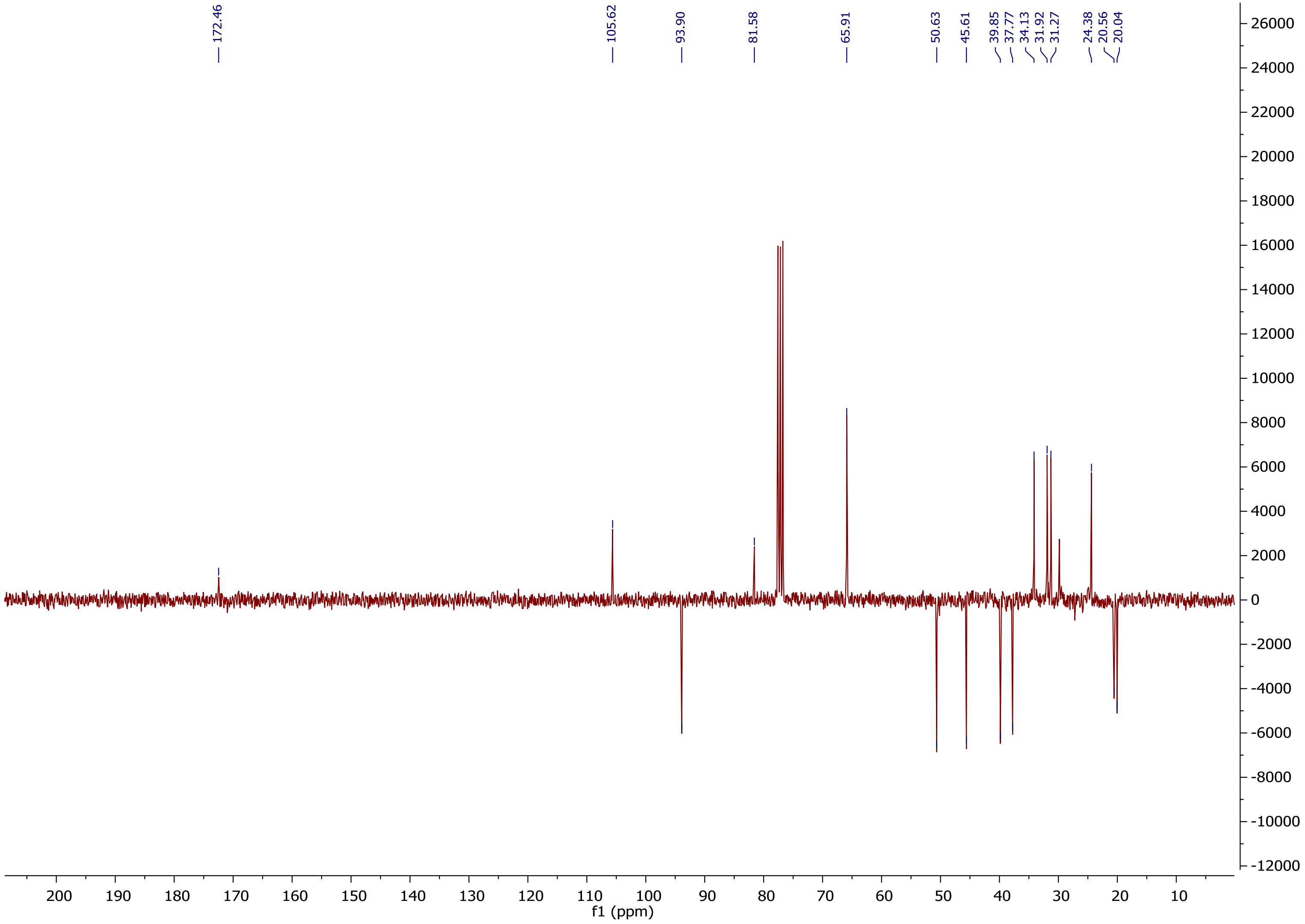

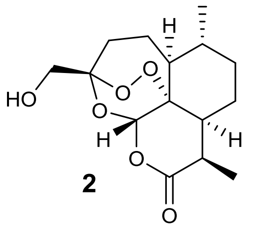

2

(CDCl$_3$, 400 MHz)

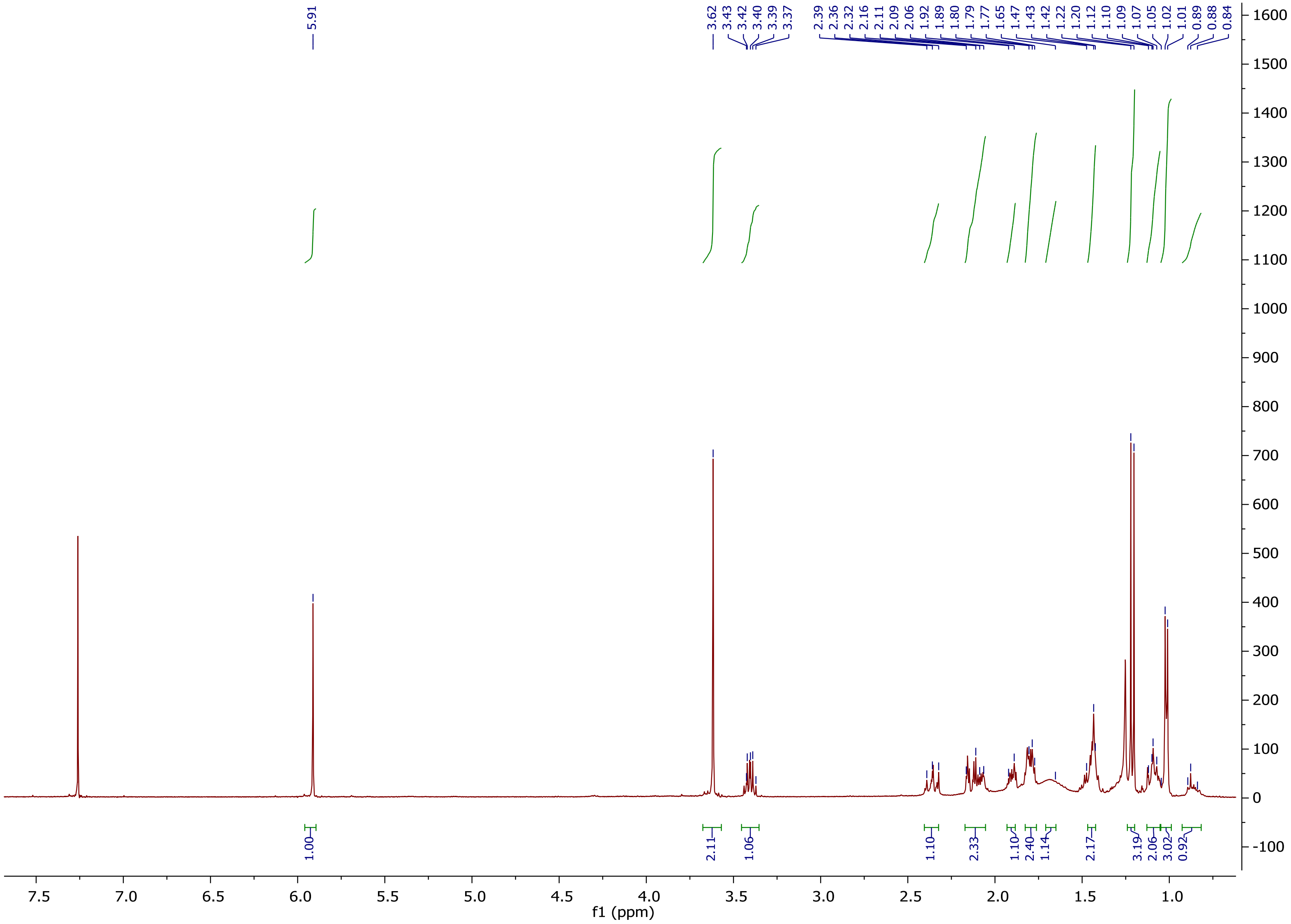

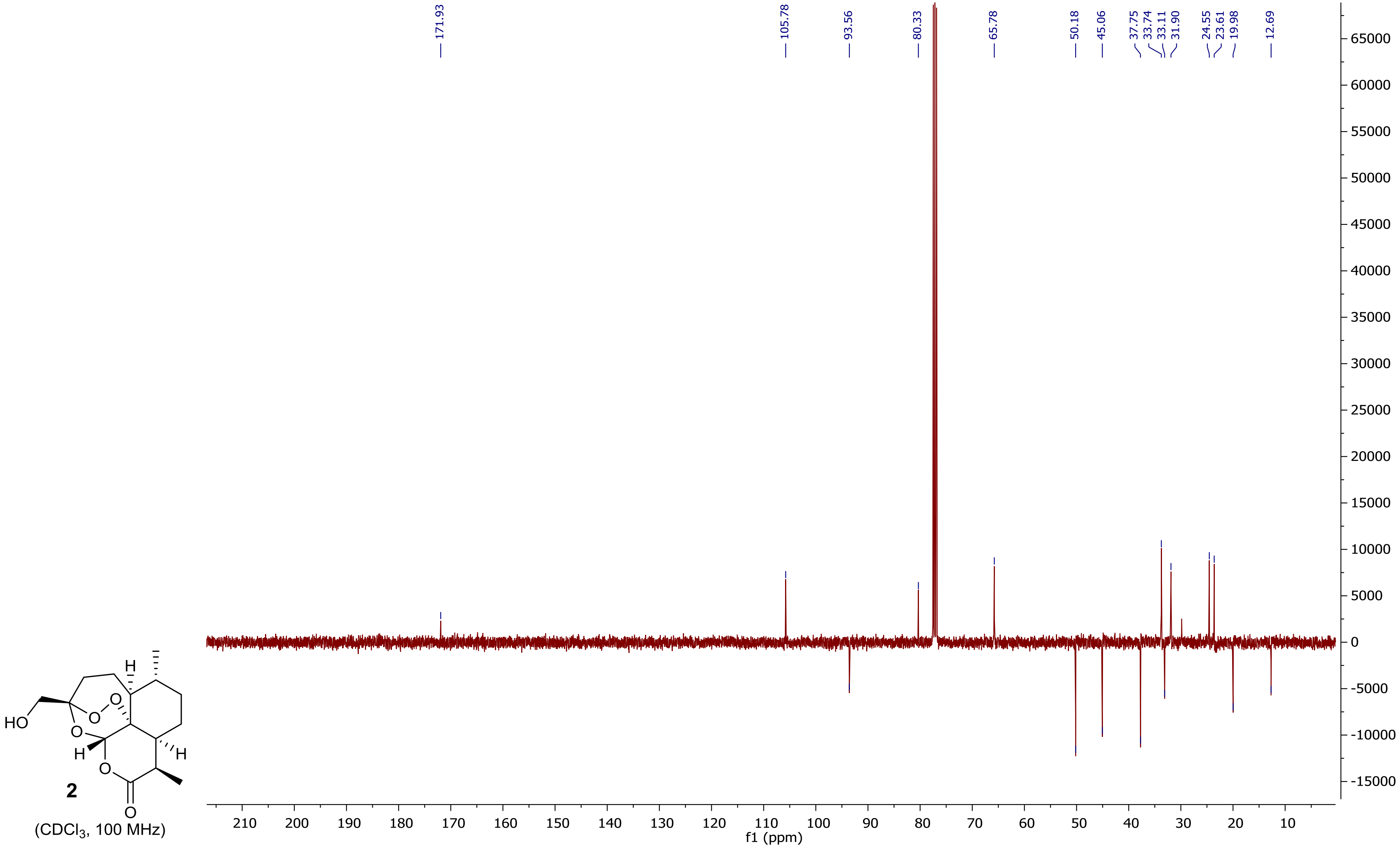
171.93
105.78
93.56
80.33
65.78
50.18
45.06
37.75
33.74
33.11
31.90
24.55
23.61
19.98
12.69
HO
O
H
2
(CDCl3, 100 MHz)
f1 (ppm)
